# Characterizing CO_2_ Reduction Catalysts on Gas Diffusion Electrodes: Comparing Activity, Selectivity, and Stability of Transition Metal Catalysts

**DOI:** 10.1021/acsaem.2c00160

**Published:** 2022-05-03

**Authors:** Mark Sassenburg, Reinier de Rooij, Nathan T. Nesbitt, Recep Kas, Sanjana Chandrashekar, Nienke J. Firet, Kailun Yang, Kai Liu, Marijn A. Blommaert, Martin Kolen, Davide Ripepi, Wilson A. Smith, Thomas Burdyny

**Affiliations:** † Materials for Energy Conversion and Storage (MECS), Department of Chemical Engineering, Delft University of Technology, 2629 ZH Delft, The Netherlands; ‡ Department of Chemical and Biological Engineering and Renewable and Sustainable Energy Institute (RASEI), University of Colorado Boulder, Boulder, Colorado 80303, United States

**Keywords:** CO_2_ reduction, gas diffusion electrode, catalyst comparison, silver, gold, palladium, tin, copper

## Abstract

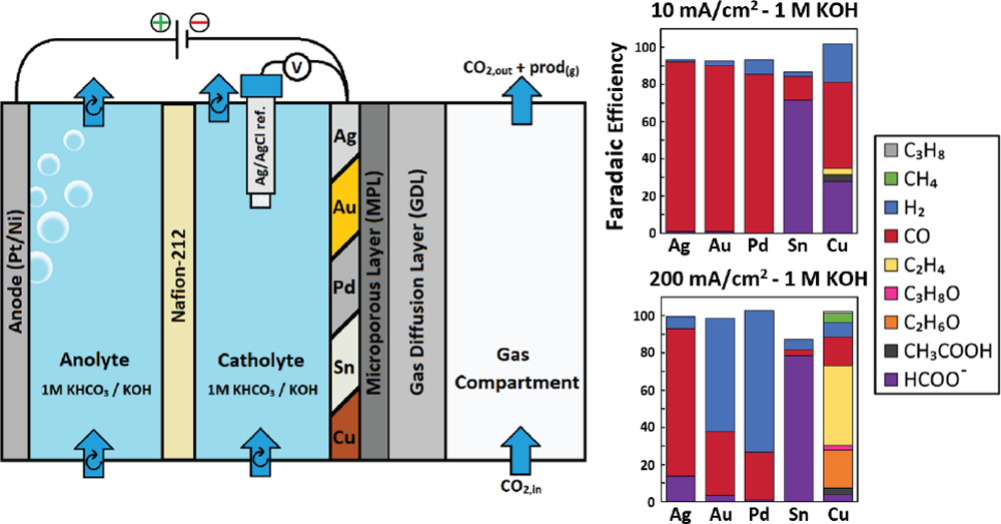

Continued advancements in the electrochemical reduction of CO_2_ (CO_2_RR) have emphasized that reactivity, selectivity, and stability are not explicit material properties but combined effects of the catalyst, double-layer, reaction environment, and system configuration. These realizations have steadily built upon the foundational work performed for a broad array of transition metals performed at 5 mA cm^–2^, which historically guided the research field. To encompass the changing advancements and mindset within the research field, an updated baseline at elevated current densities could then be of value. Here we seek to re-characterize the activity, selectivity, and stability of the five most utilized transition metal catalysts for CO_2_RR (Ag, Au, Pd, Sn, and Cu) at elevated reaction rates through electrochemical operation, physical characterization, and varied operating parameters to provide a renewed resource and point of comparison. As a basis, we have employed a common cell architecture, highly controlled catalyst layer morphologies and thicknesses, and fixed current densities. Through a dataset of 88 separate experiments, we provide comparisons between CO-producing catalysts (Ag, Au, and Pd), highlighting CO-limiting current densities on Au and Pd at 72 and 50 mA cm^–2^, respectively. We further show the instability of Sn in highly alkaline environments, and the convergence of product selectivity at elevated current densities for a Cu catalyst in neutral and alkaline media. Lastly, we reflect upon the use and limits of reaction rates as a baseline metric by comparing catalytic selectivity at 10 versus 200 mA cm^–2^. We hope the collective work provides a resource for researchers setting up CO_2_RR experiments for the first time.

## Introduction

Increasing energy demand has a significantly negative impact on the global environment because of the emissions associated with the extraction, transport, and utilization of fossil fuels. Renewable electricity generated from solar or wind and sustainable feedstocks such as air and water is needed to replace fossil fuels and reduce greenhouse gas emissions in the production of important chemicals and fuels. One promising approach can directly utilize atmospheric CO_2_ (or CO_2_ captured at point sources) and use renewable electricity to drive the electrochemical reduction of CO_2_ to valuable chemicals and fuels.

In the past decade, the CO_2_ reduction reaction (CO_2_RR) has received increasing attention due to its potential to supplant fossil fuels in the production of base chemicals and fuels. The field has built upon pivotal work in the 1990s and 2000s by Hori, which categorized the activity of transition metal catalysts for CO_2_RR under well-controlled conditions at a current density of 5 mA cm^–2^.^1^ These studies provided a solid foundation for exploratory catalyst development into each metal, giving the research field a fixed current density point of comparison. For CO_2_RR to be both economically feasible and environmentally impactful, however, significant progress is now needed to make the process efficient and stable at scale. In particular, large-scale facilities (> MW), high current densities (>100 mA cm^–2^), and long-term stability (>1000 h) with high energy efficiency and single pass conversion efficiency are needed to achieve these goals, while retaining near-uniform selectivity to reduce downstream separation processes.^2^ The necessity for process intensification in particular has now led to the rapid increase in current densities to the realm of 100–1000 mA cm^–2^, which significantly affects the local reaction environment,^3^ system design,^4^ catalytic behavior,^5^ and overall stability.^6^ The original controlled experiments characterizing materials at 5 mA cm^–2^ did not experience these consequences of process intensification, motivating the need for an updated reference of base performance of transition metal catalysts that reflect practical industrial conditions.

The use of gas diffusion electrodes (GDEs) has shown the ability to achieve high current densities (>200 mA cm^–2^) by having the catalyst supported on a microporous substrate at a gas–liquid interface.^7−11^ As the CO_2_RR community begins to use such electrode architectures that allow concentrated gas-phase CO_2_ to be fed close to the cathode, greater emphasis has been placed on understanding the interconnected factors, which govern the electrocatalytic performance as the scale and intensity of the system increases. While the electrode potential is ultimately the driving force that allows surface reactions to occur, the reaction environment is heavily influenced by current density and mass transport. For example, recent studies on catalysts deposited on GDEs have shown that an increase in current density^12,13^ and the use of different electrolytes^6,14−17^ have effects on product selectivity by varying the local reaction environment. The importance of current density dependent effects such as mass transport and homogeneous reactions is also observed in bicarbonate (KHCO_3_) electrolysis systems, where bicarbonate plays a dual role as a proton and CO_2_ source. A study on the direct conversion of a bicarbonate electrolyte (KHCO_3_) to CO for example showed that CO production was largely retained on a GDE while feeding nitrogen gas instead of CO_2._^18^ These examples highlight the importance of the catalyst’s surrounding reactor configuration on the measured performance and use fixed current densities to support previous work performed at fixed cathode potentials.

Another complexity within the field is that most reported studies do not describe the experimental setups that are used, and these setups can vary widely between groups as can the testing conditions that are used (e.g., flow rates, electrolyte, and membranes). Furthermore, few works present the data for multiple materials within the same paper as was previously done by Hori et al. at 5 mA cm^–2^. An updated baseline dataset of the most commonly used transition metals may then act as a reference for both new and established researchers in the field. In particular, a dataset where the experimental setup and the catalytic material had been well defined and compared against other catalysts under the same experimental conditions can provide a common foundation for benchmarking experimental setups.

**Figure 1:**
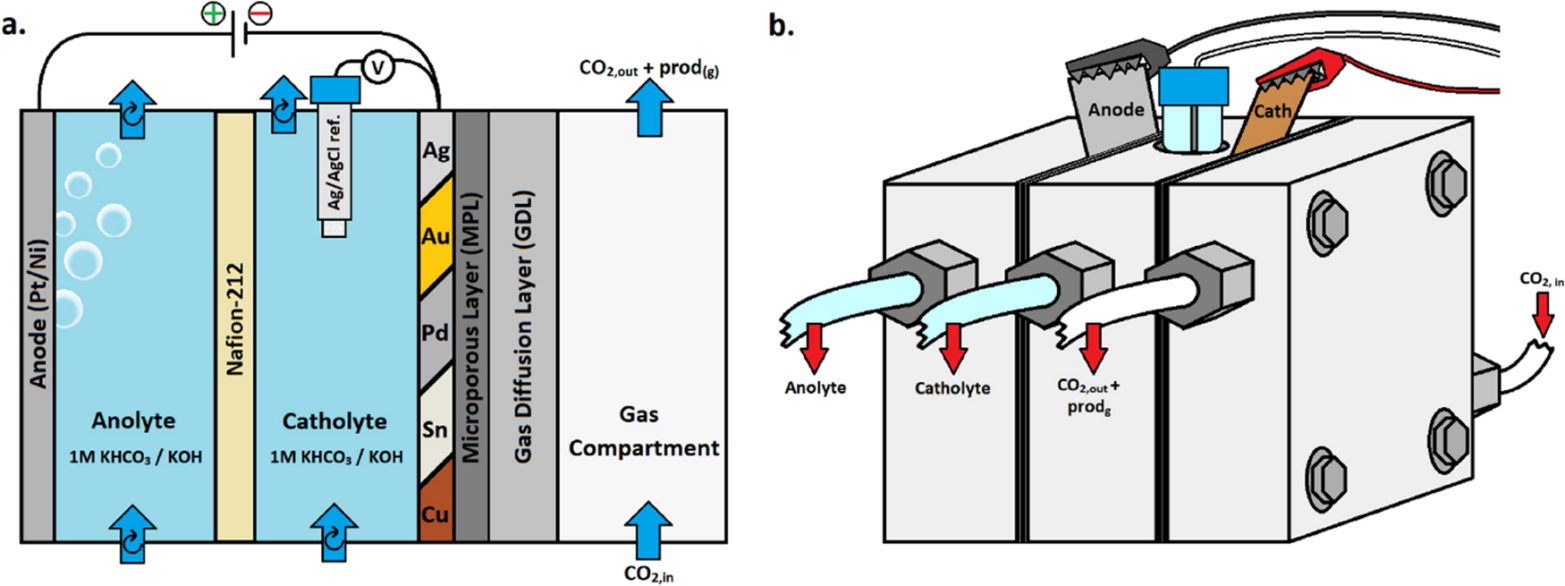
Schematic representation of the three-compartment GDE setup interior (a) and exterior (b) used to investigate CO_2_ RR catalysts in neutral and alkaline electrolytes while utilizing a gaseous CO_2_ feed.

In this work, we compare the baseline CO_2_ reduction performance of Au, Ag, Pd, Sn, and Cu catalysts deposited on GDEs at fixed current densities within a representative reactor configuration (Figure 1) over a broad parameter space. The electronic, structural, and electrochemical properties of the GDEs with different catalysts were characterized before and after 1 h of electrolysis using two electrolytes (1 M KHCO_3_ and KOH) and at four applied current densities (10, 50, 100, and 200 mA cm^–2^). Constant current densities were chosen instead of constant potentials in order to control the overall catalytic conversion rate, thus keeping the total diffusion and migration of the involved species comparable in each case. The dataset then supplements work using fixed cathodic voltages. During electrolysis at the applied current densities, the products were collected for analysis and the negative voltage was measured with respect to a Ag/AgCl reference electrode. The combined work provides a wide dataset for comparison with the literature, highlighting features of each of the metals which cannot be elicited from low current density experiments alone.

## Controlled Experimental Platform and Testing Conditions

For characterizing the electrochemical performance of the five transition metals, we have chosen to use a fixed cell architecture and catalyst morphology, which represents a recognizable baseline for the field. This entails a nanoparticle-based catalyst layer with a nominal catalyst thickness of 100 nm deposited onto a carbon-based gas diffusion layer with a flowing catholyte configuration (Figure 1a). Such an orientation is reflected in a number of publications within the field^19−24,16,25−27^ and such a system acts as a direct comparative baseline for research assessing changes in the type of gas diffusion layer, catalyst morphology, catalyst loading, electrolyte type, electrolyte concentration, and operating conditions (temperature, pressure, current density, and voltage). The chosen operating conditions for our comparisons span a range of current densities (10, 50, 100, 200 mA cm^–2^) for the two most commonly used electrolytes (1 M KHCO_3_ and 1 M KOH), thus encompassing common testing conditions in the literature.

While the configuration and operating conditions that are chosen for the dataset are important, we must make sure that their implementation is extremely well controlled to ensure both a high level of repeatability of the experimental data, as well as reproducibility of the results by external users. Without providing such regulation and documentation, the baseline cannot function as well as intended. Here, we provide large control over both the utilized catalyst, and the testing infrastructure as described below and in detail in the Supporting Information (SI).

To create a repeatable nanoparticle-based catalyst we deposited our five metal transition catalysts (Ag, Au, Pd, Sn, and Cu) onto a Sigracet 39BB gas diffusion layer (GDL) using magnetron sputtering (AJA International Inc.) to deposit ∼100 nm thick metal catalysts (see detailed description and equipment in SI A). The deposition thickness of the unit was confirmed through profilometry for each individual material. The as-deposited samples then resulted in a nanoparticle layer on the top of the GDL, which was similar for each base material as confirmed though scanning electron microscopy (SEM) (JSM-6010LA, JEOL), high resolution SEM imaging (NovaNanoSEM 450, FEI), and atomic force microscopy (AFM) (AFM with Icon ScanAsyst, Bruker). The five materials are visualized in Figure S21, exhibiting a similar porous structure. Because of the roughness of the GDE and the catalyst layer porosity, the thickness is greater than the deposited 100 nm. The elemental composition of the catalyst surface was examined ex situ by X-ray photoelectron spectroscopy (XPS) (K-Alpha, Thermo Scientific) before and after electrolysis to identify the surface species present on the electrolyte side of the catalyst layer. Since XPS is performed ex situ, a measure of oxidation from air is expected for surface species for all samples. SEM and XPS analyses allow for the stability of the catalyst layer to be examined from a morphology and contaminant perspective. In order to minimize the influence of residual electrolyte species on the ex situ SEM and XPS results, a rinsing protocol with DI water and drying was included (see SI A). All catalysts were deposited homogeneously on a 4.4 cm^2^ square electrode area, with a geometric active electrode area of 2.25 cm^2^ exposed to the electrolyte while placed in the assembled electrochemical cell. Lastly, a new sample was used for each electrochemical experiment.

While control over the catalyst deposition and morphology is of critical importance, so too is the robustness of operating the electrochemical system itself. Operating GDE systems for CO_2_ electrolysis is challenging for a number of reasons relating to electrode flooding,^28,29^ penetration of CO_2_ into the liquid phase, CO_2_ consumption by the electrolyte,^30^ and pressure-imbalances caused by fluid flow and gas chromatography (GC) measurements. Here, we demonstrate a system, which incorporates back-pressure regulation (to prevent gas/liquid crossover) and mass flow meters (to identify the gas flow into the GC used in calculations) to maintain the gas–liquid environment as consistently as possible during experiments. GC measurements every 5 min lead to some gas pressure increase and gas escaping through the liquid phase, but only after injection of the product gas stream. To improve the confidence in the presented results, duplicates of each experiment were performed.

**Figure 2:**
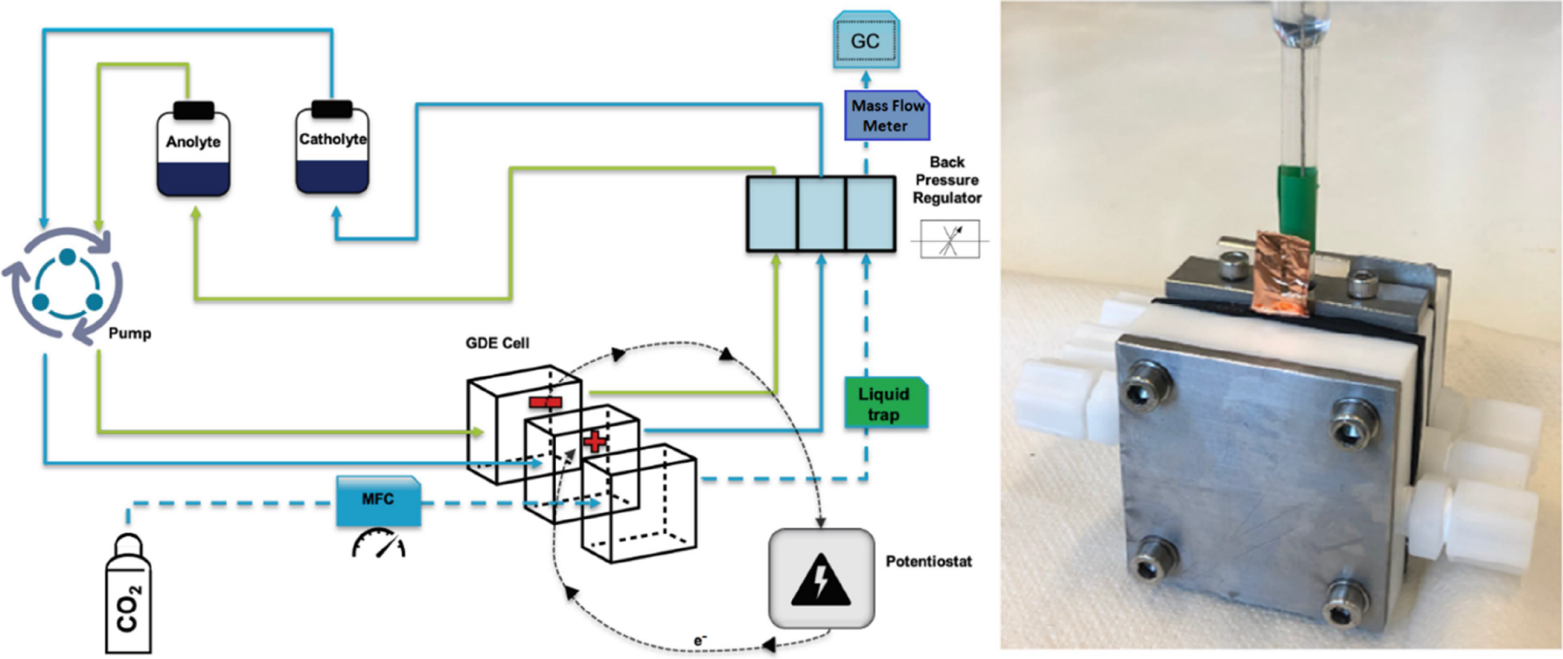
Left: schematic drawing highlighting the components of the electrochemical setup. Right: picture of the assembled 3-compartment flowcell.

Electrochemical experiments were performed in a three-compartment GDE system as shown in Figure 2. Technical drawings of the cell compartments are available in the SI (Figure S57–S60). The electrochemical setup consists of external liquid bottles containing 80 mL each of the respective anolyte and catholyte connected to a peristaltic pump to recirculate the catholyte and anolyte chambers at 10 mL min^–1^. It is important to note that the recirculation of the electrolyte could induce transient pH effects due to a combination of continuous acidification by CO_2,g_ reacting with hydroxyls and the production of hydroxyls at the cathode. In general, a KHCO_3_ bulk pH shift from 7.8 to 8.5–8.8 (at 200 mA cm^–2^, 1 h) was measured for KHCO_3_. For KOH, the dissolution of CO_2_ was a more significant factor reducing bulk pH from 13.8 to 13.0–12.8. CO_2_ was provided using a pure CO_2_ bottle and regulated by a mass flow controller to feed the cathode gas compartment at 30 mL min^–1^. The electrochemical measurements were performed utilizing a ParSTAT MC potentiostat (Ametek SI) to perform 1-hour chronopotentiometry on each sample. The electrochemical cell includes a Ag/AgCl reference electrode, positioned in the catholyte chamber to measure cathodic potential. A liquid trap at the gas outlet of the cell is used to protect the GC in case of flooding. All outlets are connected to a back-pressure regulator and enable the balancing of gas and liquid pressures at 1220 mbar, hereby promoting gas/liquid separation. The quantity of gas entering the GC was measured again using a mass flow meter (Bronkhorst EL-FLOW Select), since the conversion and dissolution of CO_2_ can lead to great disparity between the in- and outflow. The products of electrochemical CO_2_ reduction over 1 h were measured using online GC (Compact GC 4.0, Global Analyzer Solutions).

While gas products (CO, CH_4_, C_2_H_4_, and H_2_) were measured by online GC, post experimental analysis of the accumulated liquid products in the catholyte (formate, ethanol, and propanol) were performed using an Infinity 1260 II HPLC (Agilent Technologies). A Nafion-212 membrane was deployed to prevent anionic products from crossing over to the anolyte. As validation, anolyte samples were taken from experiments in which large quantities of formate were expected to be formed. During analysis of the anolyte, product signals were less than 0.5% of the catholyte signal and were thus discarded. Data of product analysis and the electrochemical experiments were combined to show the faradaic efficiency (FE) and partial current density of the products as a function of applied current density, providing two different perspectives of the same data.

The electrode potentials versus a Ag/AgCl reference were also recorded during experiments and converted to the RHE, but were not iR-corrected. In the system configuration, a large ohmic drop exists, which reduces the accuracy of the iR-correction, particularly because of changes in the electrolyte conductivity with current density,^44^ temperature, and experimental time (see SI A. EIS for further details).

More detailed information on the fabrication of electrodes, measurement equipment, and followed procedures can be found in SI A. Protocol. The following sections then provide the detailed structural properties and electrochemical performance of the five metal catalysts (Ag, Au, Pd, Sn, and Cu) deposited on GDEs over the examined operating range.

## Results

Here, we present the material and electrochemical characterization for the five most commonly investigated transition metal catalysts (Ag, Au, Pd, Sn, and Cu) for CO2R. For each of the five metals and two electrolytes and duplicates of four current densities were tested. In some cases, extra experiments were added to extend observed trends (for Au/Pd) and where stability issues were observed (for Sn). In this work, 88 GDE samples were then fabricated and characterized with chronopotentiometry, product analysis, SEM imaging, XPS, and with that a substantial dataset was obtained. For the sake of brevity, only the most relevant data are presented in the following sections, with the most critical findings given greater emphasis. All obtained data are available in SI B. Characterization Data, categorized by characterization technique, for use in further studies and comparisons.

While all electrochemical experiments were run for 1 h, the data presented here use the selectivity versus current density after the first 20 min of operation, averaged over the duplicate experiments. This time was chosen as it simultaneously allowed for the stabilization of product curves from the GC, and does not conflate catalyst stability over time with the selectivity of the original catalyst and configuration (e.g., Sn dissolution over time). The stability of the catalyst over the full-length of experiments is, however, discussed.

### Silver

Silver (Ag) is a promising electrocatalyst for the selective conversion of CO_2_ to CO and has previously been studied in H-cells^31−34^ as well as in GDE architectures.^35,16^ The selectivity of Ag to produce CO from CO_2_ is largely due to the weak binding energy that CO has with Ag surfaces, though there are minor differences with facet/site composition and coordination. A recent study found that 20–30% of the selectivity of Ag toward CO can be tuned toward formate (HCOO^–^) by increasing the pressure and electrolyte alkalinity without affecting the catalyst stability.^36^ When this work was compared to other Ag-GDE studies, it showed that CO/HCOO^–^ selectivities and energy efficiencies at equal current densities were nonuniform across separate studies, implying the presence of unique parameters for each configuration.

**Figure 3:**
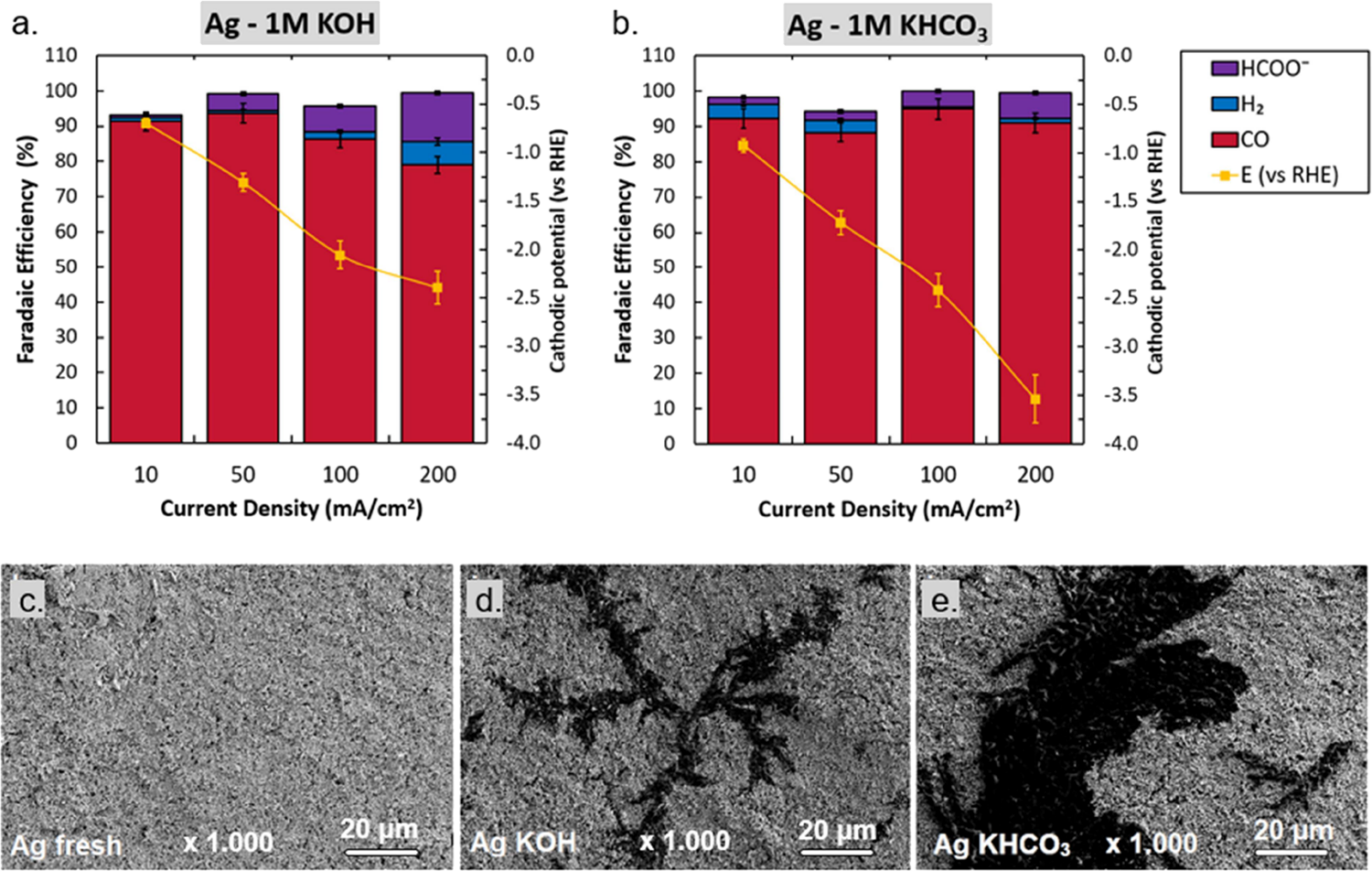
Characterization of Ag coated electrodes. Faradaic efficiency as a function of activity with cathodic potentials in 1 M KOH (a) and 1 M KHCO_3_ (b). Error bars in panels a and b represent the data points from two separate experiments. SEM images before (c) and after both 200 mA cm^–2^ experiments (d, e) show dark surface coverages.

In our work, for all tested current densities, Ag shows >80–90% selectivity toward CO with a gradual shift toward formate as the current density increased (Figure 3a,b). The HCOO^–^ formation increasing at higher current densities has been previously reported to be an effect of high local pH, which favors HCOO^–^ formation at the expense of CO.^37^ Despite the high selectivity of these electrodes, the surface morphology exhibited significant changes in both electrolytes after 1 h of electrolysis. SEM images (Figure 3c–e) show that after electrolysis in either electrolyte, large (>20 μm) features emerge in fractal-like structures, indicating that electrons are being scattered or absorbed in greater amounts. XPS characterization of these features primarily shows potassium and oxygen, suggesting that they may be related to salt formation from the electrolytes during or after operation. Ion beam etching was performed on these electrodes, and showed pristine Ag under this top layer of K/O (see SI B: HR-SEM). Although the selectivity was minimally affected during the 1 h experiments, continuation of electrolysis under these conditions will likely lead to large salt crystals forming on the surface, eventually blocking gas flow and/or rupturing the substrate. A recent paper on the surface coverage and the effects of electrolyte concentration on K-salt growth has shown similar features which are directly attributed to be potassium carbonate (K_2_CO_3_) and resulted in a rapid decrease of selectivity to CO after 50% of the surface was covered.^38^

From the performed experiments, it can be concluded that Ag is an effective CO producing electrocatalyst with high selectivity and low overpotential compared to other catalyst materials presented here. Such a result is not unexpected given silver’s prevalence in GDE-based CO2R. Overall, the selectivity for Ag to CO was retained over the evaluated current density range. Increasing the current density to 200 mA/cm^2^ caused the local reaction conditions to become more alkaline over time, promoting the production of formate.

### Gold

Gold (Au) has historically been shown to be the best performing CO reduction catalyst in aqueous based H-cells due to its low onset potential for the CO_2_RR and high selectivity toward CO.^39−42^ Although Au has shown the ability to lower the initial energy barrier in the CO2RR, increasing current densities above the H-cell regime show a continuous loss of selectivity toward CO while H_2_ evolution is promoted. Subsequently, gold is comparatively un-utilized in GDE configurations compared to H-cell systems. In examples where gold has been used in GDEs, low partial current densities toward CO are observed before the hydrogen evolution reaction begins to dominate.^43^

**Figure 4:**
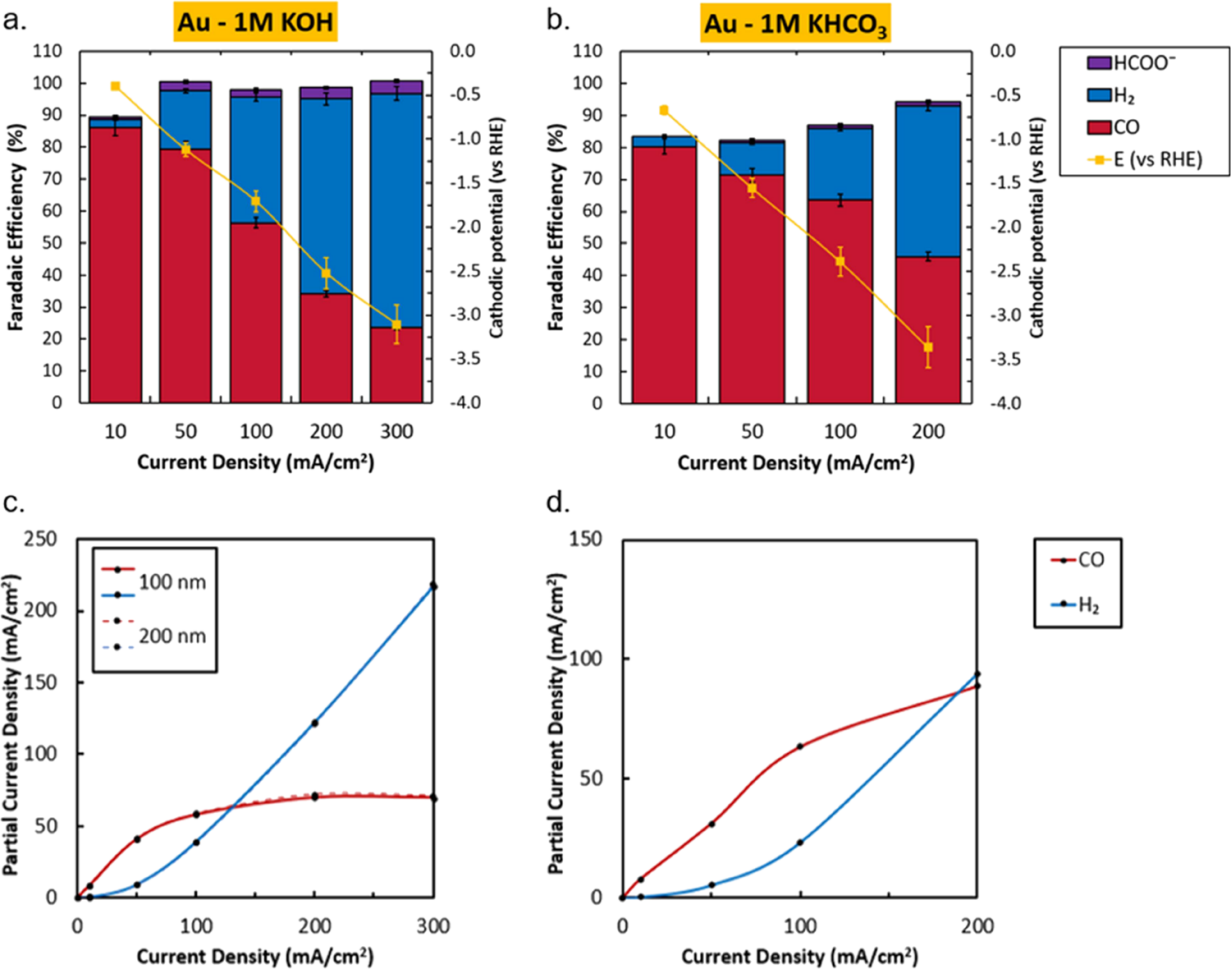
Characterization of Au coated electrodes. Faradaic efficiency as a function of activity with cathodic potentials in 1 M KOH (a) and 1 M KHCO_3_ (b). Error bars in panels (a) and (b) represent the data points from two separate experiments. Correlated partial current density for 1 M KOH on 100 and 200 nm Au (c) shows a limiting CO current density of 72 mA cm^–2^. Partial current density of Au in 1 M KHCO_3_ (d) can be seen to level off at a slightly higher value. Blue and red lines are added to visualize the limiting trend of CO and the gradual increase of H_2_.

Within our experimental dataset, we observe a similar limitation from the gold catalysts. In particular, the 1 M KOH experiments depict a clear downward trend in CO selectivity with increasing current density which occurs earlier than the mass transport limited currents achievable. Plotting the same data as a partial current density instead (Figure 4c), it can be seen that the CO production rate becomes limited to *j*_CO_ = 72 mA cm^–2^. In experiments conducted in KHCO_3_, CO also begins to plateau in the tested range. Comparing material characterization before and after the reaction, XPS scans (Figures S41–S43) show no changes in Au peak intensity, and SEM images show no mesoscopic changes to the surface. However, post-experimental XPS results do show peaks for potassium (K 2p) and oxygen (O 1s), due to the formation of (bi)carbonate on the catalyst surface similar in nature to the peaks observed for the Ag catalyst, but in lower quantities.

Aside from the decaying selectivity toward CO, the most interesting Au result is the observed limiting current density of 72 mA cm^–2^ in 1 M KOH. To assess whether the limitation was due to surface site availability, we doubled the sample thickness to 200 nm nominal thickness and tested over the same range of current densities. At this thickness, the entire catalyst layer should still have ample access to CO_2_. However, these 200 nm samples showed near identical results to the thinner 100 nm samples (see dotted line in Figure 4c). Similar studies on pure Au (with different parameters) resulted in limiting current densities of *j*_CO_ = 35 mA cm^–2^^13^ and to 100 mA cm^–2^.^43^ Further research is required to determine whether this limitation toward CO is intrinsic to Au and to better understand which conditions might affect the value of the plateau current.

### Palladium

**Figure 5:**
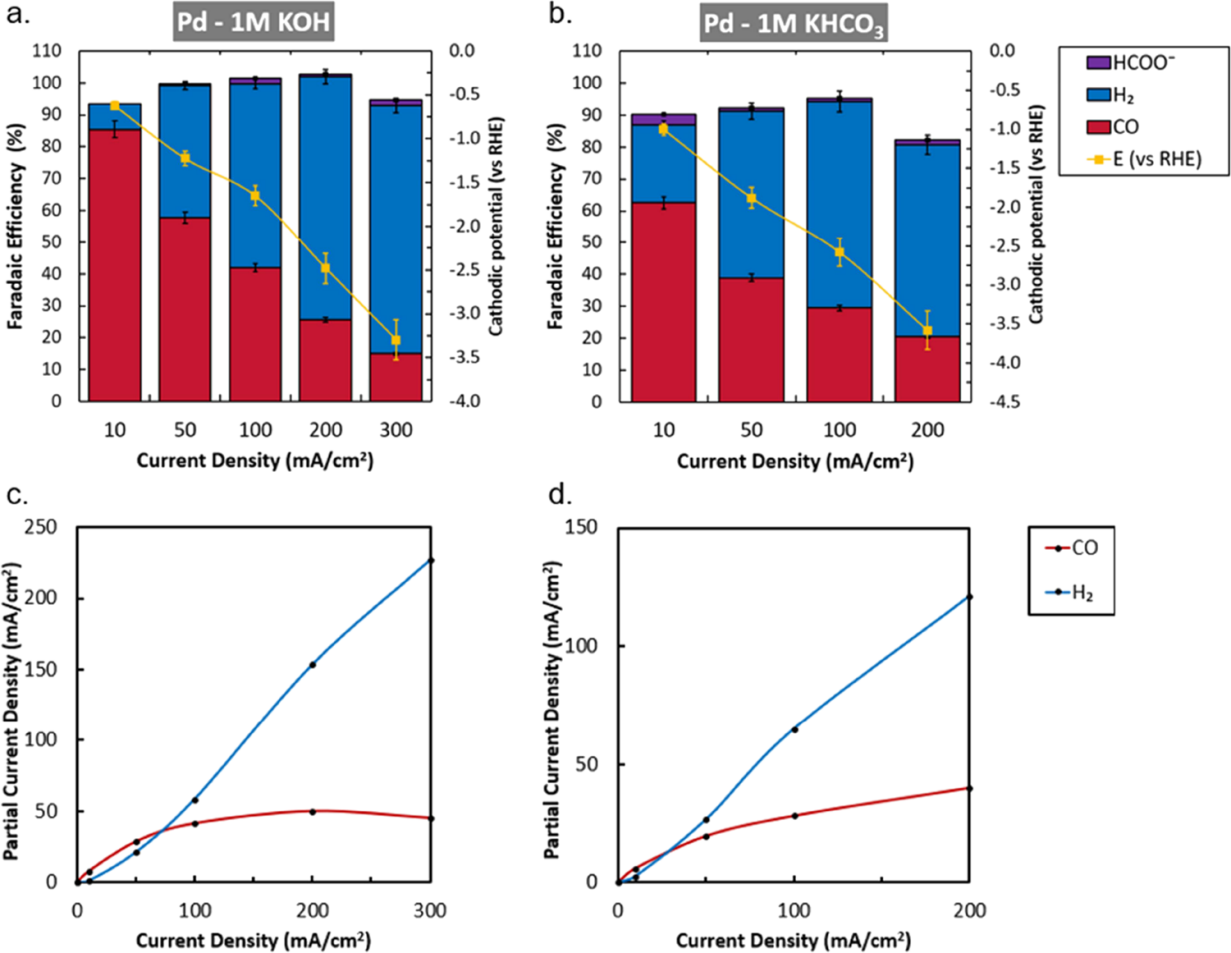
Characterization of Pd coated electrodes. Faradaic efficiency as a function of activity with cathodic potentials in 1 M KOH (a) and 1 M KHCO_3_ (b). Error bars in panels (a) and (b) represent the data points from two separate experiments. Correlated partial current density for Pd in 1 M KOH (c) show a limiting CO current density around 50 mA cm^–2^. Partial current density of Pd in 1 M KHCO_3_ (d) levels off at a slightly lower value while the HER continually increases. Blue and red lines are added to visualize the limiting trend of CO and the gradual increase of H_2_.

Palladium (Pd) has been studied as a single crystal electrode for CO_2_ reduction^45^ and as a nanoparticle catalyst^46−48^ in which CO and formate were found as the main carbon containing products at different electrode potentials. In our experiments shown in Figure 5, Pd exhibits high initial selectivity toward CO at 10 mA cm^–2^, but shows steadily increasing HER selective behavior as a function of current density, similar to what was seen for Au, and only trace amounts of formate. Minimal amounts of formate were found for all the experiments across the entire applied current range. Similar to what was observed for gold, the partial current densities indicate a limiting current density to CO of approximately 50 mA cm^–2^. XPS results reveal that during the experiments, potassium accumulates on the surface accompanied by a slight decrease in the Pd 3d signal, indicating partial coverage. Before electroreduction, the Pd catalyst already showed oxygen content comparable to after the experiment, however, a peak shift toward slightly lower binding energies is witnessed after both KOH and KHCO_3_ experiments, indicating a change in the role of oxygen. SEM images before and after applying current seem to be relatively stable for KHCO_3_, except for localized impurities. Additional SEM images for the electrodes operating in KOH at intermediate current densities (especially at 100 and 200 mA cm^–2^, see SI B) show a wide variety of drastic morphological surface changes.

Unlike Au, previous literature performed at lower current densities suggests that Pd experiences a plateau current density for CO due to surface poisoning by CO at lower overpotentials.^49−51^ Here, at the elevated operating potentials, it is, however, unclear if this is limiting its performance. From the results here, the overall high level of H_2_ formation and the relatively large required overpotentials make pure Pd nanoparticles an inadequate catalyst for large-scale utilization in its present form. Alternatively, Pd might find its use as a bimetallic co-catalyst, as past studies have shown it to be an interesting metal to tune dimerization to multicarbon products^52,53^ due to its strong binding with CO.

### Tin

Tin (Sn) is a catalyst studied for its highly selective formation of formate.^55−59^ Finding a highly selective formate (HCOO^–^) catalyst can be helpful for the development and implementation of CO_2_ reduction technologies. Alongside CO, formate is another chemical building block that can be used as a reactant in further downstream synthesis, but can also be used as a renewable feedstock in biosynthesis toward fine chemicals.^54^ Sn does suffer from poor stability, leading some researchers to investigate alloying and adding ionomers and binders to protect the Sn catalyst.^60−63^

**Figure 6:**
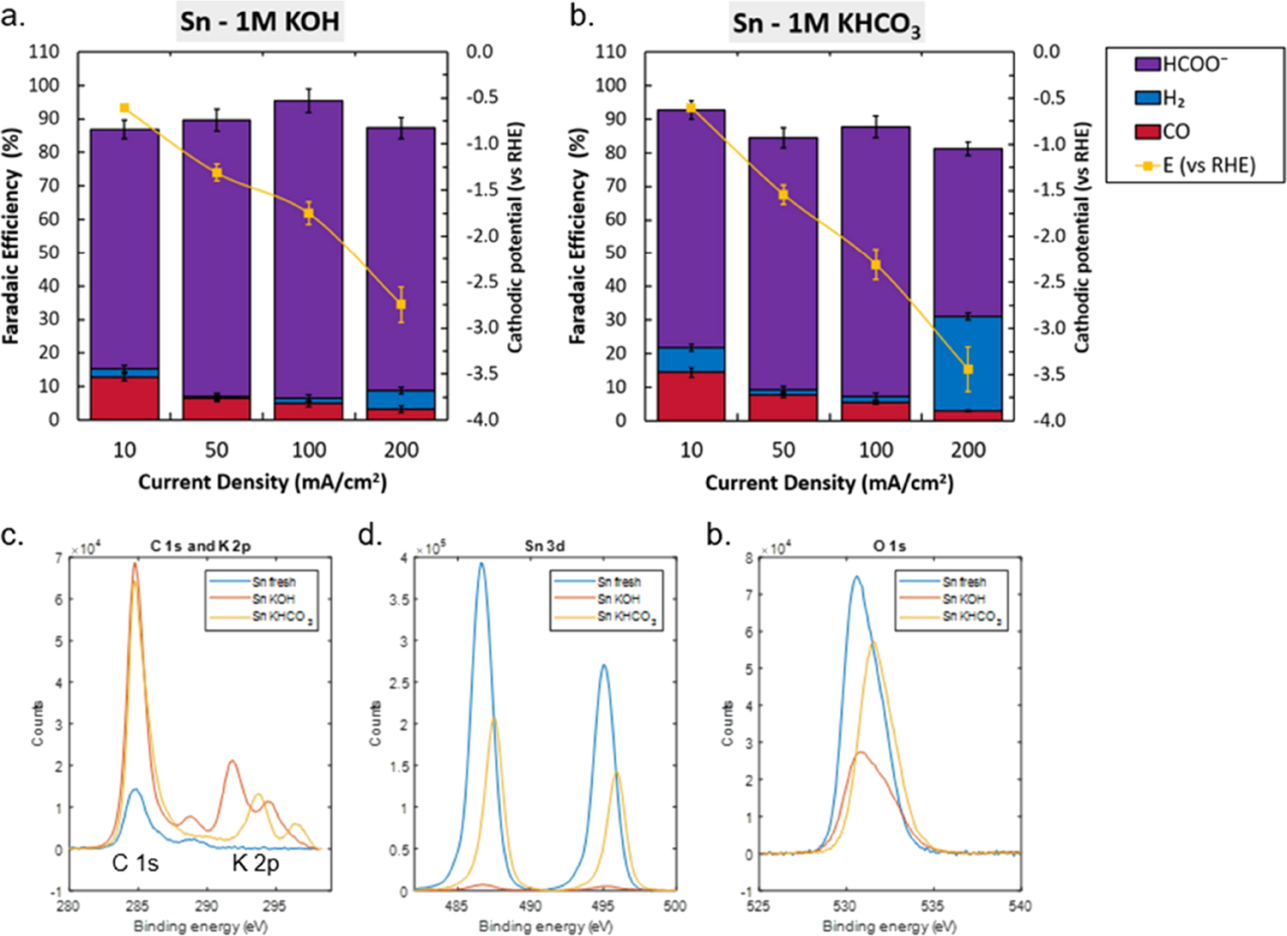
Characterization of Sn coated electrodes. Faradaic efficiency as a function of activity with cathodic potentials in 1 M KOH (a) and 1 M KHCO_3_ (b). Error bars in panels a and b represent the data points from two separate experiments. XPS results for C 1 s and K 2p (c), Sn 3d (d) and O 1 s (e) scans before and after the 200 mA cm^–2^ experiment in both electrolytes.

Here, GDEs deposited with Sn show high selectivity toward formate throughout all experiments across the entire applied current range. At an applied current density of 200 mA cm^–2^, the system lost selectivity toward carbon containing products, reflected by the increase in hydrogen evolution over the duration of the experiment. An explanation for this is provided by observing the XPS spectra, where a scan of the Sn 3d peaks shows a significant decrease of Sn after the KHCO_3_ experiment and near-complete disappearance after 1 h operation in KOH, indicating the loss of Sn during electrolysis due to dissolution in the highly alkaline conditions, as described by the Sn Pourbaix diagram. The O 1 s peaks also show decreased signal, following the trend of Sn 3d. Potassium uptake is relatively low for these samples, as is displayed by less prominent K 2p peaks (right peaks of Figure 6c). SEM images of Sn samples after reaction in KHCO_3_ show no clear morphological changes, aside slight bleaching near GDL native cracks (as shown in zoomed out SI images). Crystals (different from the earlier seen (bi)carbonate) were also found in KOH experiments. These crystals were likely formed by a combination of the dissolution of Sn in the highly alkaline environment, while the applied potential caused localized redeposition in a more stable agglomerated form.

Overall Sn has shown to be an effective catalyst for the selective production of HCOO^–^ throughout many years of prior research, and this trend is confirmed here. However, the lack of stability at elevated current densities of a sputter deposited Sn catalyst showed that it is vital to find techniques to stabilize the catalyst and prevent the Sn dissolution through the use of nanoparticles, binding agents, co-catalysts, or ionomers in order to ensure long-term stability.

### Copper

Copper (Cu) has received significant attention by CO_2_ reduction researchers due to its unique ability to convert CO_2_ into at least 16 different products.^64^ Numerous studies focused on improving the activity and selectivity of Cu through morphological enhancements,^65,66^ facet-dependent behavior,^67,68^ and local environment control.^69,70^ Some of the mechanistic pathways behind the formation of various products are still debated,^71,72^ but it has become clear that the specific binding strength of Cu to CO allows for the dimerization of adsorbed CO* and CHO* species, resulting in multicarbon (C_2+_) product formation. In GDE experiments, Cu and Cu-alloys have shown promising selective behavior toward prominently ethylene at elevated current densities.^73^

**Figure 7:**
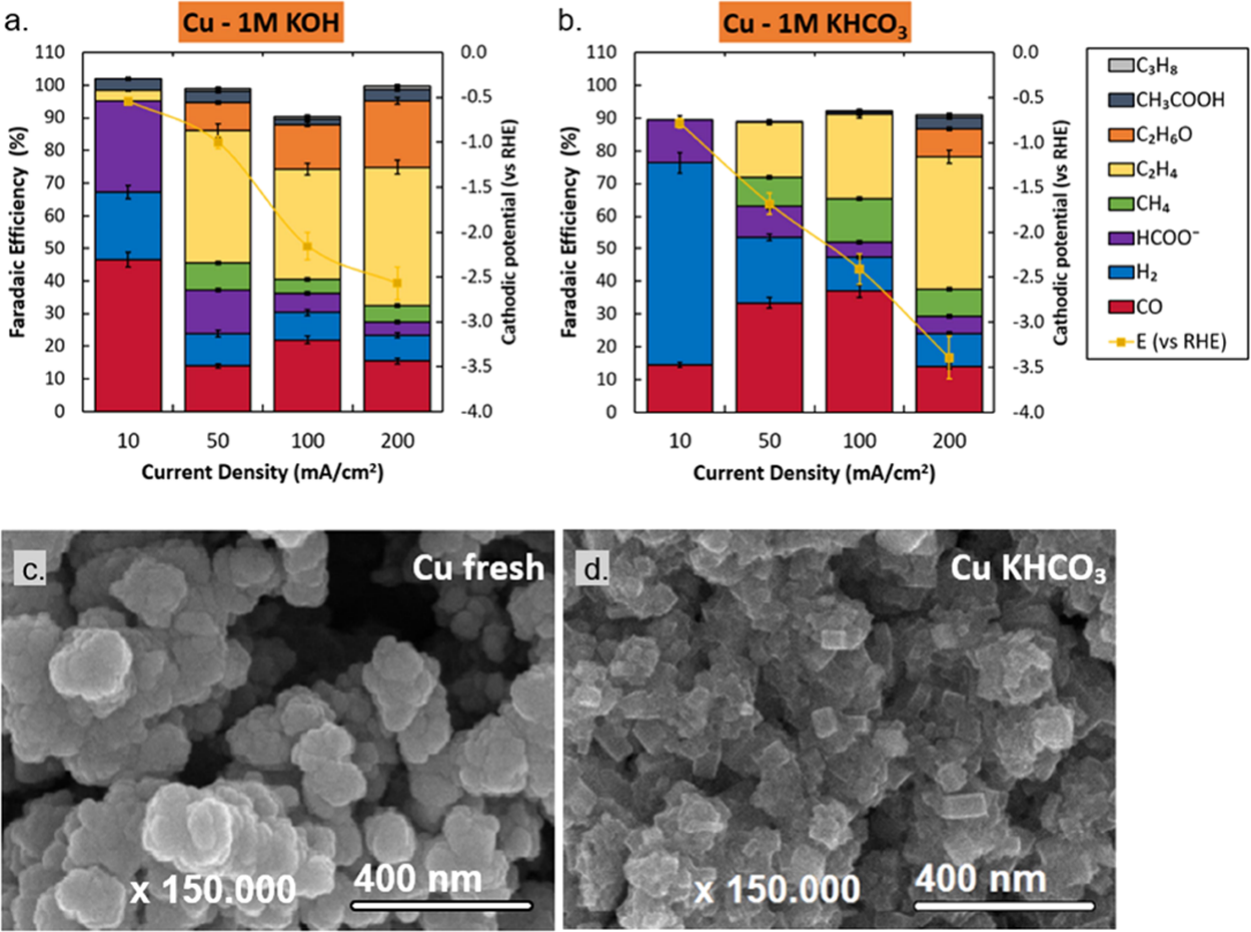
Characterization of Cu coated electrodes. Faradaic efficiency as a function of activity with cathodic potentials in 1 M KOH (a) and 1 M KHCO_3_ (b). Error bars in panels (a) and (b) represent the data points from two separate experiments. HR-SEM images of fresh Cu (c) and after 1 h electrolysis in KHCO_3_ (d) show a cubic faceting of the catalyst.

In our work, sputter-deposited Cu GDEs show highly varied product selectivities with changing current densities, as reported elsewhere. At an applied current density of 10 mA cm^–2^, the Cu GDEs produce a mixture of H_2_, CO, and formate at low overpotentials. At an applied current density of 50 mA cm^–2^, methane, ethylene, and ethanol are detected as well. Further increasing the current density shows a shift in the product distribution toward ethylene while CO production plateaus. Comparing XPS results before and after experiments show that, besides a slightly higher degree of oxidation and the presence of potassium in the case of KOH (while decreasing the Cu 2p signal), the composition remains consistent. In contrast, SEM imaging does show significant restructuring of the surface in most experiments. The 200 mA cm^–2^ case shows that the specific conditions and applied potential resulted in the formation of Cu nanoneedles and cubes. The post-electrolysis HR-SEM image of KHCO_3_ (Figures 7d and S37) shows that the Cu catalyst has restructured under the applied potential, favoring cubic shapes.

### Comparison of Low and High Reaction Rate Selectivities

Through the presented experiments, we were able to observe trends for the different catalysts as a function of applied current density. Of the five assessed transition metals, only silver maintained its selectivity toward CO_2_ reduction products over a broad current density range, while Au, Sn, and Pd tended toward H_2_ as a primary product as current densities were increased. Cu maintained its total CO_2_ reduction selectivity, with product distributions growing at higher reaction rates. These results highlight how low versus high current density testing conditions change the observed product selectivities through variations in the local reaction environment, changes to catalyst stability, and the increased applied potentials which influence the relative activity of each product at different current densities.

One observation that needs to be highlighted is the limiting current density of Au and Pd toward CO, whereas Ag did not exhibit such a limit under the same conditions. Here the production of H_2_ on Ag remains low up to 200 mA cm^–2^, allowing for high CO selectivities to be maintained. Conversely on Au, hydrogen formation increases with current density while CO plateaus. A detailed study into the intrinsic limit of converting CO_2_ could help determine which of these metals can effectively be used for industrial purposes, and why gold is a less favorable CO_2_R catalyst at higher potentials and current densities.

For Sn the effect of electrolyte composition was more impactful than current density in the conversion of CO_2_ to formate. We found that Sn experiments in KHCO_3_ lead to mild catalyst restructuring, while in KOH structural instabilities damaged the catalyst surface irreversibly. During the 1 h electrolysis the effects of restructuring were not clearly expressed through product distributions yet, but it became apparent that increasing current densities led to enhanced surface reformation and more frequent flooding issues. These trends highlight the necessity of applied current density and electrolyte composition when comparing or benchmarking the electrochemical performance of catalysts on GDEs.

**Figure 8:**
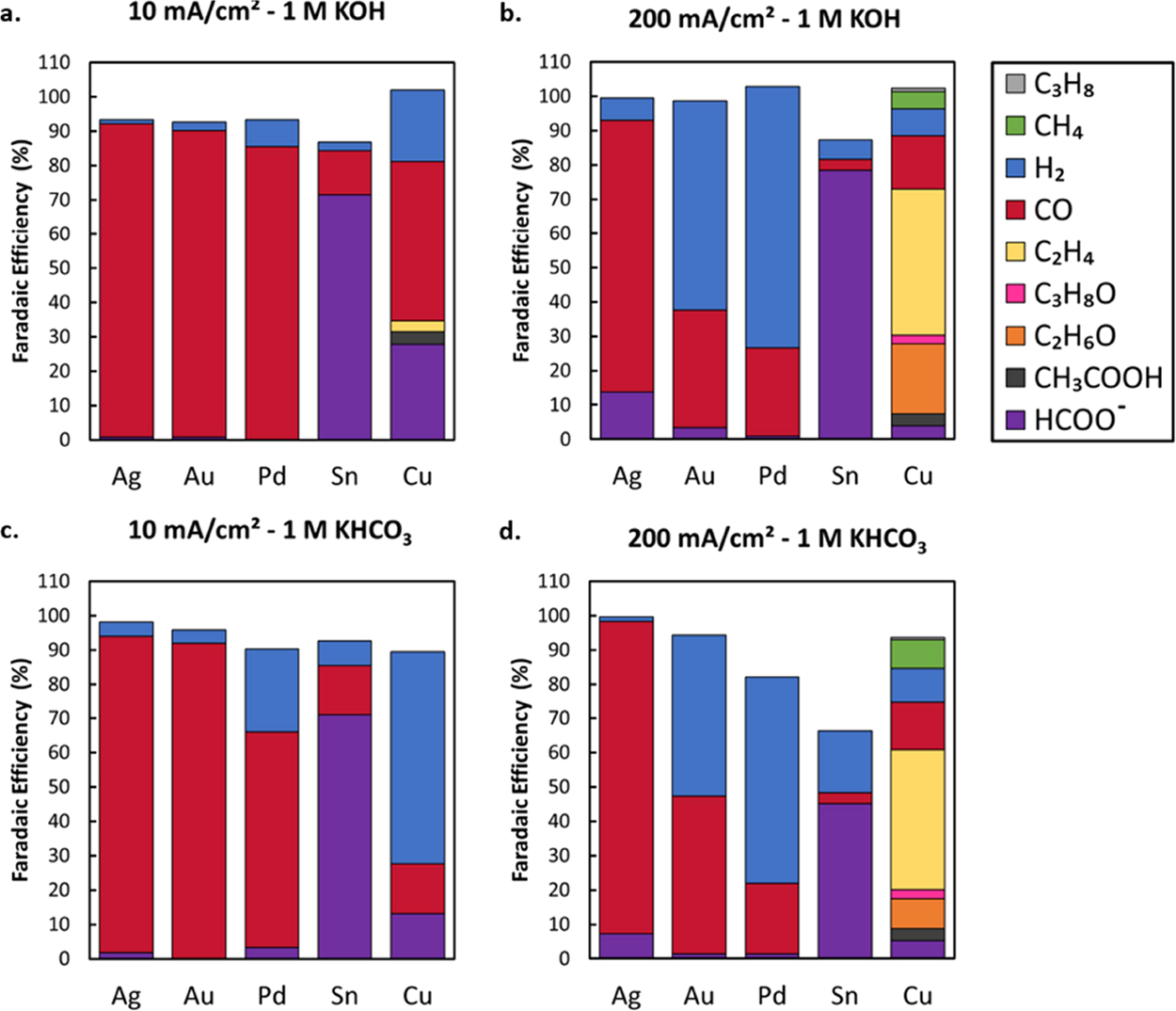
Selectivity comparison of transition metal catalysts in 1 M KOH at 10 mA cm^–2^ (a) and 200 mA cm^–2^ (b) and in 1 M KHCO_3_ at 10 mA cm^–2^ (c) and 200 mA cm^–2^ (d).

To this end, here we briefly provide a direct side-by-side comparison of the selectivity at low (10 mA cm^–2^) and higher (200 mA cm^–2^) current density as a reflection to the baseline work previously performed in an H-cell.^1^ As shown in Figure 8, we compare these current densities in 1 M KOH and 1 M KHCO_3_. The differences between the two electrolytes that are prevalent at lower current densities (Figure 8a,c) are much less impactful when going to 200 mA cm^–2^ (Figure 8b,d). The elevated rate of formed OH^–^ and consumed CO_2_ gradually closes the gap between both starting conditions. As a result, the product distribution of the catalyst homogenizes as its activity is increased, regardless of the electrolyte. We can also more clearly see that some metals match their product selectivity at higher current densities consistently with little variation, while others start favoring the HER or an alternative carbon product.

Importantly, comparing the 10 mA cm^–2^ flowcell results against the 5 mA cm^–2^ H-cell benchmark for CO_2_ reduction shows mostly similarities in applied potential and product selectivity, highlighting that the reaction rate is a more prominent performance indicator at lower current densities than the choice between H-cell and flow cells. This is likely because the reaction environment remains similar, and the difference in available surface area is less likely to be limiting.

## Conclusions

The main focus of this work is to provide a comparison of elemental catalysts by creating a controlled system and identifying the effect of current density on activity, selectivity, and stability while moving from the H-cell regime (10 mA cm^–2^) up to the mass transport capabilities of the GDE regime (200 mA cm^–2^). Emphasis was placed on comparability of metals by producing 100 nm thick samples, and performing electrochemical and material characterization, and assessing collected data according to a detailed protocol. Although such a protocol allows for correlating catalysts without bias, a downside of this approach is that only a singular experimental configuration is screened. None-the-less we have strived to perform this analysis in a well described and controlled testing environment for the confirmation and reproducibility of new and existing research within the field. Changes in performance due to varying catalysts, configurations, and operating conditions are then grounded by a common point of comparison.

## Supporting Information Available

Protocols, sample production, material characterization, performance characterization, characterization data, EIS data, chronopotentiometry data, time-dependent F.E. data, SEM images, HR-SEM images, XPS results, AFM images, and PTFE cell design (PDF)
