# Supplementary material for: Characterizing CO2 Reduction Catalysts on Gas Diffusion Electrodes: Comparing Activity, Selectivity, and Stability of Transition Metal Catalysts
Source: ACS Appl Energy Mater. 2022 May 3;5(5):5983–94. doi: 10.1021/acsaem.2c00160 (PMC9131424; doi:10.1021/acsaem.2c00160)
Supplement: Supplementary file 1 — ae2c00160_si_001.pdf [file ae2c00160_si_001.pdf]

Supporting Information

Characterizing CO_2 reduction catalysts on gas diffusion electrodes: Comparing activity, selectivity and stability of transition metal catalysts

Mark Sassenburg[1], Reinier de Rooij[1], Nathan Taylor Nesbitt[1], Recep Kas[1,2], Sanjana Chandrashekar[1], Nienke J. Firet[1], Kailun Yang[1], Kai Lui[1], Marijn A. Blommaert[1], Martin Kolen[1], Davide Ripepi[1], Wilson A. Smith[1,2], Thomas Burdyny[1]*

*Corresponding author: t.e.burdyny@tudelft.nl

[1]Materials for Energy Conversion and Storage (MECS), Department of Chemical Engineering, Delft University of Technology, 2629 ZH Delft, The Netherlands

[2]Department of Chemical and Biological Engineering and Renewable and Sustainable Energy Institute (RASEI), University of Colorado Boulder, Boulder, Colorado 80303, United States

SI A. Protocols

In order to obtain comparable results amongst both different materials and researchers it is necessary to streamline our methodologies (production, characterization and performance) for performing all planned experiments. It is of utmost importance that our individual approaches to each experiment are as similar as possible. To equally treat all samples our researchers specialized in certain characterization methods. Building of the cell for performance experiments was explained by one person to ensure practical details (e.g. ref. electrode spacing, cathode/anode fixation and cell tilt) to be uniform as well. This protocol will give the main guidelines to obtain comparable results for each technique and to allow reproduction of results.

Sample production

Samples were produced by means of **magnetron sputtering deposition**. To compare the parameters of different materials it is best to have similar thickness and structural morphology. It is assumed uniform, comparable structures are obtained by using continuous rotation, **20 sccm Ar** at **3 µbar** and similar deposition rates. **DC power of 50 W** was used for Au, Ag, Cu and Pd. More volatile Sn was produced at 20 W. To obtain the correct thickness the deposition rate will first be calculated by measuring the depth profile of a glass piece after 10 min sputtering. After determining the deposition rate for each material, the deposition time to make 100 nm (calculated on glass) will be used to make the GDE samples. It is assumed the error in glass-to-GDE transferability is similar between different materials. See Profilometry for more details about the thickness determination. For the sputtering deposition we will make use of the AJA2 as shown in fig. S1 on the left-end side of the AJA system.

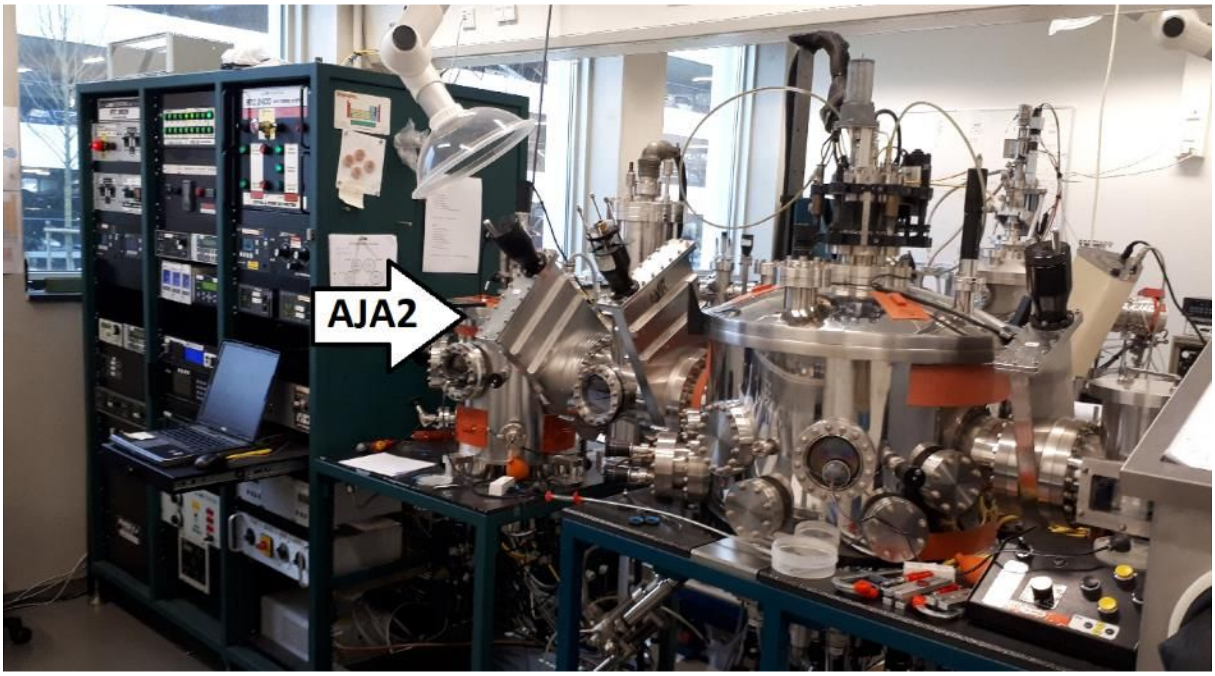

Figure S1. AJA, magnetron sputter. For our materials we will make use of the leftmost chamber (AJA2).

Material Characterization

Most material characterization techniques can be performed by one person, which makes executing the experiments and producing comparable results much easier. Nevertheless everyone should understand how and why we perform each technique and discuss the results of each method.

(High Resolution) Scanning Electron Microscopy – (HR)SEM

SEM (fig. S2) and **HR-SEM** are used to obtain information about **surface morphology** and with that track whether its structure changes after 1h operation. Found abnormalities like clusters, deformation, crystals or exposed MPL could indicate poor stability. To be able to compare materials (HR-)SEM images are used to show similar morphologies across different metals as a result of sputtering. The HR-SEM is located in a cleanroom. Images are made at the following magnifications:

-SEM (fig. S2): **x50** **x100** **x500** **x1000** **x5000**

-HR-SEM (cleanroom): **x50.000** **x100.000** **x150.000**

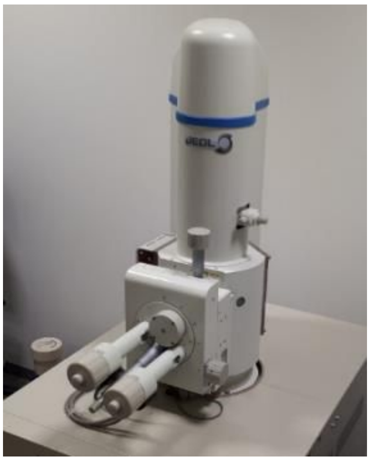

Figure S2. JEOL, JSM-6010LA Scanning electron microscope.

Atomic Force Microscopy – AFM

AFM (fig. S3) is another surface probing technique in addition to the SEM. This technique will give information on **surface topography and phase separation**. For AFM we make use of TESPA-v2 probes. Height and phase images of the catalyst surface at a magnification of **500 x 500 nm** and **1000 x 1000 nm** are taken**.** Some parameters of the AFM can be sample specific and need to be optimized during operation.

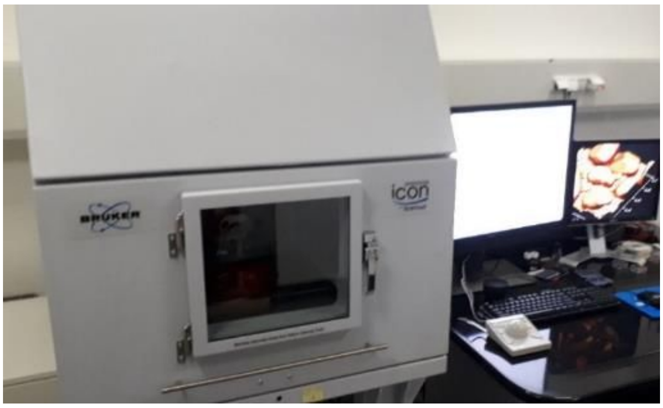

Figure S3. Bruker, Atomic Force Microscope with Dimension Icon ScanAsyst.

Profilometry

Profilometry (fig. S4) is a simple technique that measures the **thickness** of the samples by comparing the height to a reference point. The coarseness of the microporous layer is ±1 µm and the sputtered thickness we aim for is **100 nm**. Because of this coarseness, profilometry will be performed on a piece of Menzel glass, added to each sputtering procedure. By partially Scotch-taping the glass surface and peeling the tape away after sputtering, an abrupt interface between sputtered and non-sputtered glass is created. This

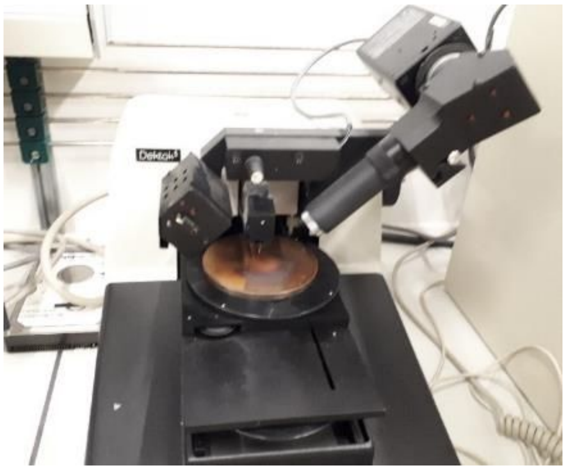
Figure S4. INRF, Dektak 3 profilometer.

interface is easily measured by profilometry, giving the thickness with a ±5 nm error. Although the porosity of catalysts on GDEs is higher than on glass, which results in a thicker layer on GDE, it is assumed that the translation of catalyst layer thickness between glass and GDE is constant over the range of materials. In the end the importance of this characterization is not the exact thickness, but to ensure similar thicknesses are present amongst all samples to obtain comparable mass transport properties.

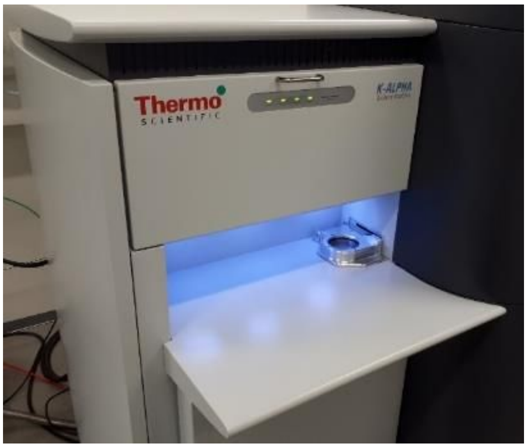

Figure S5. ThermoFisher Scientific, K-Alpha XPS.

X-ray Photoelectron Spectroscopy – XPS

XPS (fig. S5) gives clear information about the **purity and oxidation state** of the samples. This way we can exclude or take into account the effects of impurities and oxidation of the catalyst. For each catalyst a survey scan is performed first. Elements of interest are scanned separately to obtain more accurate data. A C 1s scan shows the presence of substrate carbon (and some omnipresent surface carbon). This peak will hint at if the substrate became more or less exposed after operation. Very close to the C 1s peak is the K 2p peak used to identify potassium, which can deposit from the electrolyte on the surface during operation. A respective metal scan (e.g. Au 4f for Au) is used to see change in the catalysts abundance and oxidation. Finally, an O 1s scan is used to measure the degree of oxidation.

Sample handling and rinsing

While producing, installing and ex-situ measuring GDE samples it is important to avoid any direct contact with the MPL/catalyst layer. Due to the powder like structure of the Sigracet 38 BC MPL this layer gives off its catalyst and carbon black easily. In such a case a new sample needed to be deployed, since this could have affected the homogeneity, loading and pore size distribution. Therefore it is important to have proper handling strategies. During the experiments samples were only touched on the sturdy GDL backside or along the edges of the catalyst side using tweezers (outside of the active area). After placing a sample between two gaskets a mask was placed over to prevent any contaminations. During ex-situ experiments this care was also taken and it was made sure only the active area was studied.

During the reaction with the potassium containing electrolytes it is likely that at the cathode secondary reactions with the potassium ions in solution would occur. In order to prevent post-reaction salt-crusting or residual oxidations a rigorous rinsing protocol was deployed. After each experiment the catholyte and anolyte streams were switched with a 250 mL DI water solution that was continuously recirculated through the cell for approx. 5 minutes to remove any residual $KHCO_3$/KOH electrolyte species. Afterwards the cell was opened and the sample was taken out with tweezers and sprayed again with bottled DI water for 30 seconds and subsequently dried using an abundant nitrogen stream. After this the samples were stored until ex-situ characterization was performed.

Performance Characterization

A major component of this comparison is the reproducibility of the experimental procedure. This section documents the details of the setup.

Cell construction and parameters

The experimental setup and the internal electrochemical cell will be built according to fig. S6 and S7:

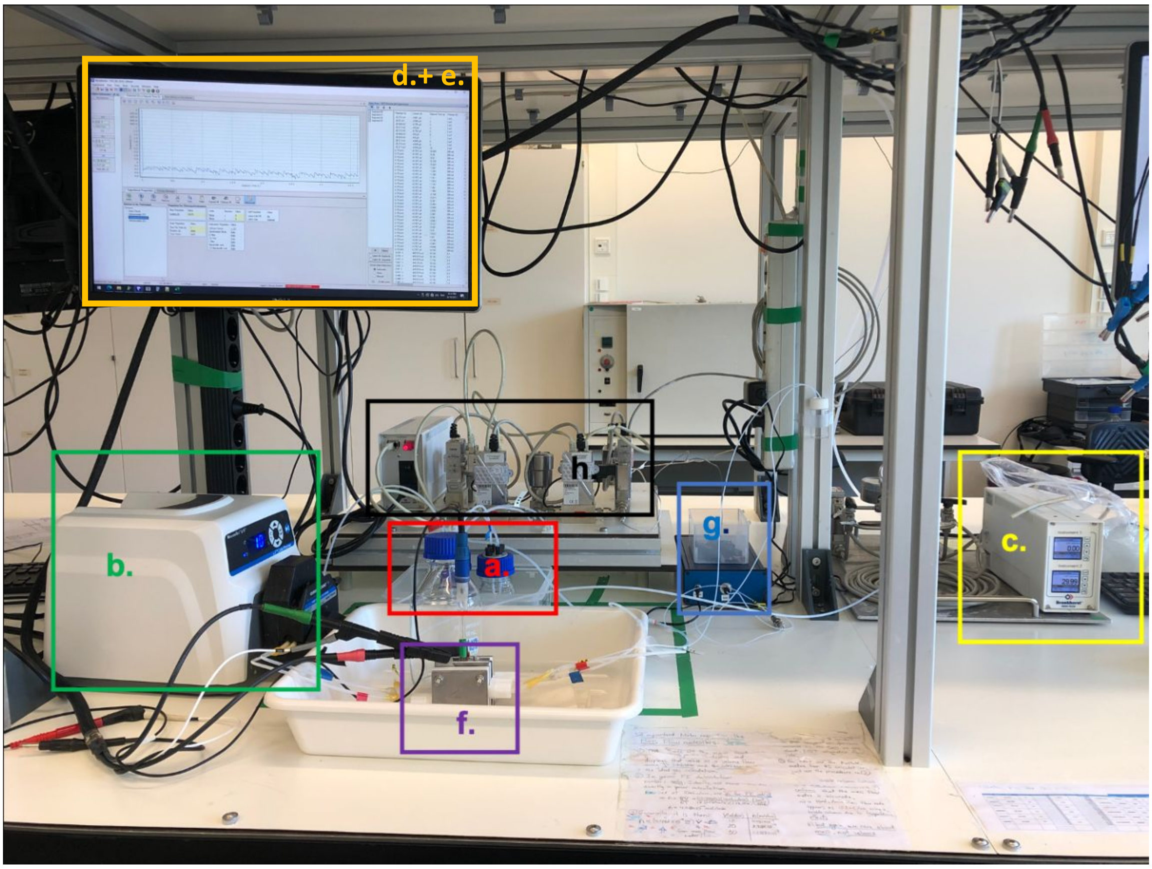

Figure S6. Practical experimental setup. a. External electrolyte compartments. b. Peristaltic pump (10 mL min^{-1}). c. MFC for CO_2 (30 sccm). d.+ e. Potentiostat, BPR and GC control. f. PTFE flowcell. g. Liquid trap. h. Back pressure regulator.

The external setup consists of 2 external electrolyte compartments (80 mL electrolyte each) from which the pump (Cole-Parmer Masterflex L/S, ϕ_{pump} = 10 mL min^{-1}) transported the anolyte and catholyte to the lower cell inlets. The corresponding upper outlets go back to the electrolyte compartments from the outlets. The cell is tilted slightly to aid in the transport of anode formed oxygen out from the top of the anolyte compartment, hereby reducing potential fluctuations. The CO_2 MFC (Bronkhorst EL-flow, ϕ_{MFC} = 30 sccm) is connected to the gas inlet of the cell and the outlet is connected directly to the GC once measurements are taken. During circulation of both liquid and gas an overpressure of 80-100 mbar is witnessed on the MFC pressure gauge. GC injections close the gas pathway temporarily (2-3 seconds) and cause overpressure to shortly spike to ~200 mbar, leading to minor gas crossover into the catholyte and subsequently leaving through the external compartment. The loss of gas has a minor effect on the gaseous product collection, but since this only occurs after every injection the system has a 4 minute time window to equilibrate before the following injection takes place.

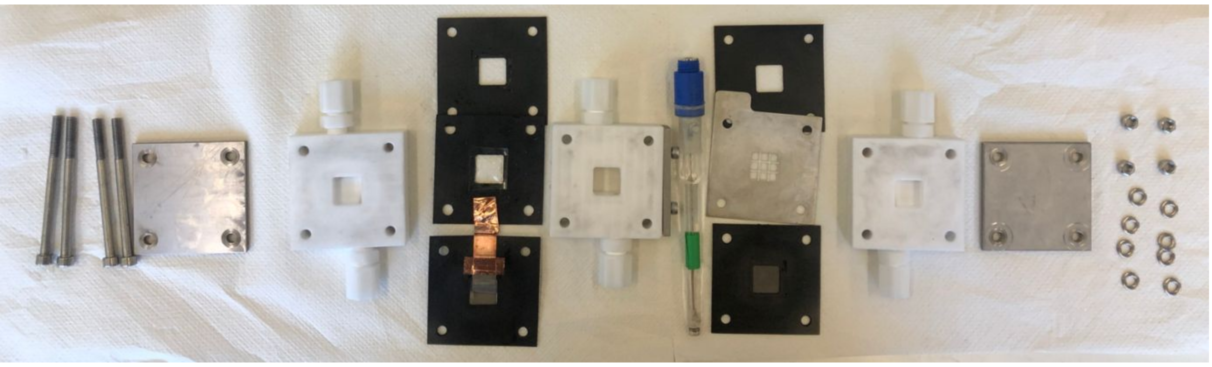

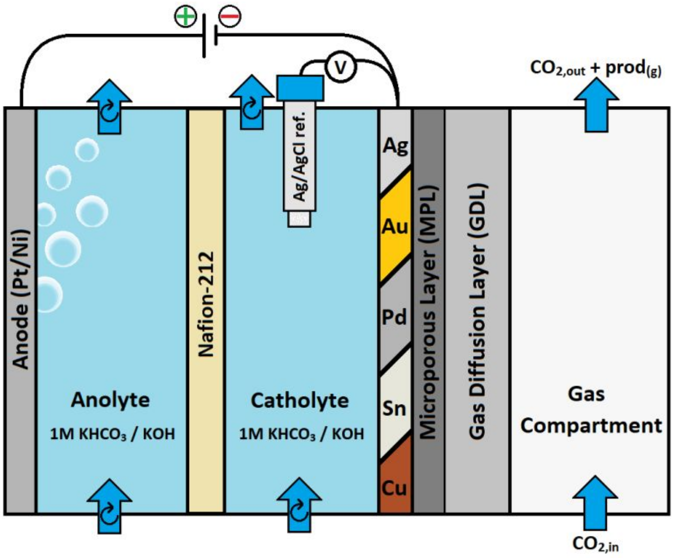

Figure S7. Exploded view of the 3 compartment cell (top) and schematic drawing of the cell interior (right).

From left to right: Anode (Ni mesh in 1 M KOH/Pt wire in 1 M $KHCO_3$) inside a recirculating anolyte compartment. Nafion-212 cation exchange membrane. Recirculating catholyte compartment with reference electrode. GDE with respective cathode catalyst layer on the microporous layer MPL facing the catholyte. CO_2 gas compartment. Between the anode and cathode the counter and working electrode are connected respectively applying a negative current. Between the working electrode and the Ag/AgCl reference potential is measured.

Between the anolyte and catholyte compartments the anode and membrane are positioned in a sandwich of gaskets. This sandwich consists of 5 parts: a gasket, the anode (bent away from the membrane and taped to the first gasket for electrical connection), a second gasket, the Nafion-212 cation exchange membrane and a third gasket. The sputtered GDE sample is also squished between two gaskets alongside a current collector. A more detailed description can be found in a paper by Liu et al. on assembly and operation of GDE cells [1].

Table S1. Setup conditions for alkaline and neutral experiments.

**Electrolyte type**	**Alkaline**	**Neutral**
**Starting pH**	13.8 – 14.0	7.8 - 8.0
**Anolyte**	1M KOH *(>85% pellets, Sigma Aldrich)*	1M $KHCO_3$ *(ACS reag. 99.7%, Sigma Aldrich)*
**Catholyte**	1M KOH *(>85% pellets, Sigma Aldrich)*	1M $KHCO_3$ *(ACS reag. 99.7%, Sigma Aldrich)*
**OER Anode**	Ni mesh	Pt wire
**Membrane**	Nafion-212	Nafion-212
**Ref. electrode**	XR310 Radiometer Analytical	XR310 Radiometer Analytical

Potentiostat

All potentiostatic measurements are performed with the **ParStat 4000** (fig S8) or **ParStat MC.**

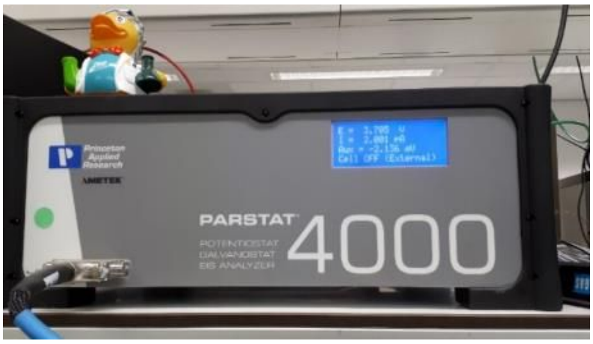

Figure S8. Ametek, Princeton Applied Research, ParStat 4000 Potentiostat/Galvanostat/EIS analyser.

pH is measured before and after experiments. By combining pH and EIS results, the measured potential can be converted to RHE:

$$E_{(RHE)} = E_{cath} + 0.0591\ pH + E^0_{Ag/AgCl} - iR_{drop}$$

Where E_{cath} is the measured negative potential between the reference and the cathode, $E^0_{Ag/AgCl}$ is the standard reference potential ($E^0_{Ag/AgCl}$ = 0.1976V vs RHE @ 25 °C) and iR_{drop} the negative current times measured resistance. Unfortunately it was not possible to accurately determine the iR drop through EIS at elevated current densities due to the long path of electrolyte between cathode and reference. More details on this can be found in SI B. Electrochemical impedance spectroscopy. Instead, all reported potentials are iR-uncorrected as described by the following formula:

$$E_{(uncorr.\ vs\ RHE)} = E_{cath} + 0.0591\ pH + E^0_{Ag/AgCl}$$

Chronopotentiometry experiments are the core part of this research. By keeping the current density (C.D.) constant and combining this with gas and liquid analysis we are able to determine the product selectivity at a certain production rate as well as the stability over time. The planar active surface area is 2.25 cm^2, so the potentiostat input are adapted as shown in brackets. Chronopotentiometry measurements are taken for 1 hour at the following currents:

- 10 mA/cm^2 (comparable C.D. to H-cell activity) [= - 22.5 mA]
- 50 mA/cm^2 [= - 112.5 mA]
- 100 mA/cm^2 [= - 225 mA]
- 200 mA/cm^2 [= - 450 mA]
- 300 mA/cm^2 [= - 675 mA]

After an experiment catholyte samples are taken and analyzed by HPLC for liquid products.

Gas Chromatography (GC)

GC (fig. S9) measurements are taken every 4 minutes from the gas phase outlet during all chronopotentiometry experiments to measure the concentration of gaseous products. Before each measurement 3 injections without applied potential are performed as a second check of the baseline after flushing the system. It is important to perform regular calibration checks to ensure measurements are accurate. The gas products CO, H_2 and C_2H_4 are calibrated at 3 levels (10, 100, 1000 ppm).

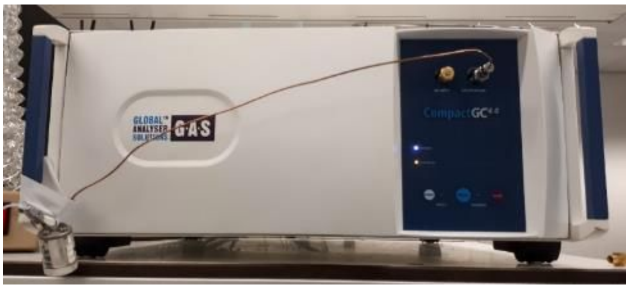

Figure S9. Global Analyser Solutions, CompactGC 4.0 gas chromatograph.

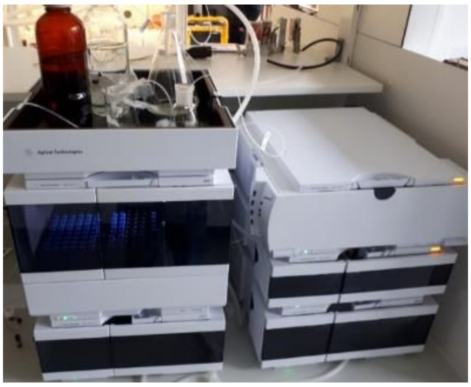

Figure S10. Agilent Technologies, 1260 Infinity II HPLC.

High Performance Liquid Chromatography (HPLC)

After each chronopotentiometry experiment a sample of catholyte will be taken to the Agilent Technologies 1260 Infinity II **HPLC** (fig. S10) equipped with VWD (dual wavelength: 210 nm and 280 nm) and RID (T = 40 °C) to measure the concentration of liquid carbon containing species on basis of their retention times in an Hi-Plex H column (T = 50 °C). Main products of interest here are formic acid, acetic acid, acetaldehyde, ethanol and propanol. 10 levels of calibration were performed (10-10000 ppm, $R^2 > 99.9$ %)

SI B. Characterization data

This section displays the results of the performed experiments. Data is collected via chronopotentiometry, electrochemical impedance spectroscopy (EIS), gas and liquid product analysis (GC/HPLC), (high resolution-) scanning electron microscopy (SEM & HR-SEM imaging), X-ray photoelectron spectroscopy (XPS) and atomic force microscopy (AFM).

Electrochemical impedance spectroscopy – EIS

Electrochemical impedance spectroscopy can be used to determine the resistance between the reference and working electrode and consecutively correct the measured potential for any resistive losses. During this series of experiments an EIS measurement was performed before and after each experiment. It was found that the configuration of our system had a significant drawback: Due to the relatively large distance between the reference electrode and the GDE cathode (~8 mm) the measured resistance was **3-4 Ω** for 1 M KOH and **7-8 Ω** for $KHCO_3$. This extremely large correction factor caused the iR corrections to lower the voltage significantly. To illustrate: 8 Ω * 450 mA = 3.6 V of ohmic drop. Furthermore, it was found that correcting potentials at 100/200 mA cm^{-2} with said resistance caused overcompensation. For instance, the corrected potential at 200 mA cm^{-2} in 1 M $KHCO_3$ was lower than the corrected potential at 50 mA cm^{-2}. In some cases the iR-drop even exceeded the applied potential, indicating that the resistance during operation at elevated currents was actually lower than we were able to measure during 'offline', no-current EIS.

Concluding, it was decided to not correct the measured potential for its resistive losses. This was done to avoid confusion about the measured potentials due to changing electrolyte conductivities throughout the length of the experiment, and to evade overcompensation of unrealistic resistances.

Chronopotentiometry

Below V_{cat}-t chronopotentiometry diagrams of all metal-electrolyte combinations are shown as measured against an Ag/AgCl reference electrode without corrections for iR-drop (fig. S11 – S15). Most of the high current experiments show diverging from the patterns. These values indicate the instabilities of the system at elevated activities, due to bubble formation in the electrolytes resulting in issues such as GDE flooding and gas crossover.

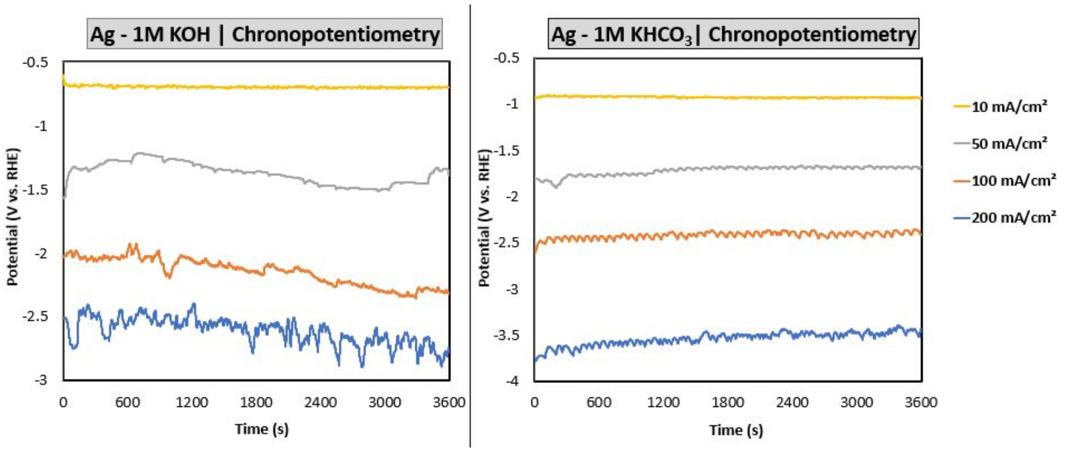

Figure S11. Chronopotentiometry measurements for Ag in 1 M KOH and $KHCO_3$.

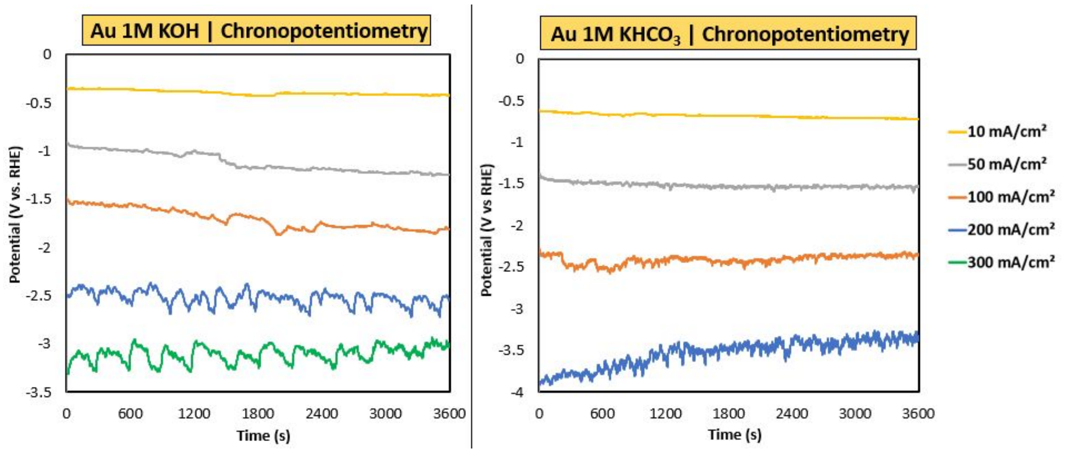

Figure S12. Chronopotentiometry measurements for Au in 1 M KOH and $KHCO_3$.

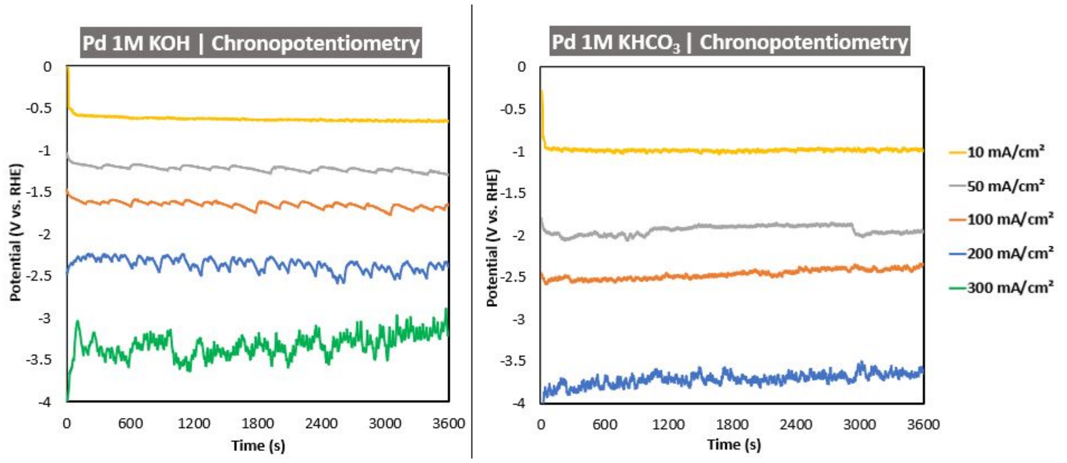

Figure S13. Chronopotentiometry measurements for Pd in 1 M KOH and $KHCO_3$.

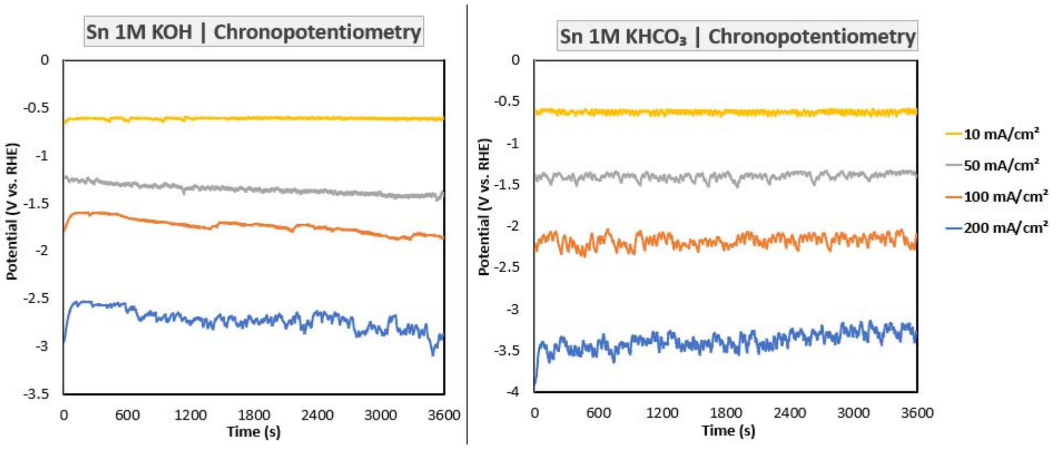

Figure S14. Chronopotentiometry measurements for Sn in 1 M KOH and $KHCO_3$.

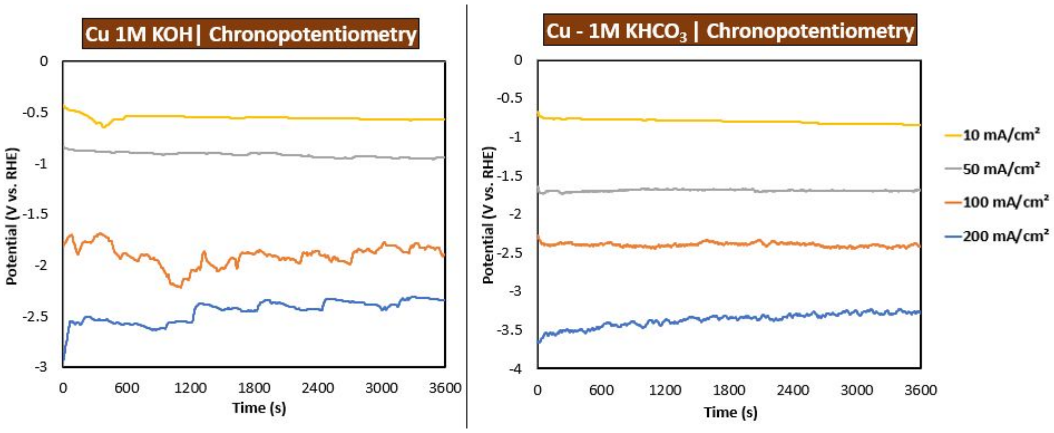

Figure S15. Chronopotentiometry measurements for Cu in 1 M KOH and $KHCO_3$.

Time-dependent Faradaic efficiencies - FE

During all experiments GC samples were taken every 4 minutes. The measured signal is converted into a concentration. Liquid analysis by HPLC is only performed when the experiment is completed after which the production is averaged over the duration. The following formula is used to calculate and plot the time-dependent Faradaic efficiencies (fig. S16 – S20). $\boldsymbol{FE_n}$ is the Faradaic efficiency of product n, $\boldsymbol{z_n}$ the number of electrons per formed molecule of product n, $\boldsymbol{F}$ the Faraday constant, $\boldsymbol{c_n}$ the concentration of n measured by the GC, $\boldsymbol{\varphi_{CO2}}$ the molar flowrate of CO_2 and $\boldsymbol{I_{tot}}$ the total current going through the system.

$$FE_n = \frac{z_n F (c_n * \varphi_{CO_2})}{I_{tot}}$$

The investigated gaseous products are H_2 (z = 2), CO (z = 2), CH_4 (z = 8) and C_2H_4 (z = 12) [mol_e/mol_n]. $\boldsymbol{F}$ = 96485 [C/mol_e], $\boldsymbol{c_n}$ [mol_n/mol_{CO2}], $\boldsymbol{\varphi_{CO2}}$ (=30 mL/min) = 4,46*10^{-5} [mol_{CO2}/s].

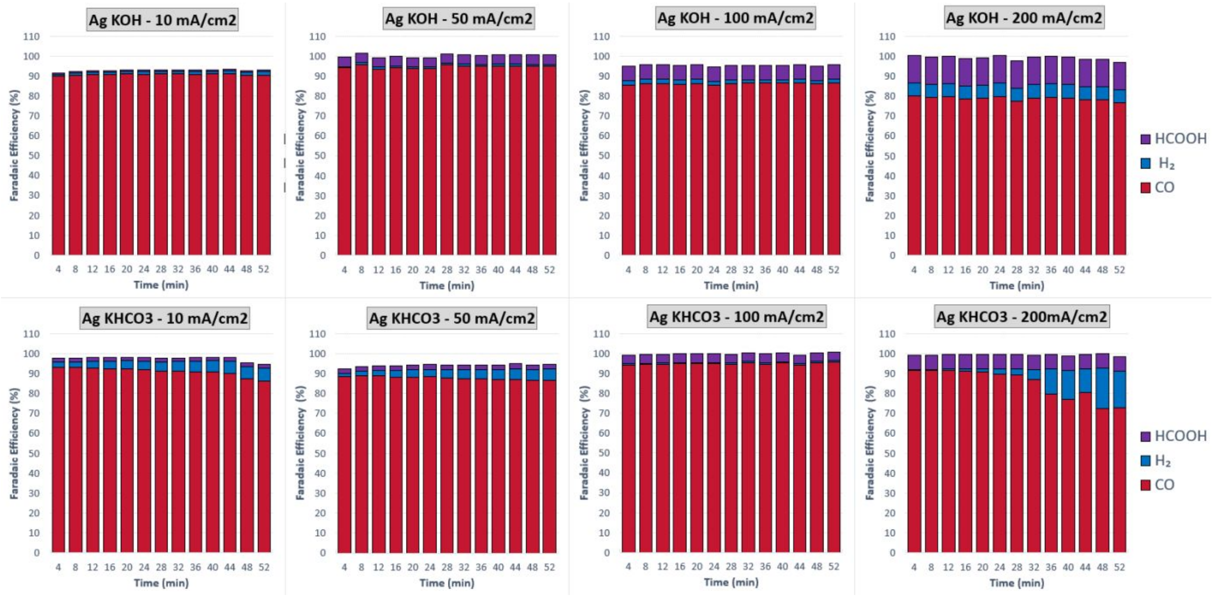

Figure S16. Time-dependent FE of products for a 100 nm Ag GDE.

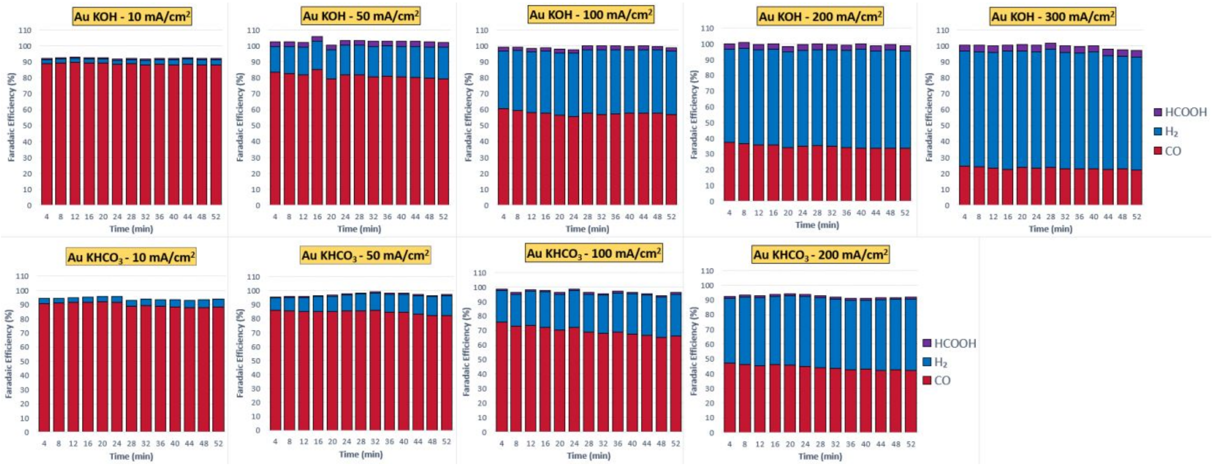

Figure S17. Time-dependent FE of products for a 100 nm Au GDE.

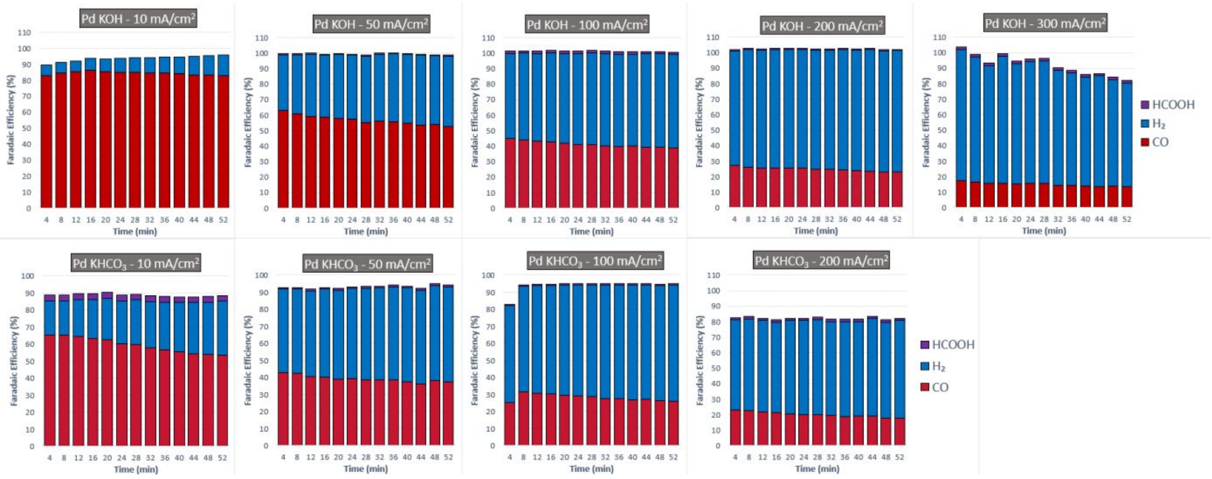

Figure S18. Time-dependent FE of products for a 100 nm Pd GDE.

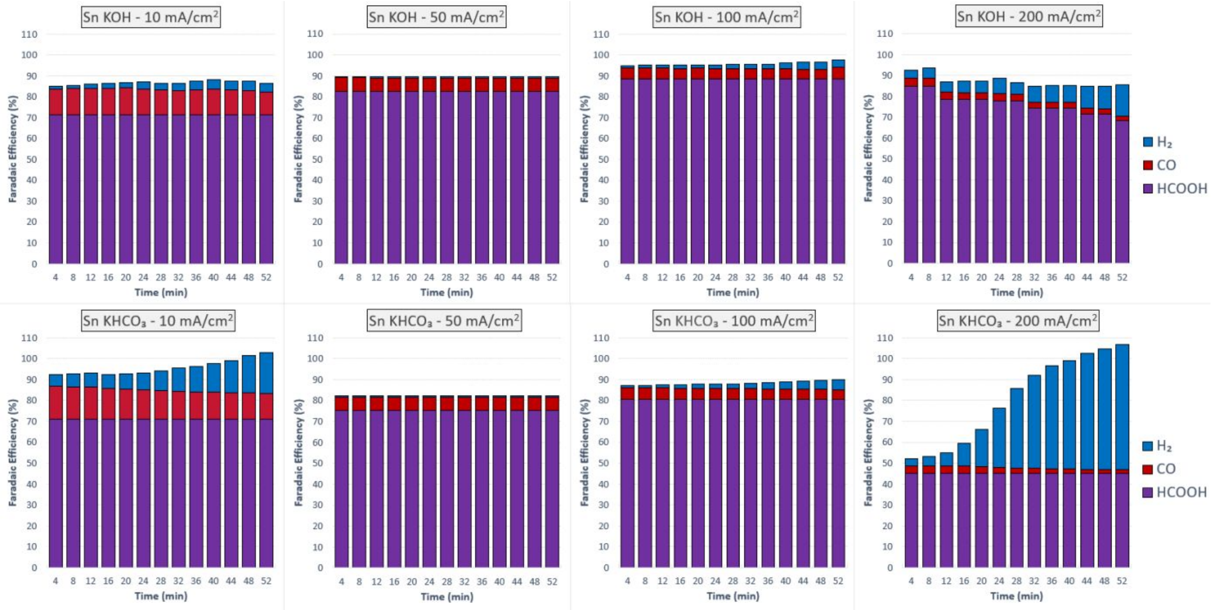

Figure S19. Time-dependent FE of products for a 100 nm Sn GDE.

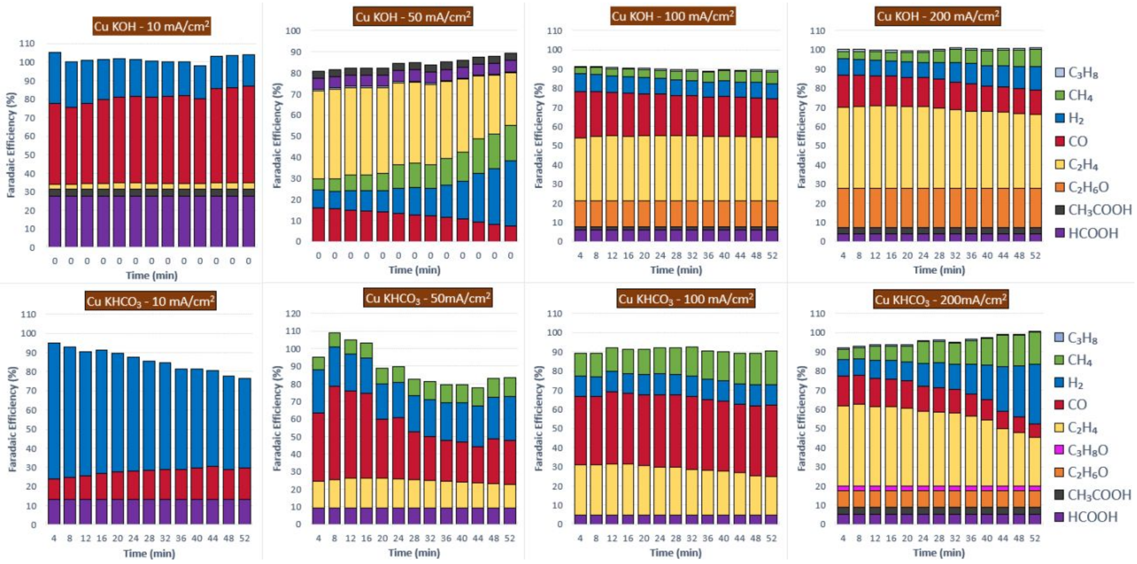

Figure S20. Time-dependent FE of products for a 100 nm Cu GDE.

Scanning electron microscopy - SEM

In figure S21 below HR-SEM images of the as-deposited catalysts are displayed. All catalysts show similar porosity and catalyst coverage on the GDE. Sn shows slightly increased agglomeration of the catalysts due to the more volatile nature of the metal *(Note: the deposition power of Sn was 20 W as compared to 50 W for the other metals.)*

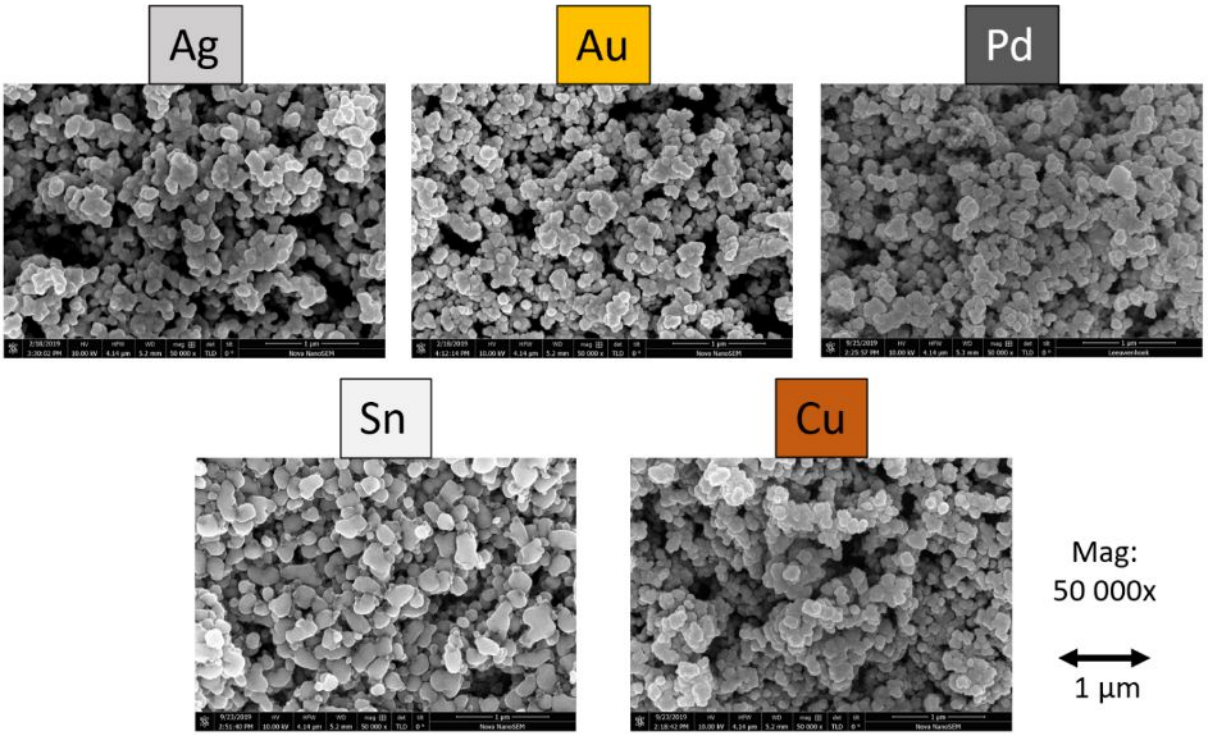

Figure S21. HR-SEM images of all 5 as-deposited catalysts. Throughout a similar porosity and particle size can be observed.

The remainder of this section contains scanning electron microscopy images taken from a bare Sigracet 38 BC GDL (fig. S22) as well as fresh and used samples (fig. S23 – S32). Initially 500 mA cm^{-2} (=1.125 A) experiments were also attempted, but due to the limiting compliance voltage of our potentiostat (ΔU_{max} = 12 V_{cell}) alongside the high resistance of the wide cell, it was not possible to achieve the requested current. Instead, the physical effects of 12 V total cell potential can be witnessed, at which current densities between 300-500 mA cm^{-2} were achieved. SEM images of these experiments do show interesting features of potassium depositions, catalyst reconstruction and a continuation of the earlier observed surface changes to a greater extent.

Magnifications and total dimensional image sizes

Magnification:<br>**x 50**<br>Size:<br>**~2.6 x 1.7 mm**	Magnification:<br>**x 100**<br>Size:<br>**~1.3 x 0.85 mm**	Magnification:<br>**x 500**<br>Size:<br>**~260 x 170 µm**	Magnification:<br>**x 1000**<br>Size:<br>**~130 x 85 µm**	Magnification:<br>**x 5000**<br>Size:<br>**~26 x 17 µm**

Bare gas diffusion layer (GDL)

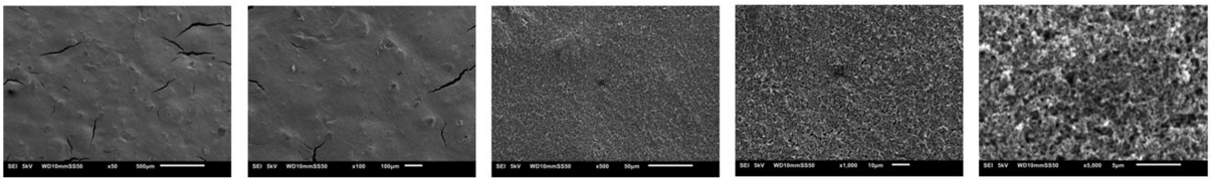

Figure S22. SEM images of a bare gas diffusion layer (Sigracet 38 BC)

Ag – KOH

100nm Ag - 10mA/cm^2 – 1M KOH

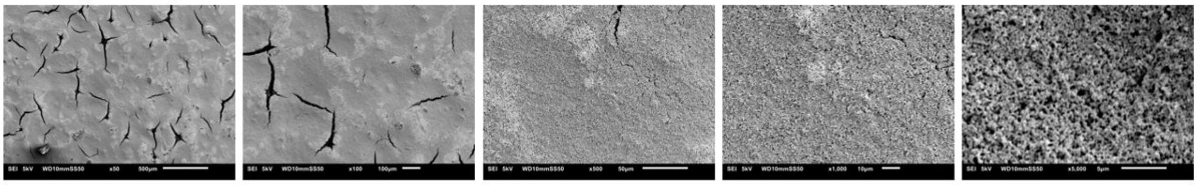

100nm Ag - 50mA/cm^2 – 1M KOH

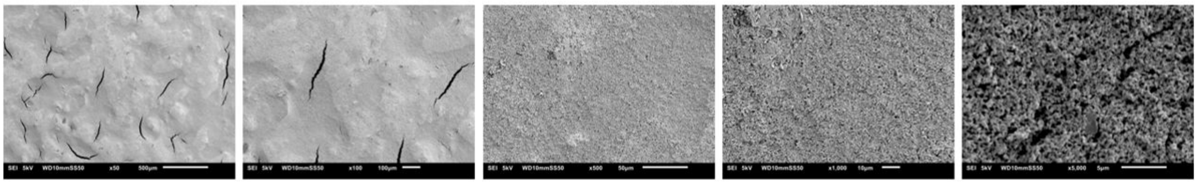

100nm Ag – 100mA/cm^2 – 1M KOH

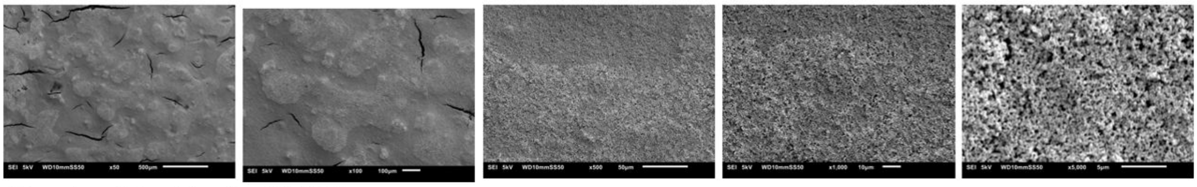

100nm Ag - 200mA/cm^2 – 1M KOH

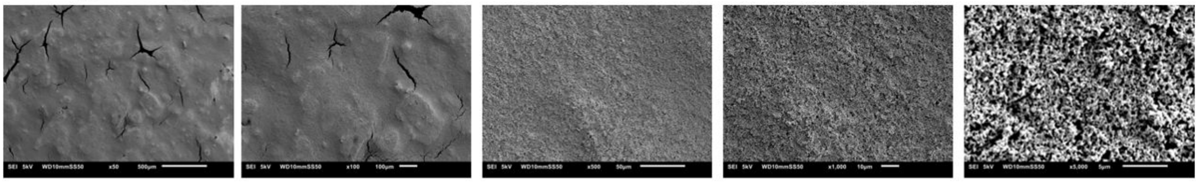

100nm Ag - 12 V_{cell} – 1M KOH

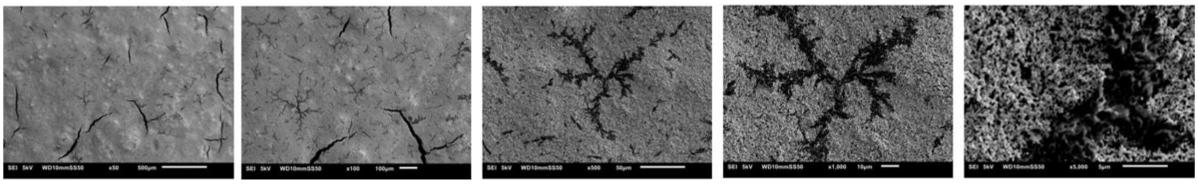

Figure S23. SEM images of 100 nm Ag on GDE after 1 hour reaction in 1M KOH at various current densities. Overall this system seems stable up to 200 mA cm^{-2}. At 500 mA cm^{-2} the surface displays branched coverage, which was identified to mainly contain increased amounts of potassium and oxygen. It is likely the high overpotentials initiated a growing deposition of potassium from the electrolyte.

x 50<br>~2.6 x 1.7 mm	x 100<br>~1.3 x 0.85 mm	x 500<br>~260 x 170 µm	x 1000<br>~130 x 85 µm	x 5000<br>~26 x 17 µm

Ag – $KHCO_3$

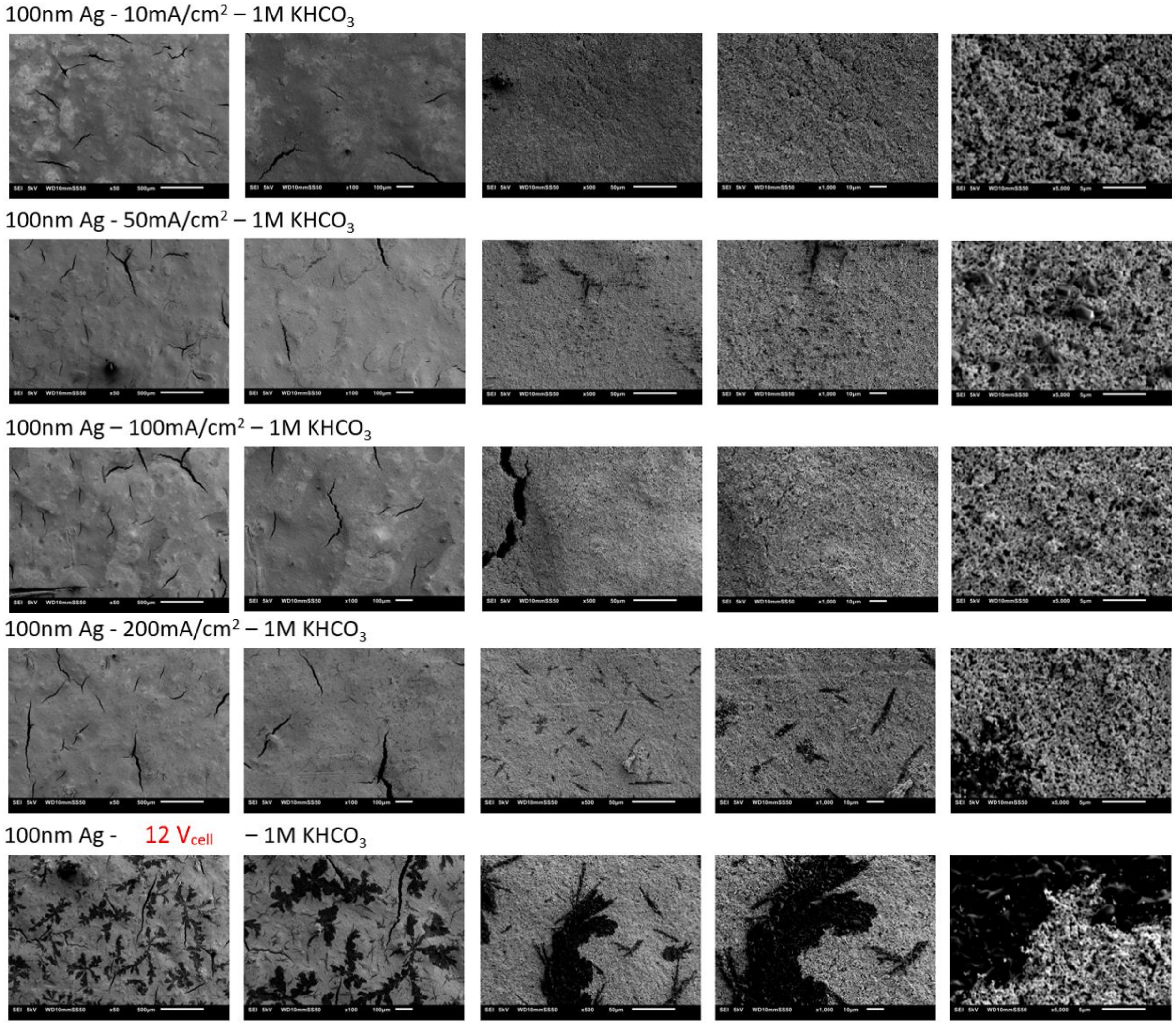

Figure S24. SEM images of 100 nm Ag on GDE after 1 hour reaction in 1M $KHCO_3$ at various current densities. At 50 and 100 mA cm^{-2} minor crystals are visible in the x5.000 magnification images. Even higher currents show exponential growth of these crystals. At 12 V the surface is largely covered by this black deposition, with features reaching up to 1-2 mm in length. Again potassium and oxygen were found in increased quantities in these areas.

Au – KOH

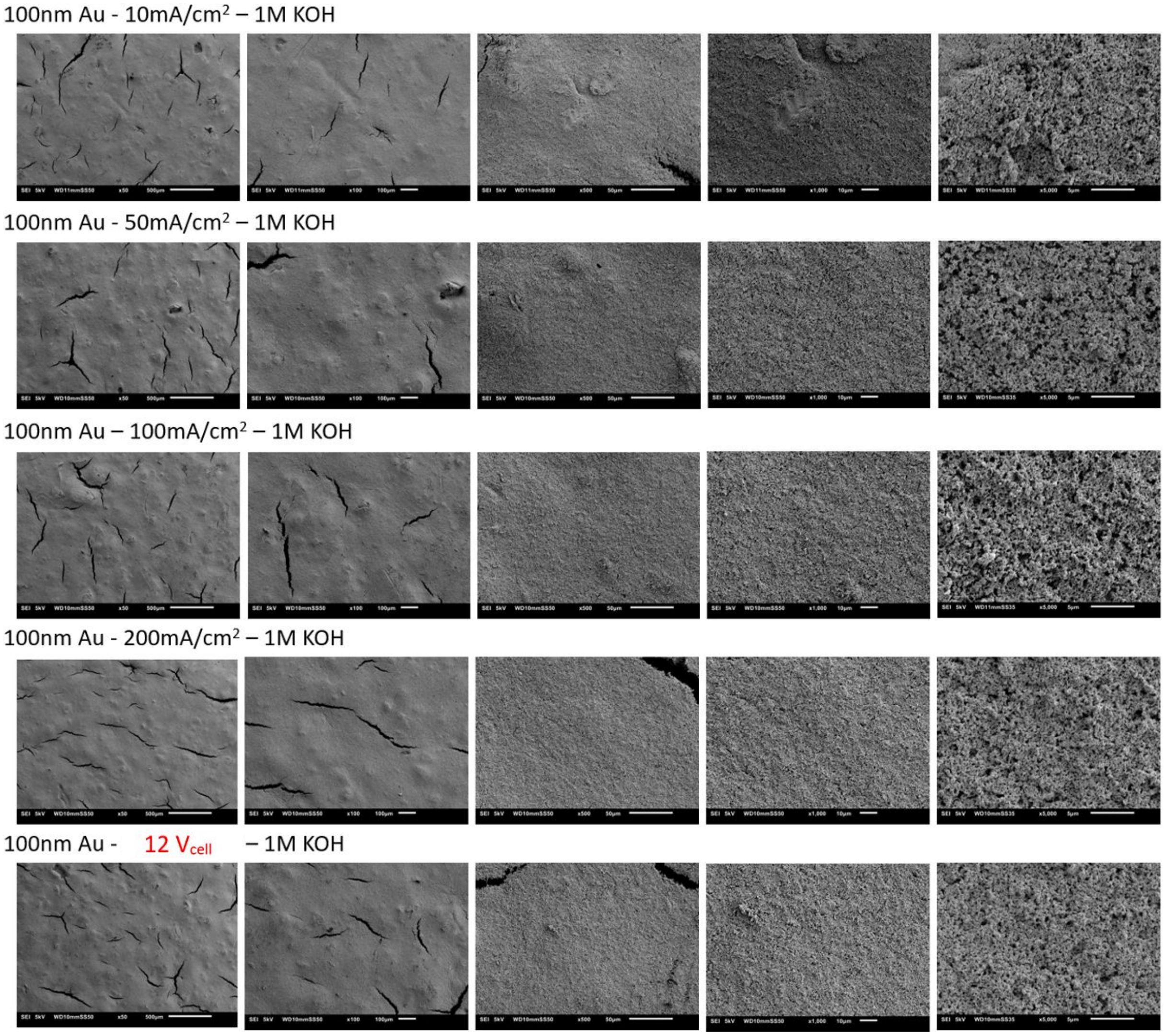

Figure S25. SEM images of 100 nm Au on GDE after 1 hour reaction in 1 M KOH at various current densities. Au is stable and no apparent changes across all 5 samples are found under these conditions.

x 50<br>~2.6 x 1.7 mm	x 100<br>~1.3 x 0.85 mm	x 500<br>~260 x 170 µm	x 1000<br>~130 x 85 µm	x 5000<br>~26 x 17 µm

Au – $KHCO_3$

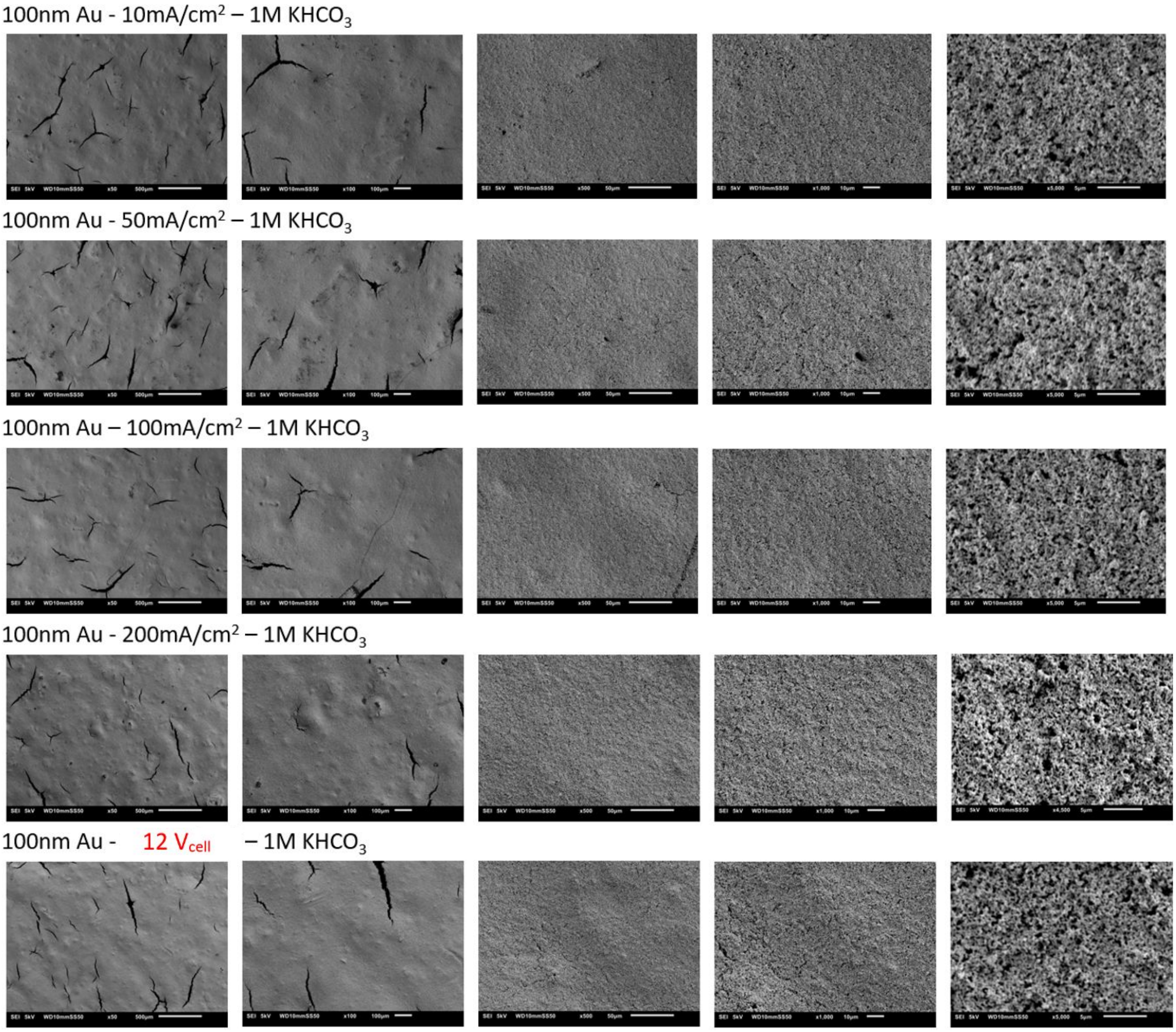

Figure S26. SEM images of 100 nm Au on GDE after 1 hour reaction in 1 M $KHCO_3$ at various current densities. Au is stable and no apparent changes across all 5 samples are found under these conditions.

x 50<br>~2.6 x 1.7 mm	x 100<br>~1.3 x 0.85 mm	x 500<br>~260 x 170 µm	x 1000<br>~130 x 85 µm	x 5000<br>~26 x 17 µm

Pd – KOH

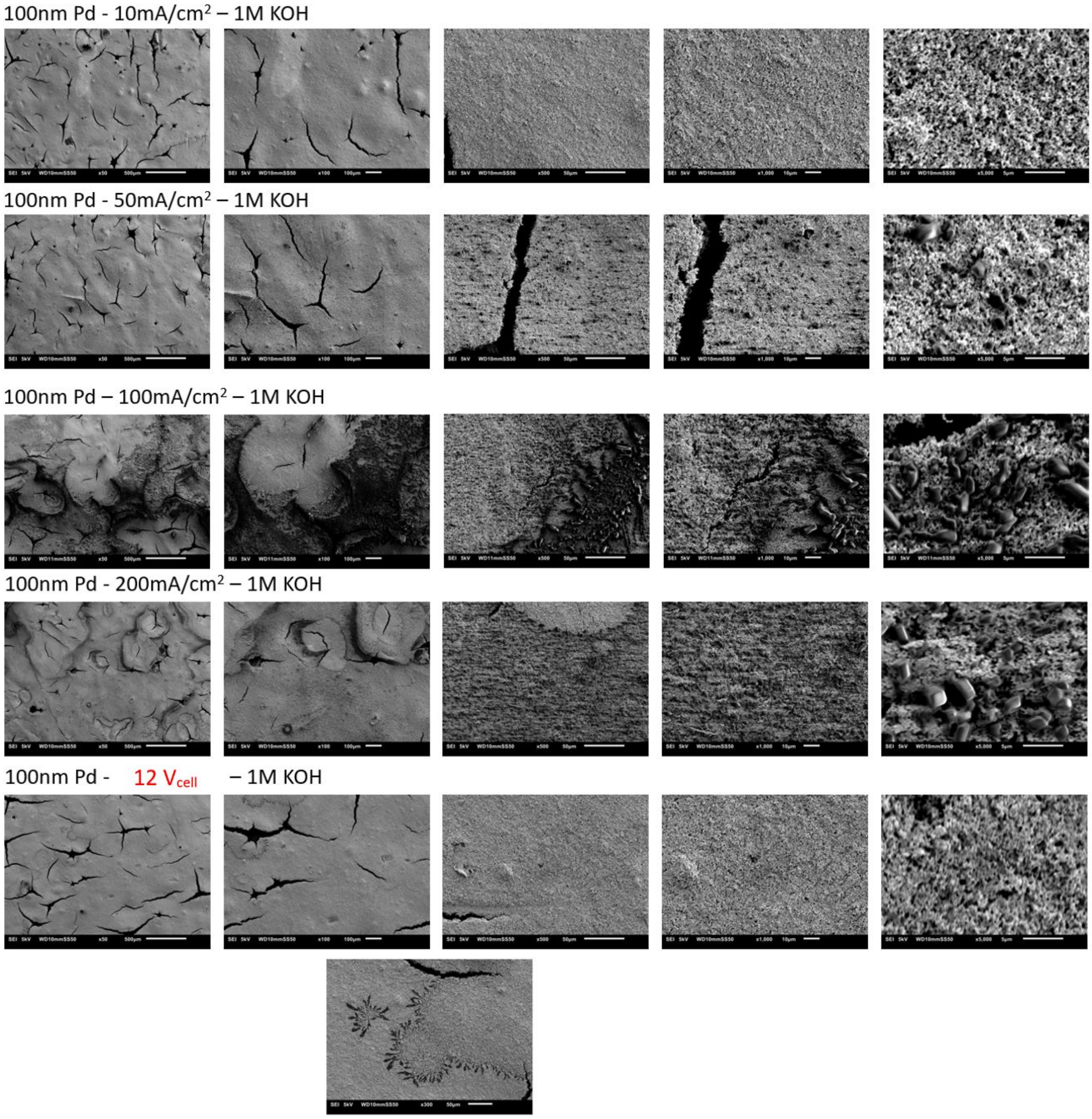

Figure S27. SEM images of 100 nm Pd on GDE after 1 hour reaction in 1 M KOH at various current densities. All current densities above the lowest (10 mA cm^{-2}) show surface instability and coverages to different extent. The surface seems to change the most at 100 mA cm^{-2}, after which higher currents and potentials have a lesser disrupting effect.

x 50<br>~2.6 x 1.7 mm	x 100<br>~1.3 x 0.85 mm	x 500<br>~260 x 170 µm	x 1000<br>~130 x 85 µm	x 5000<br>~26 x 17 µm

Pd – $KHCO_3$

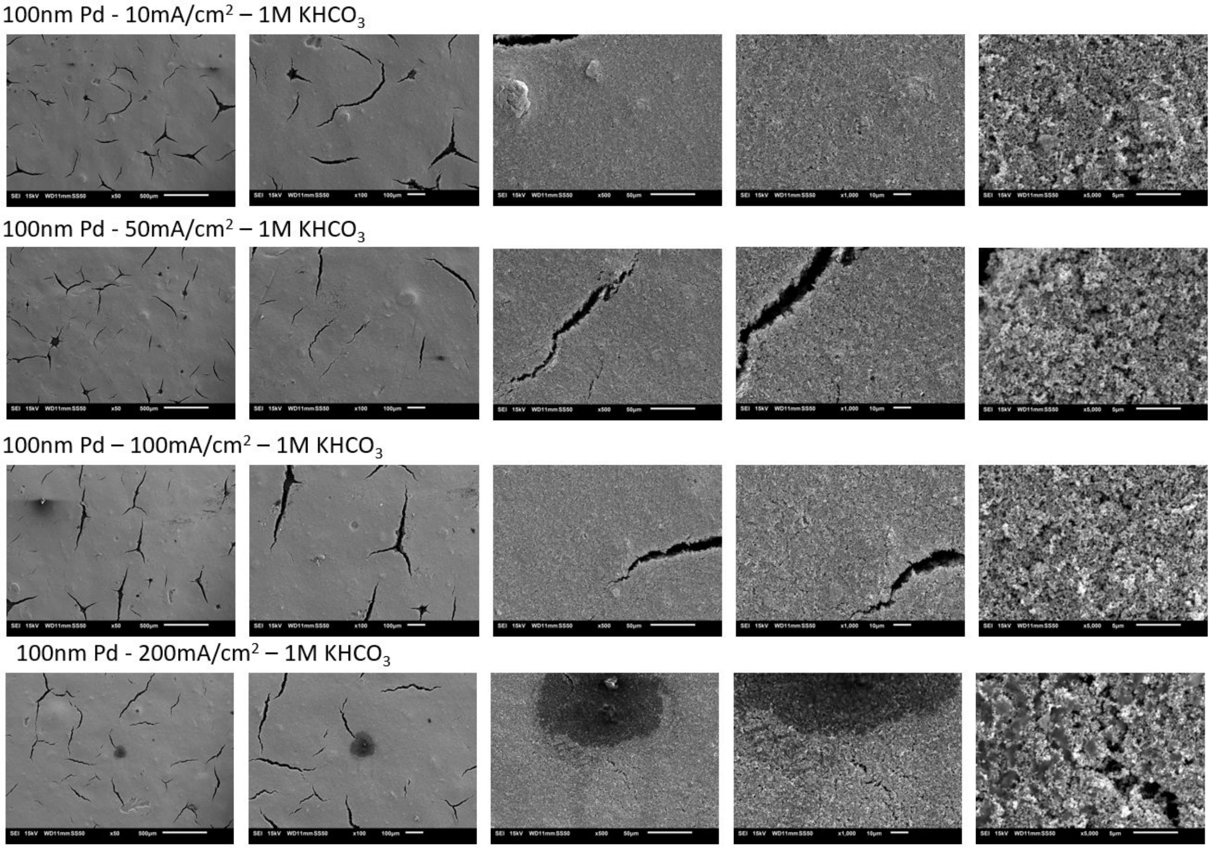

Figure S28. SEM images of 100 nm Pd on GDE after 1 hour reaction in 1 M $KHCO_3$ at various current densities. In comparison to the 1 M KOH case these samples show much better stability with a small fraction of the surface being altered.

x 50<br>~2.6 x 1.7 mm	x 100<br>~1.3 x 0.85 mm	x 500<br>~260 x 170 µm	x 1000<br>~130 x 85 µm	x 5000<br>~26 x 17 µm

Sn – KOH

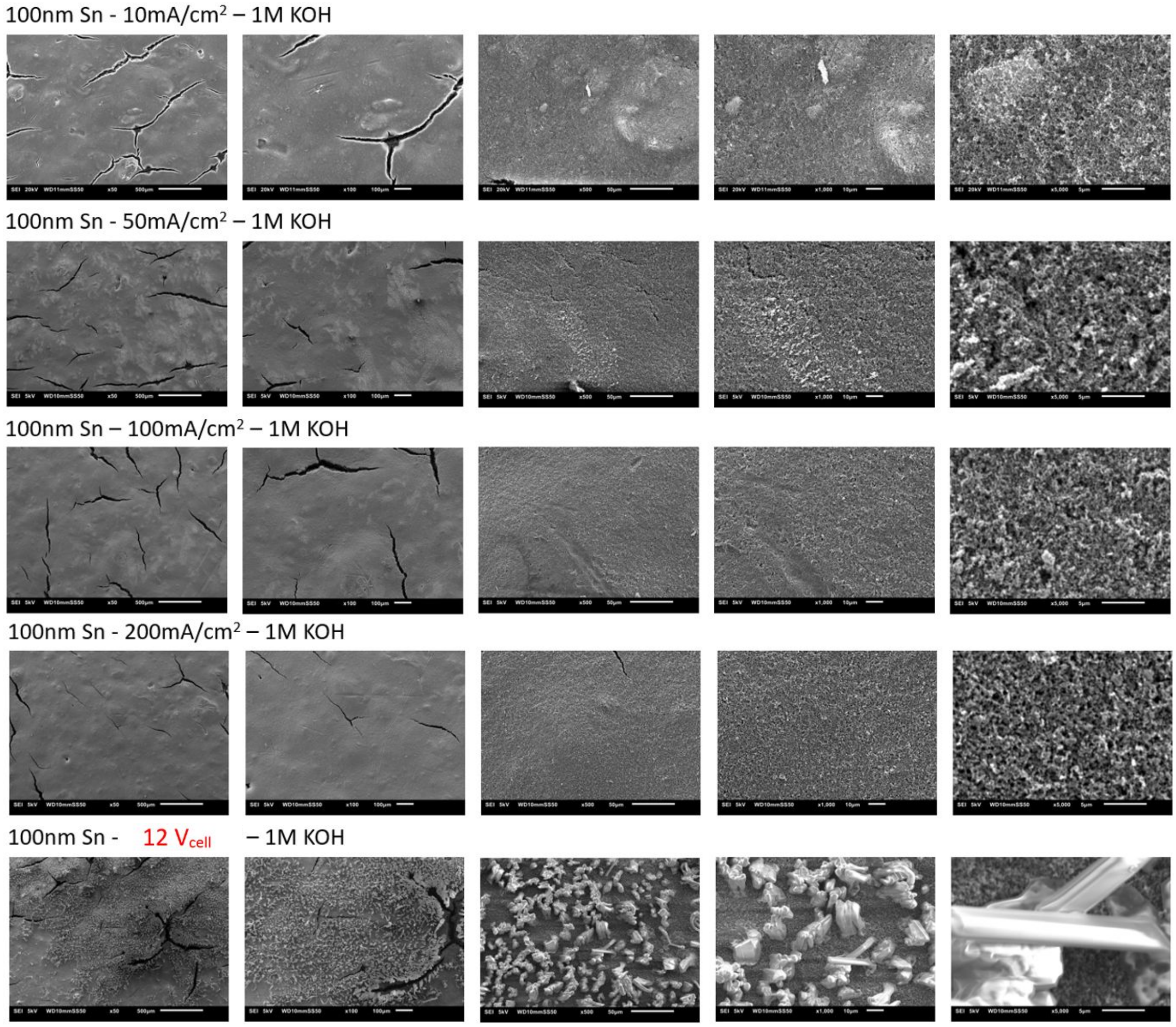

Figure S29. SEM images of 100 nm Sn on GDE after 1 hour reaction in 1 M KOH at various current densities. Overall the Sn samples seems rather stable at low and moderate activities. The x5000 image of the 200 mA cm^{-2} case showed 'thinner' GDE structures, indicating minor dissolution of the catalyst. Looking at the samples exposed to 12 V_{cell} a remarkable sight was obtained. The surface of the GDE was for a large part covered in potassium containing crystals.

x 50	x 100	x 500	x 1000	x 5000
~2.6 x 1.7 mm	~1.3 x 0.85 mm	~260 x 170 µm	~130 x 85 µm	~26 x 17 µm

Sn – KHCO$_3$

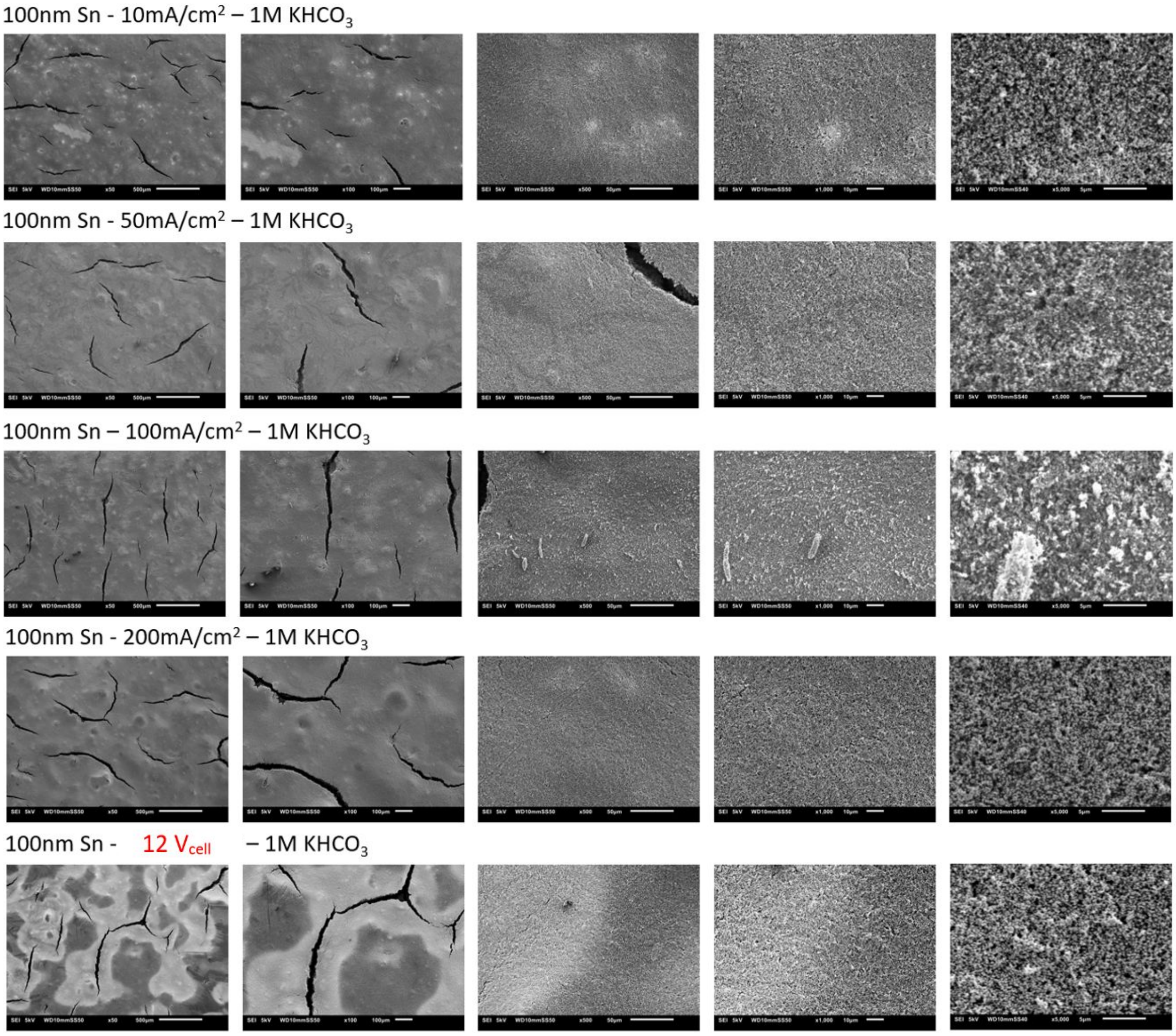

Figure S30. SEM images of 100 nm Sn on GDE after 1 hour reaction in 1 M KHCO$_3$ at various current densities. Whereas the KOH experiments showed significant instabilities induced through alkaline conditions, the less extreme environment of KHCO$_3$ only showed a change in color when increasing activity. No distinct differences of what the nature of the decoloring could be was found when looking into elemental mapping.

x 50<br>~2.6 x 1.7 mm	x 100<br>~1.3 x 0.85 mm	x 500<br>~260 x 170 µm	x 1000<br>~130 x 85 µm	x 5000<br>~26 x 17 µm

Cu – KOH

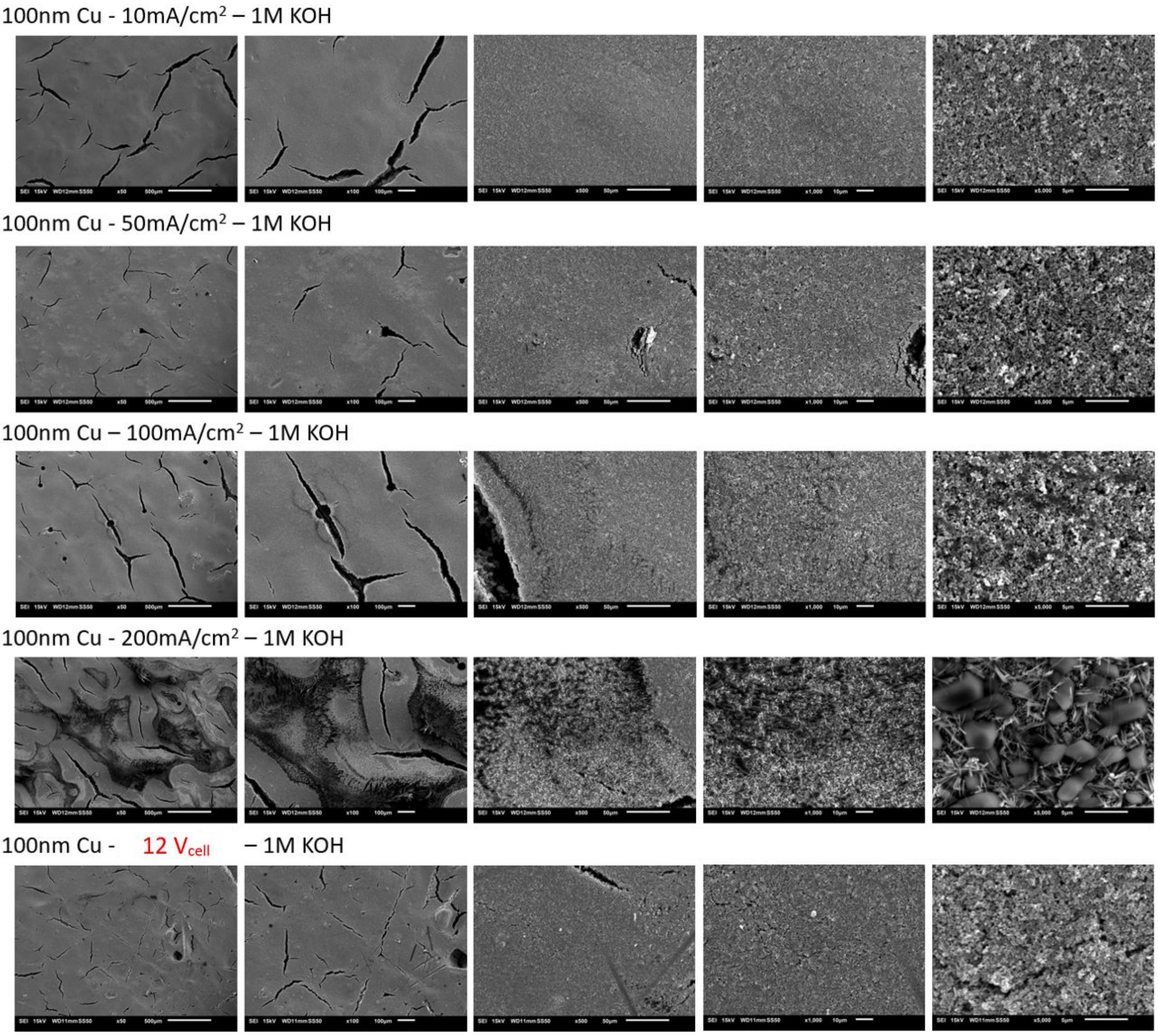

Figure S31. SEM images of 100 nm Cu on GDE after 1 hour reaction in 1 M KOH at various current densities. Minor restructuring is observed at 50, 100 and 500 mA cm^{-2}. The 200 mA cm^{-2} appears to supply the surface with a very specific environment that allows the growth of both facetted potassium-containing crystals as well as Cu nanoneedles.

x 50<br>~2.6 x 1.7 mm	x 100<br>~1.3 x 0.85 mm	x 500<br>~260 x 170 µm	x 1000<br>~130 x 85 µm	x 5000<br>~26 x 17 µm

Cu – $KHCO_3$

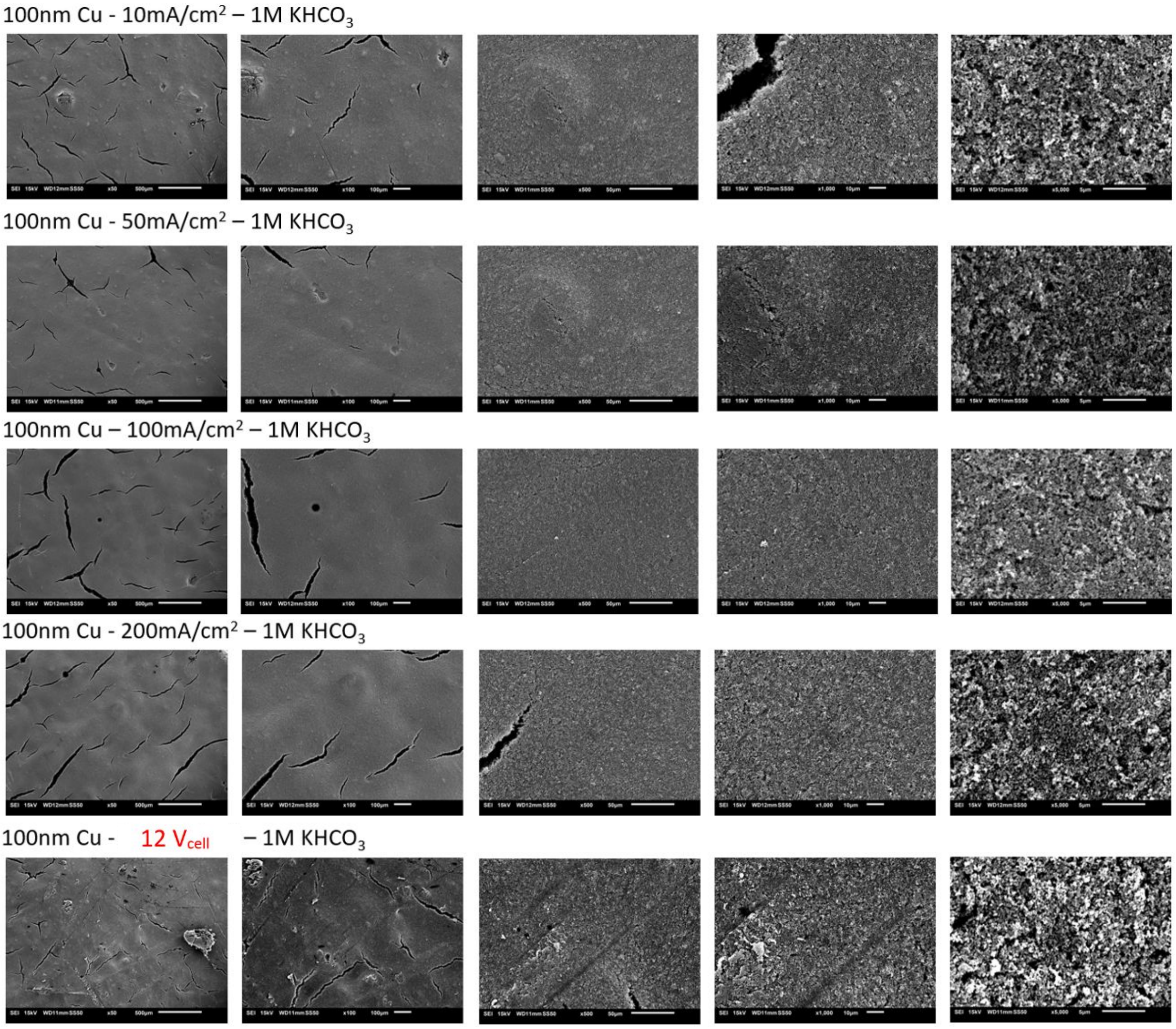

Figure S32. SEM images of 100 nm Cu on GDE after 1 hour reaction in 1 M $KHCO_3$ at various current densities. Throughout all experiments no significant changes were found on the surface structure. The darkening in some images is caused by a change of SEM brightness settings.

High-resolution scanning electron microscopy - HR-SEM

This section contains three magnifications of high resolution scanning electron microscopy (HR-SEM) images of the 5 metals as deposited and after electroreduction for 1 hour in 1 M KOH and 1 M $KHCO_3$ at V_{cell} = 12 V (fig. S33 - 37. Above the images the used magnification can be found. Between the sputtered and used samples differences such as agglomeration, clustering, smoothening, deposition and reformation can be found.

Magnif.	x 15.000	x 50.000	x 150.000
Size	9,0 x 13,5 µm	2,7 x 4,1 µm	0,9 x 1,35 µm

Ag – sputtered

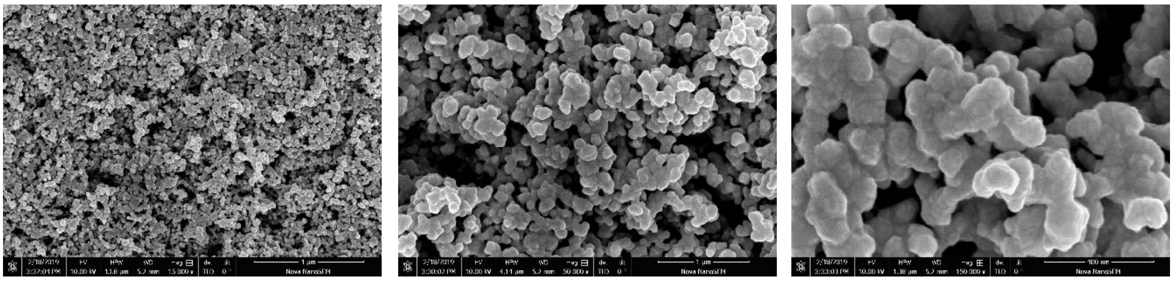

Ag – 1 M KOH

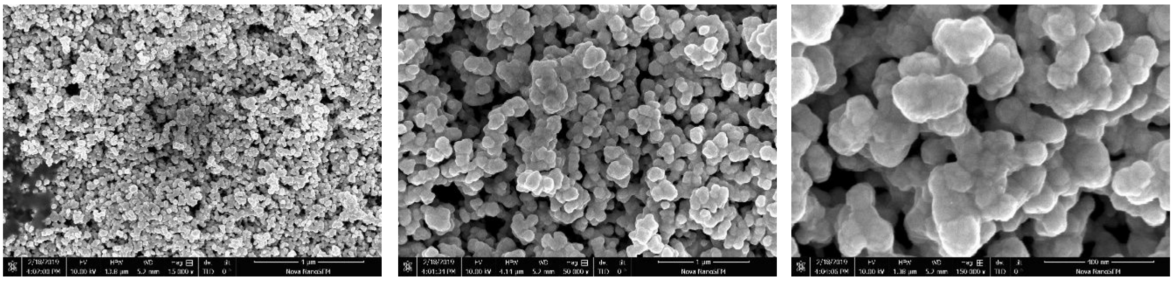

Ag – $KHCO_3$

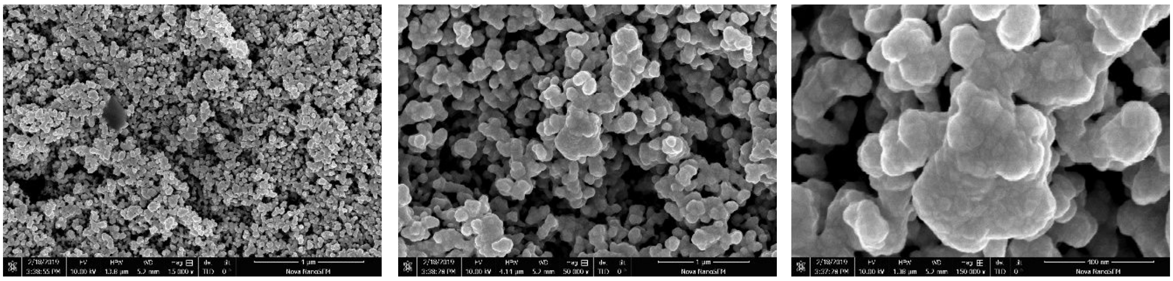

Figure S33. HR-SEM images of 100 nm Ag on GDE before and after 1 hour reaction. The catalyst layer itself seems to be unaffected by the reaction.

Magnif.	x 5.000	x 50.000	x 150.000
Size	27 x 41 µm	2,7 x 4,1 µm	0,9 x 1,35 µm

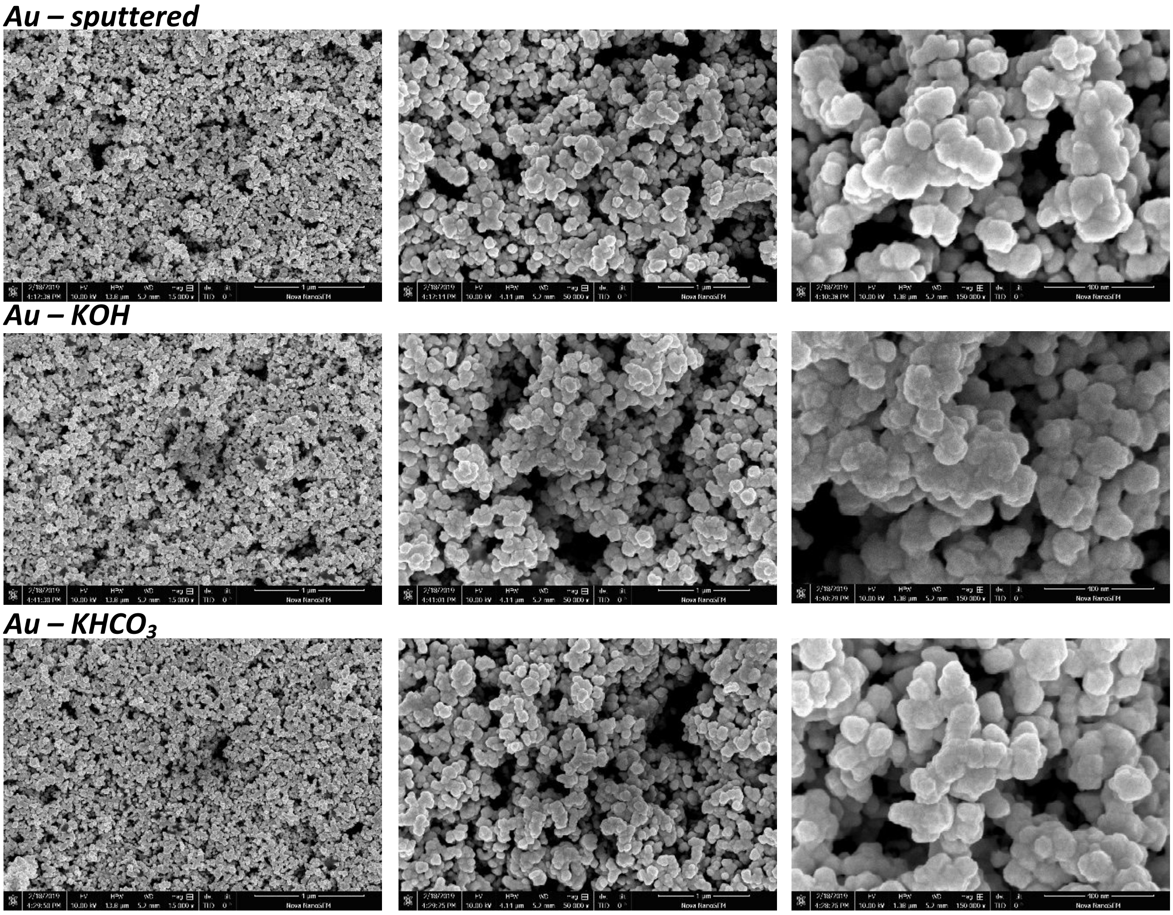

Figure S34. HR-SEM images of 100 nm Au on GDE before and after 1 hour reaction.

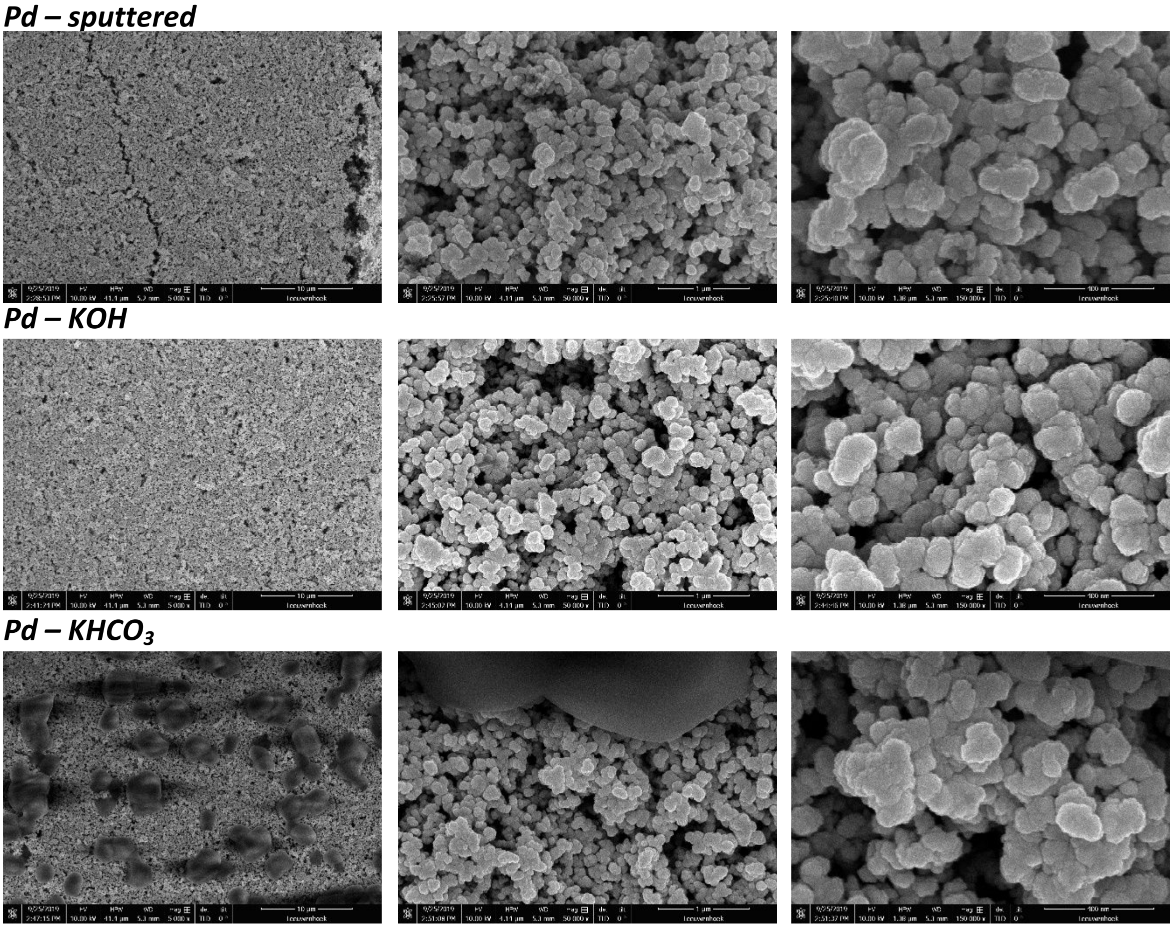

Figure S35. HR-SEM images of 100 nm Pd on GDE before and after 1 hour reaction.

Magnif.	x 5.000	x 50.000	x 150.000
Size	27 x 41 µm	2,7 x 4,1 µm	0,9 x 1,35 µm

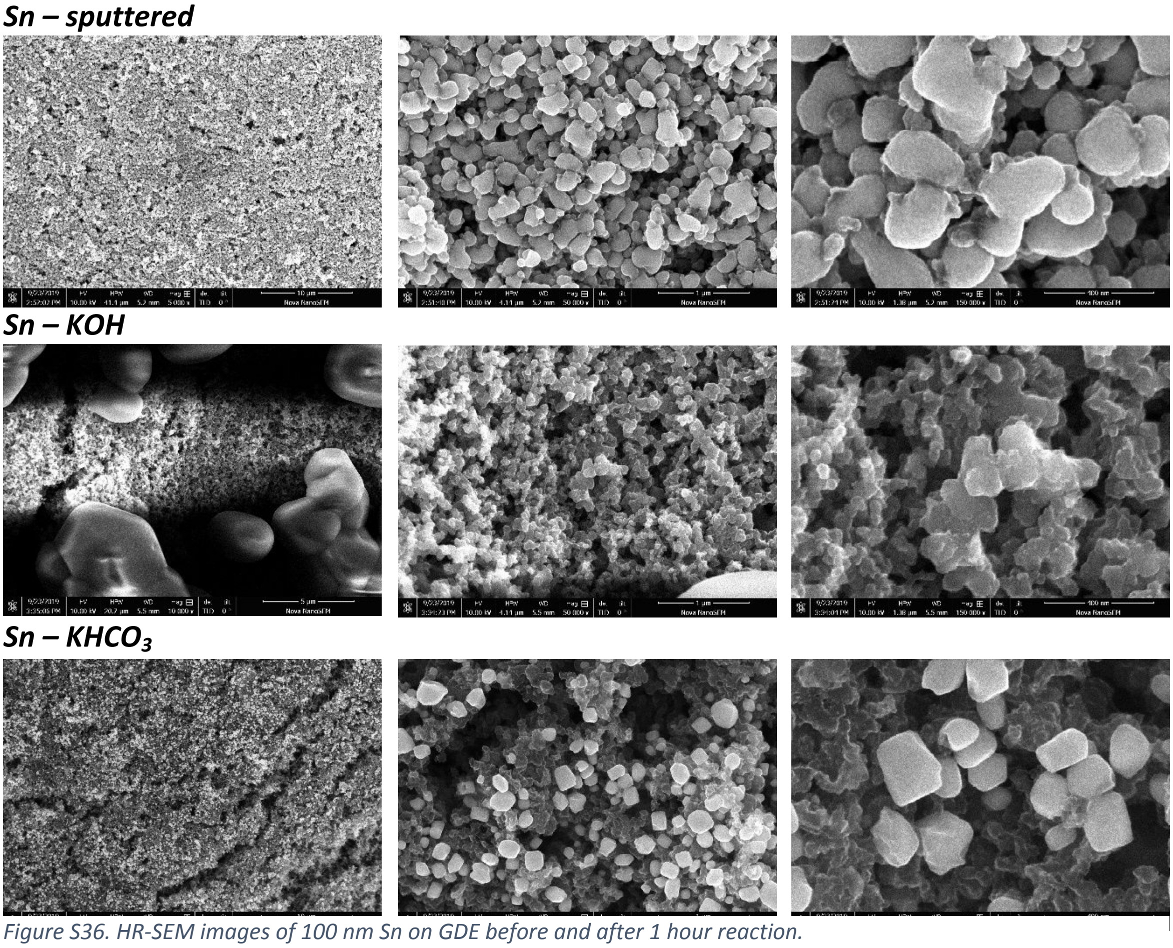

Figure S36. HR-SEM images of 100 nm Sn on GDE before and after 1 hour reaction.

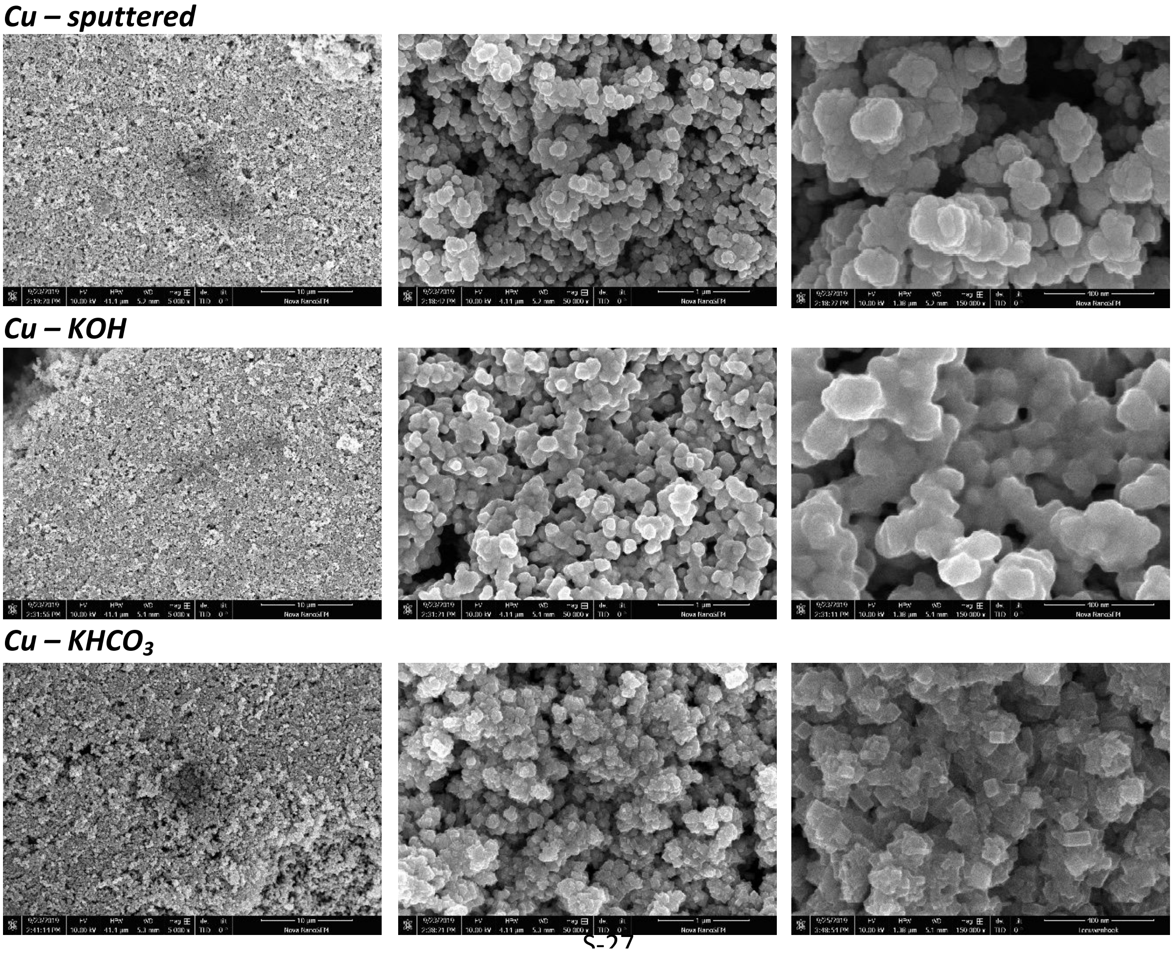

Figure S37. HR-SEM images of 100 nm Cu on GDE before and after 1 hour reaction.

X-ray photoelectron spectroscopy - XPS

This section contains all XPS scans of the 5 metals as deposited and after electroreduction at 200 mA/cm^2 for 1 hour in both KOH and $KHCO_3$ (fig. S38 – S52). During XPS the following scans were performed for each of these samples:

1. **A survey scan** between 1350 and 0 eV
2. **A respective metal element scan** (Ag/Au/Pd/Sn/Cu)
3. **A valence band scan** [in some cases]
4. **A carbon (C) scan** between 298 and 279 eV
5. **An oxygen (O) scan** between 545 and 525 eV
6. **A potassium (K) scan** between 305 and 287 eV

Corresponding to the numbers above the following format is used:

[Metal] [Condition]

**1.**	**2.**	**[3.]**
**4.**	**5.**	**6.**

The survey and respective metal elements give us information on the stability of the metal, for instance, in the Sn fresh vs Sn KOH/$KHCO_3$ case there is a significant signal drop-off of the Sn3d peak (~99% for KOH, ~50% for $KHCO_3$) after testing, indicating its instability.

To obtain additional information on the presence and stability of the GDE the carbon (and fluorene) peaks are looked into. Each scan is performed on and averaged over 2 separate locations (random spots, not on substrate native crevices).

Ag fresh

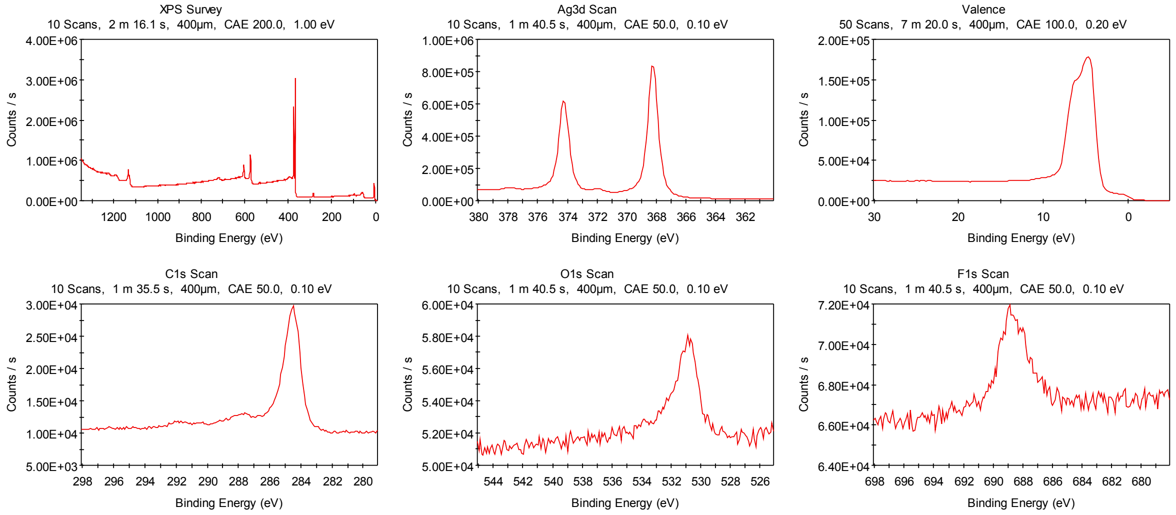

Figure S38. XPS spectra of a fresh Ag sample.

Ag KOH

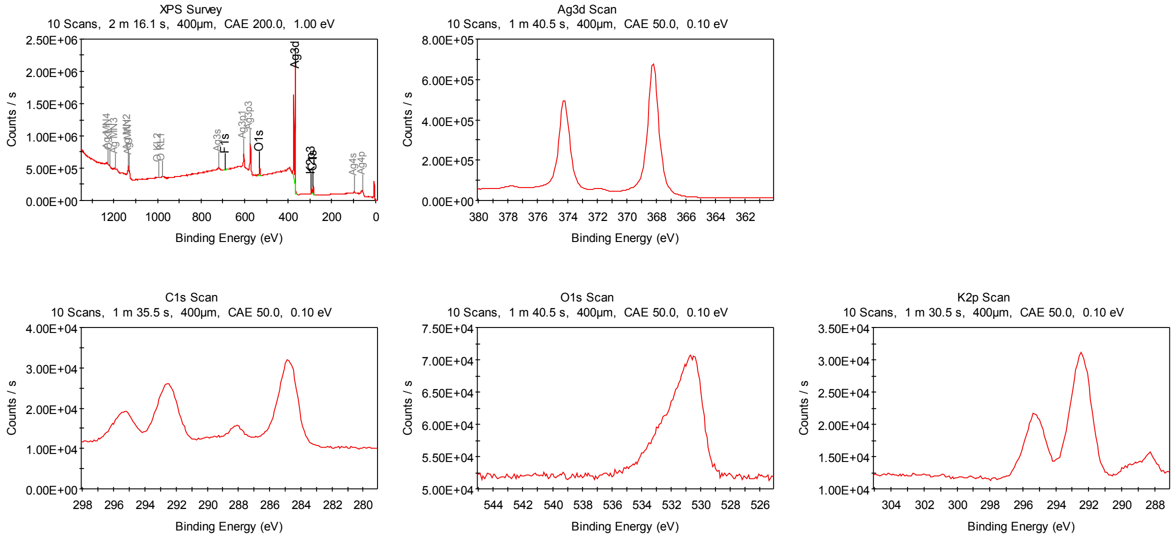

Figure S39. XPS spectra of a Ag sample after 1 hr electrolysis at 200 mA cm^{-2} in 1 M KOH.

Ag KHCO$_3$

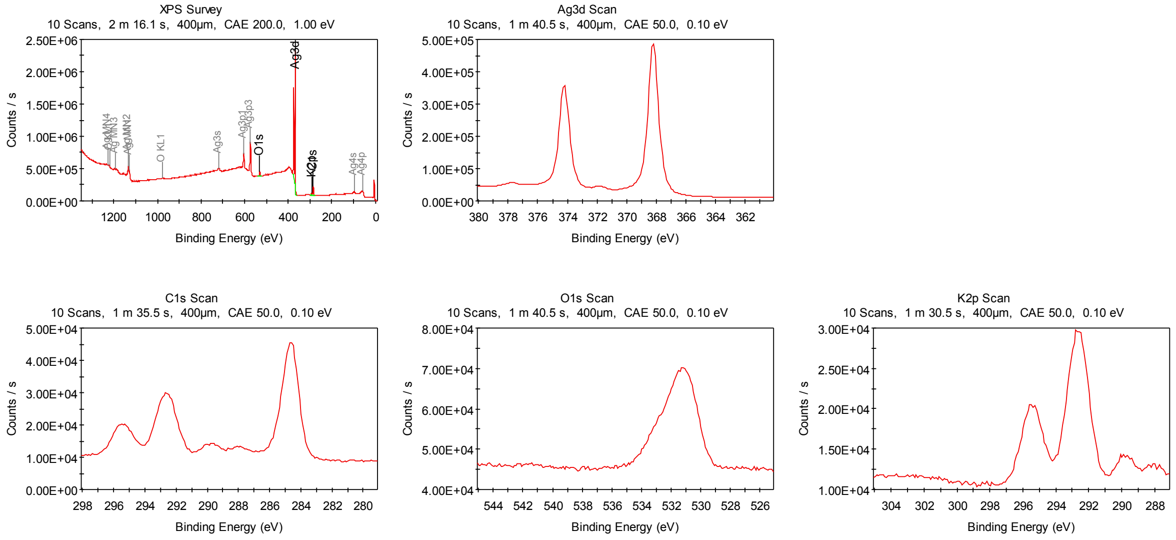

Figure S40. XPS spectra of a Ag sample after 1 hr electrolysis at 200 mA cm^{-2} in 1 M KHCO$_3$.

Au fresh

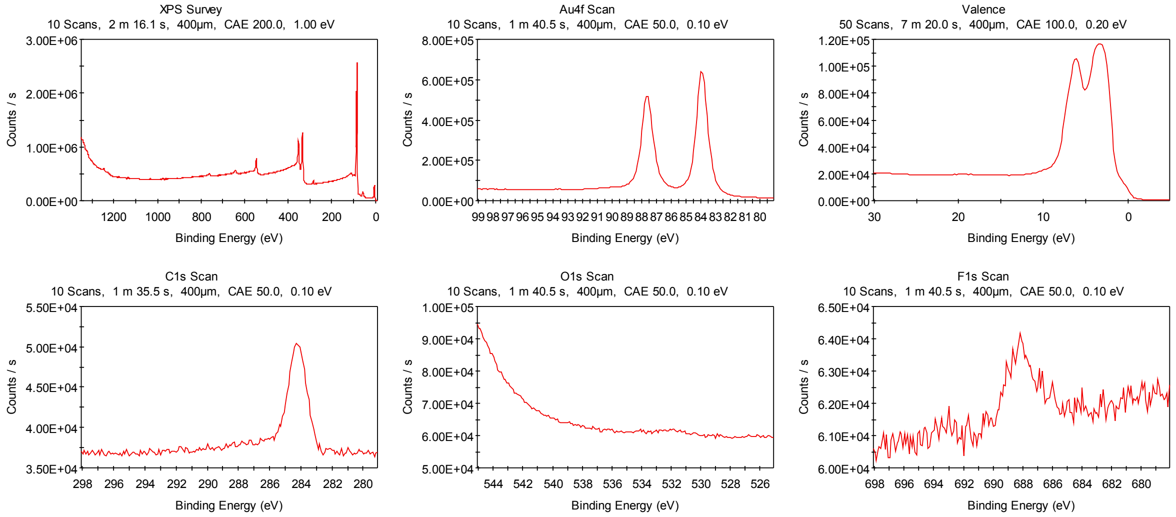

Figure S41. XPS spectra of a fresh Au sample.

Au KOH

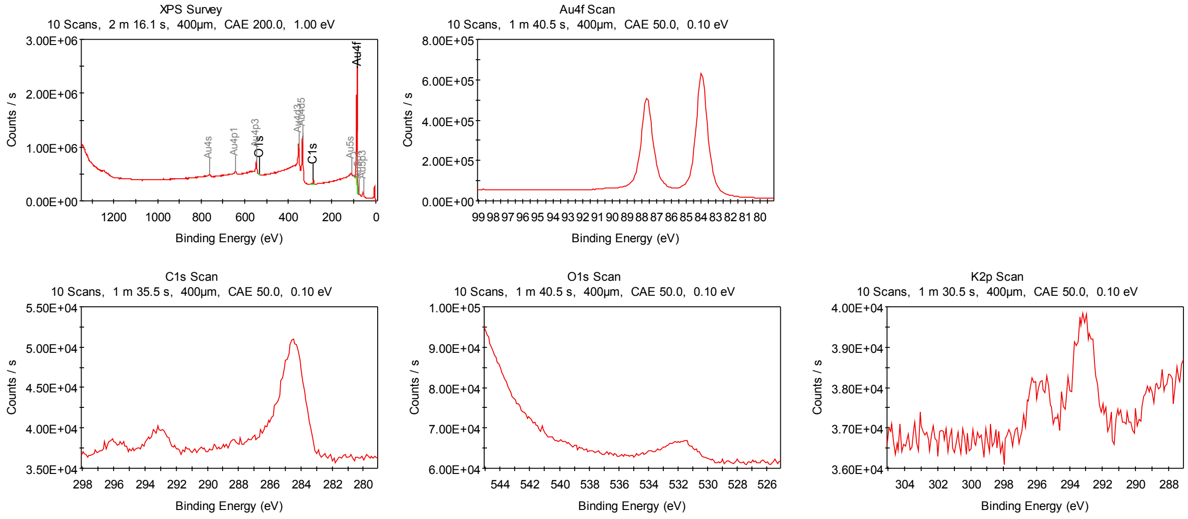

Figure S42. XPS spectra of a Au sample after 1 hr electrolysis at 200 mA cm^{-2} in 1 M KOH.

Au KHCO$_3$

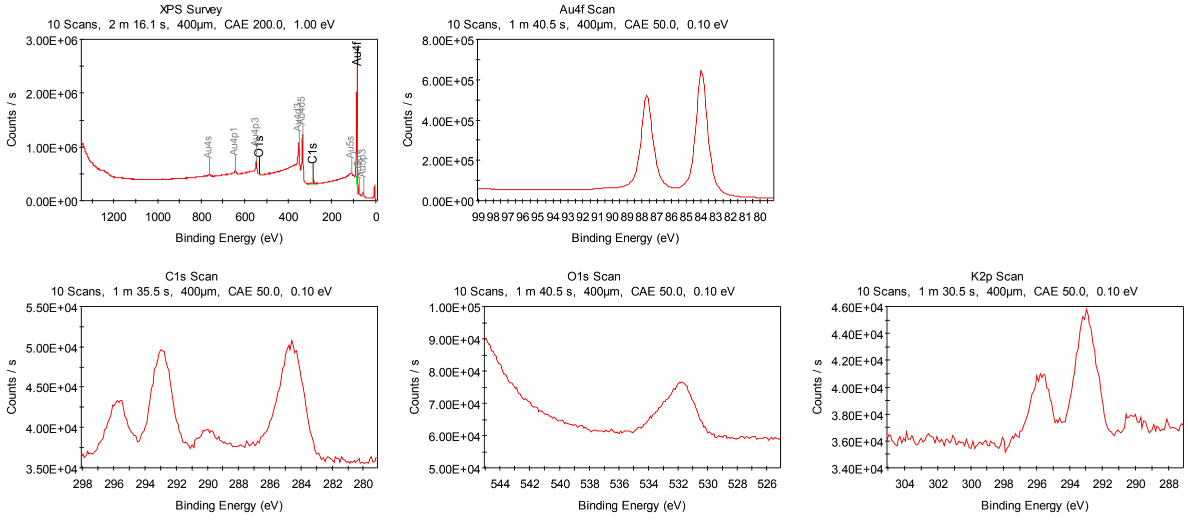

Figure S43. XPS spectra of a Au sample after 1 hr electrolysis at 200 mA cm^{-2} in 1 M KHCO$_3$.

Pd fresh

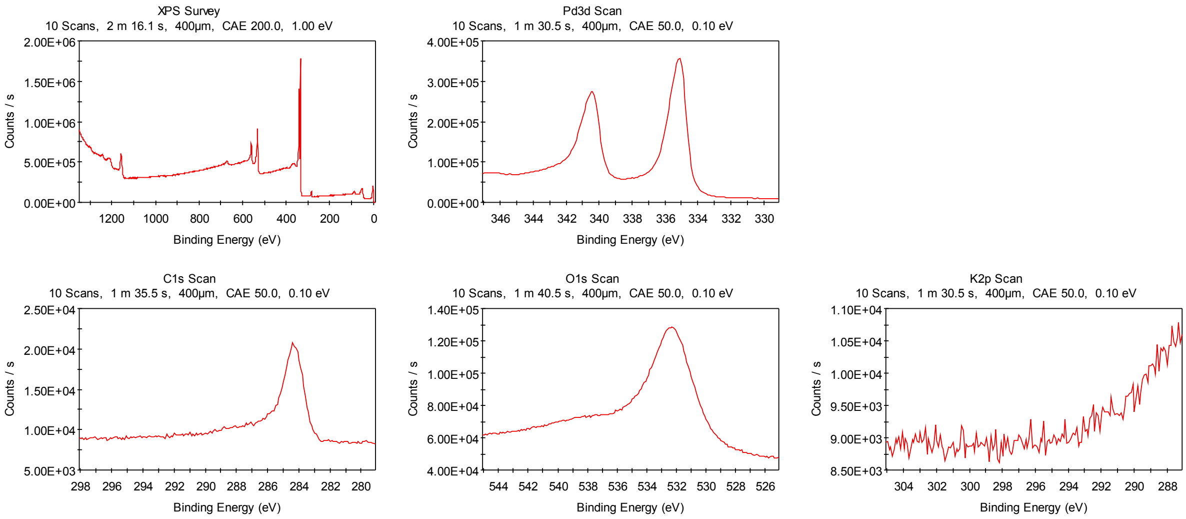

Figure S44. XPS spectra of a fresh Pd sample.

Pd KOH

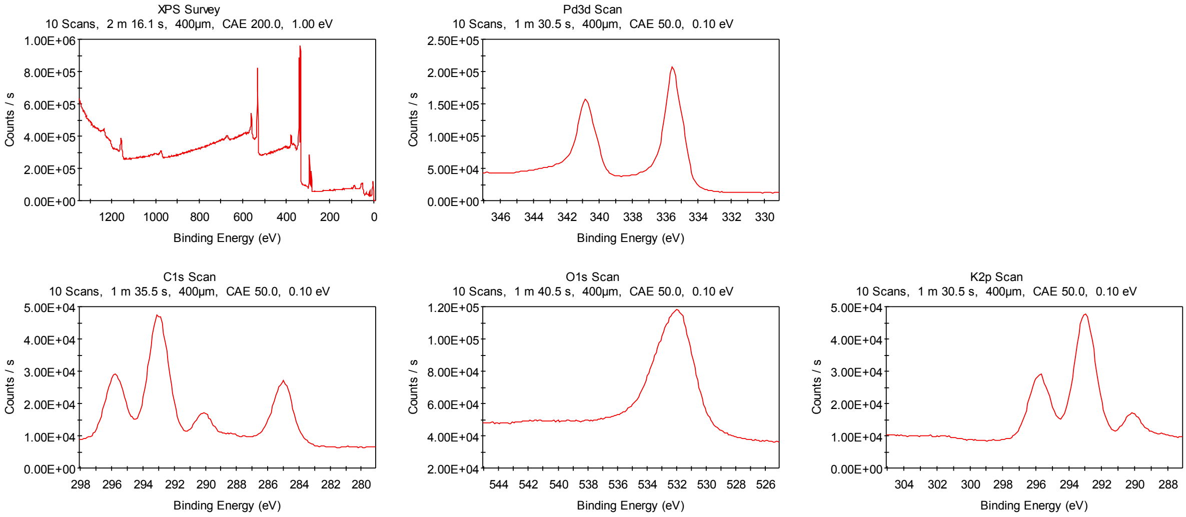

Figure S45. XPS spectra of a Pd sample after 1 hr electrolysis at 200 mA cm^{-2} in 1 M KOH.

Pd $KHCO_3$

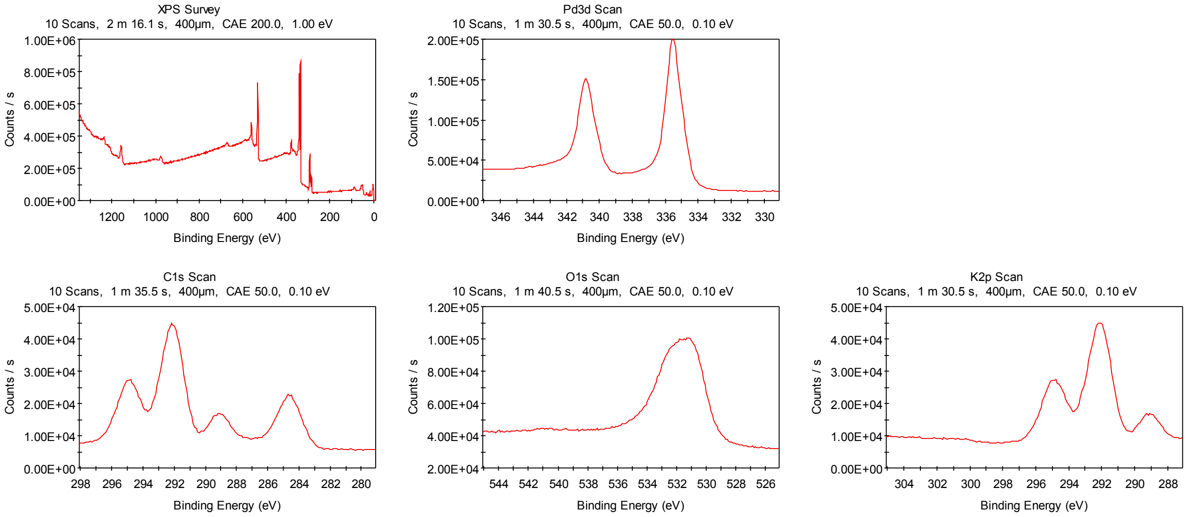

Figure S46. XPS spectra of a Pd sample after 1 hr electrolysis at 200 mA cm^{-2} in 1 M $KHCO_3$.

Sn fresh

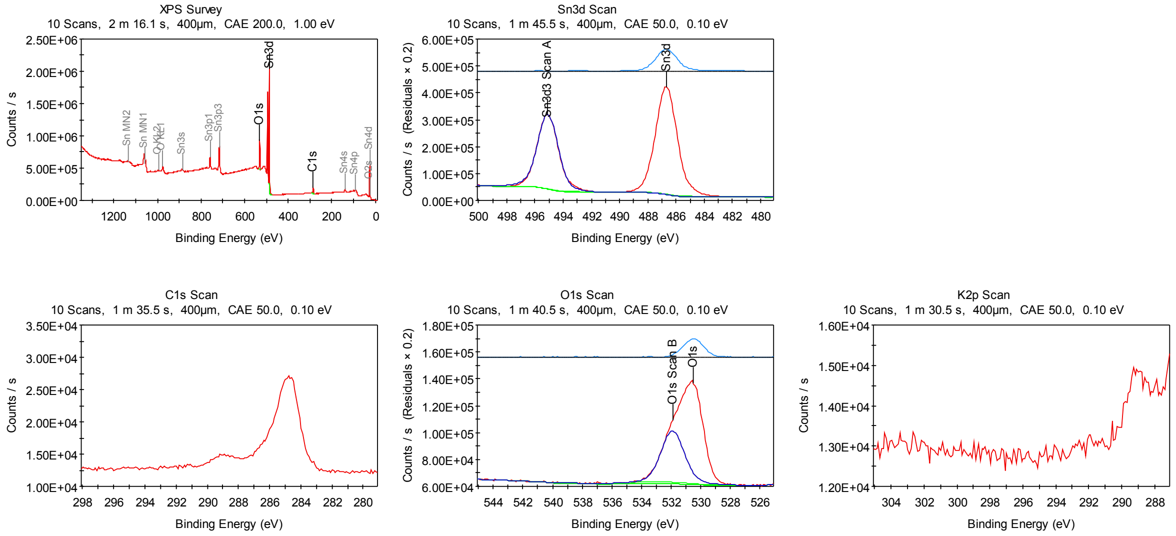

Figure S47. XPS spectra of a fresh Sn sample.

Sn KOH

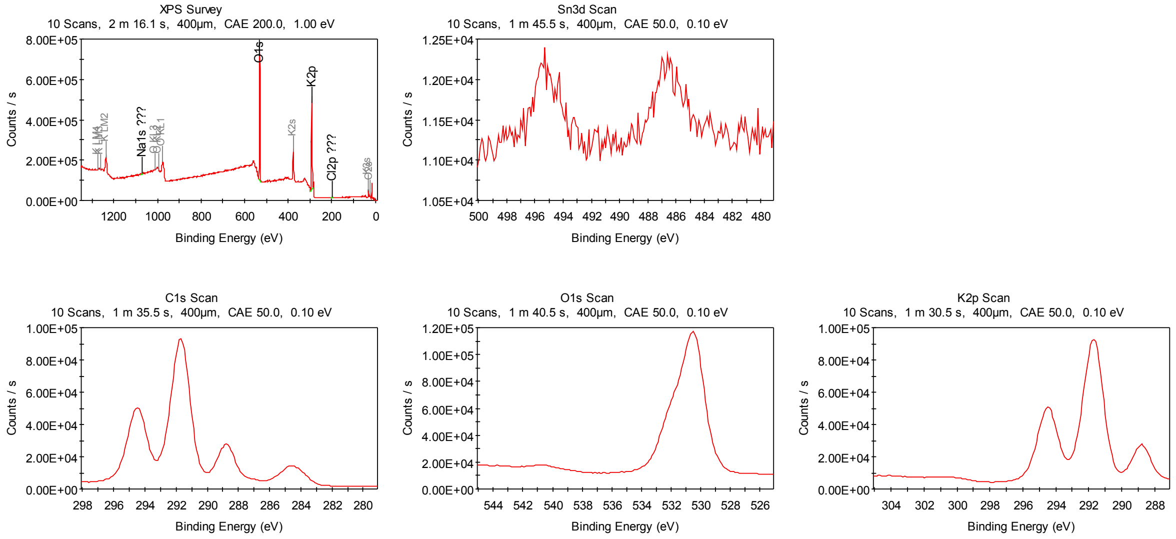

Figure S48. XPS spectra of a Sn sample after 1 hr electrolysis at 200 mA cm^{-2} in 1 M KOH.

Sn KHCO$_3$

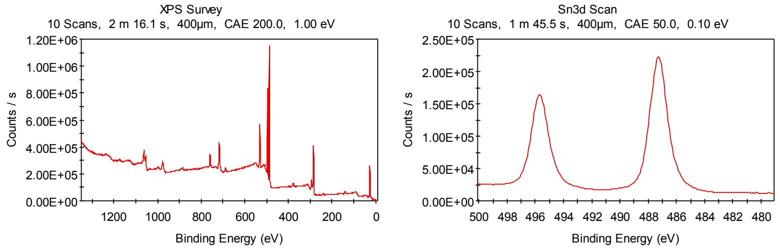

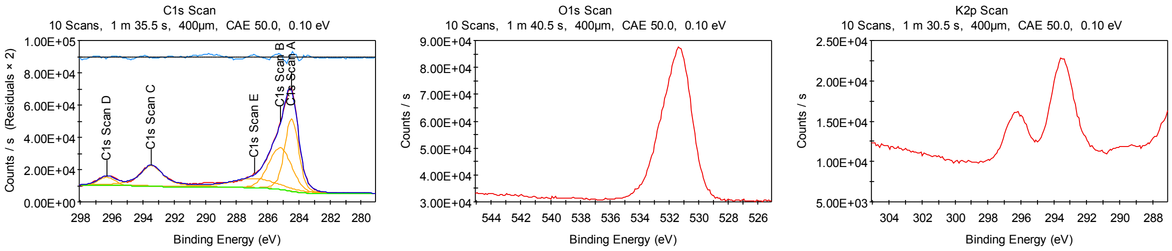

Figure S49. XPS spectra of a Sn sample after 1 hr electrolysis at 200 mA cm^{-2} in 1 M $KHCO_3$.

Cu fresh

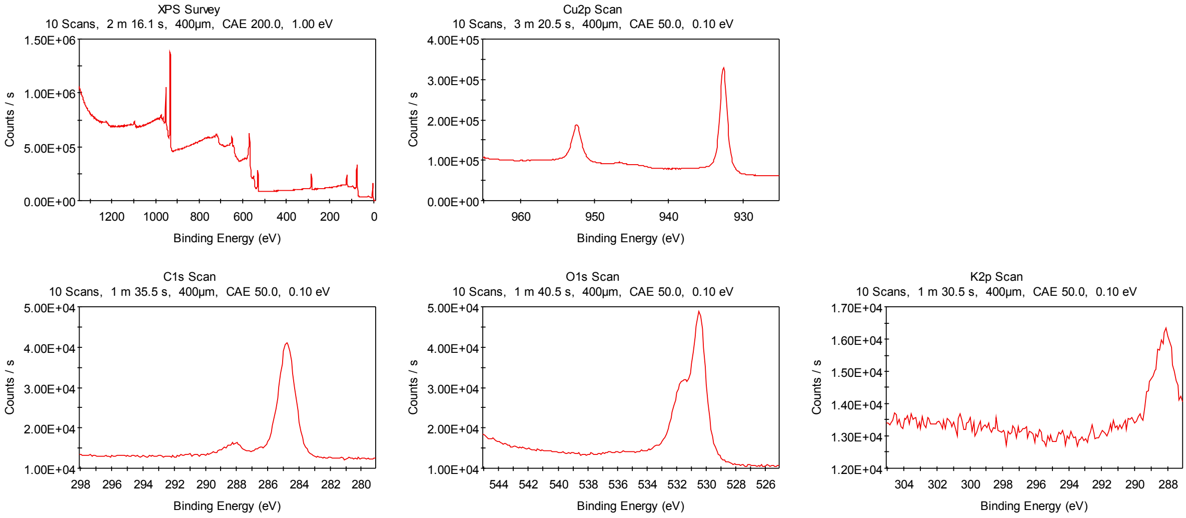

Figure S50. XPS spectra of a fresh Cu sample.

Cu KOH

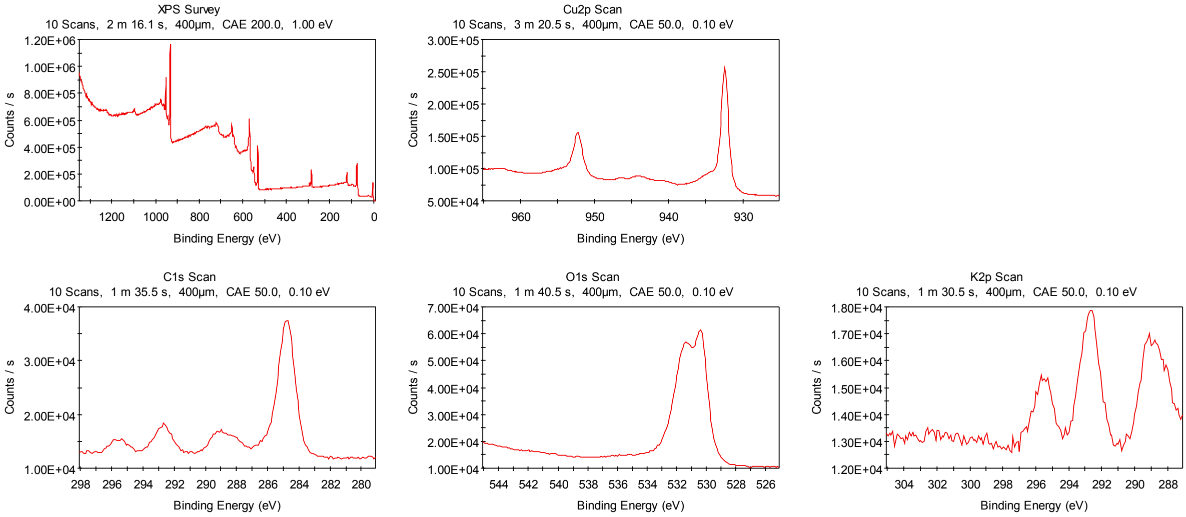

Figure S51. XPS spectra of a Cu sample after 1 hr electrolysis at 200 mA cm^{-2} in 1 M KOH.

Cu $KHCO_3$

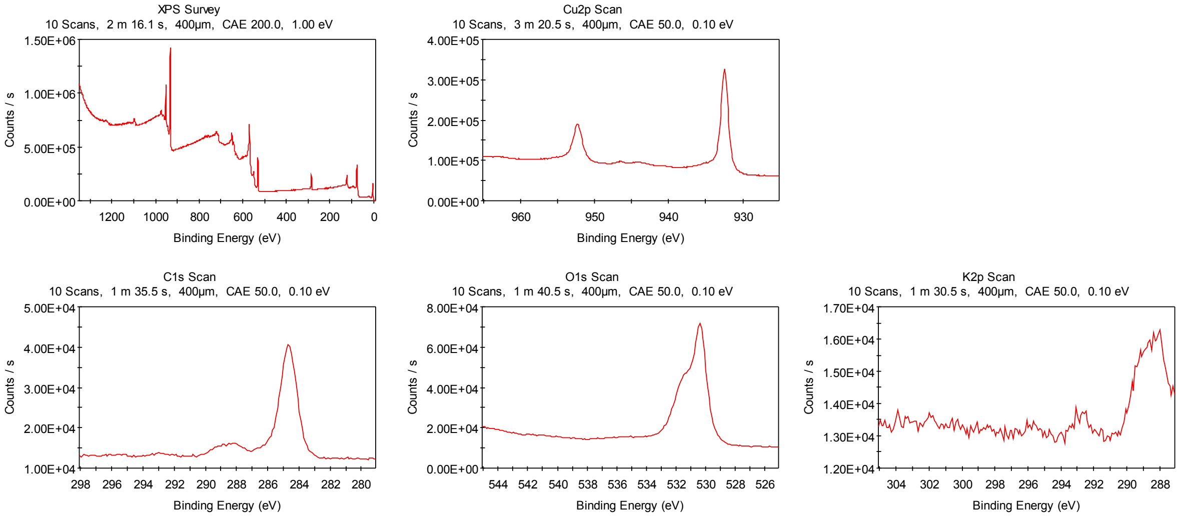

Figure S52. XPS spectra of a Cu sample after 1 hr electrolysis at 200 mA cm^{-2} in 1 M $KHCO_3$.

Atomic force microscopy - AFM

Atomic Force Microscopy (AFM) is a powerful tool to look into the smallest details of the reactive surface. Below µm size topography heightmaps of a bare GDE sample can be found as well as of before and after catalysis samples of Ag, Au, Sn and Cu (Fig. S53 – S57). The Z-axis of in the 1 µm x 1 µm heightmaps has an aspect ratio of 1:1:0.3 (the 2 µm x 2 µm 'Bare GDE' sample has a normal 1:1:1 ratio). Although it is hard to compare AFM images, it gives an idea of what the surface looks like on the nano- and microscale.

Bare GDE

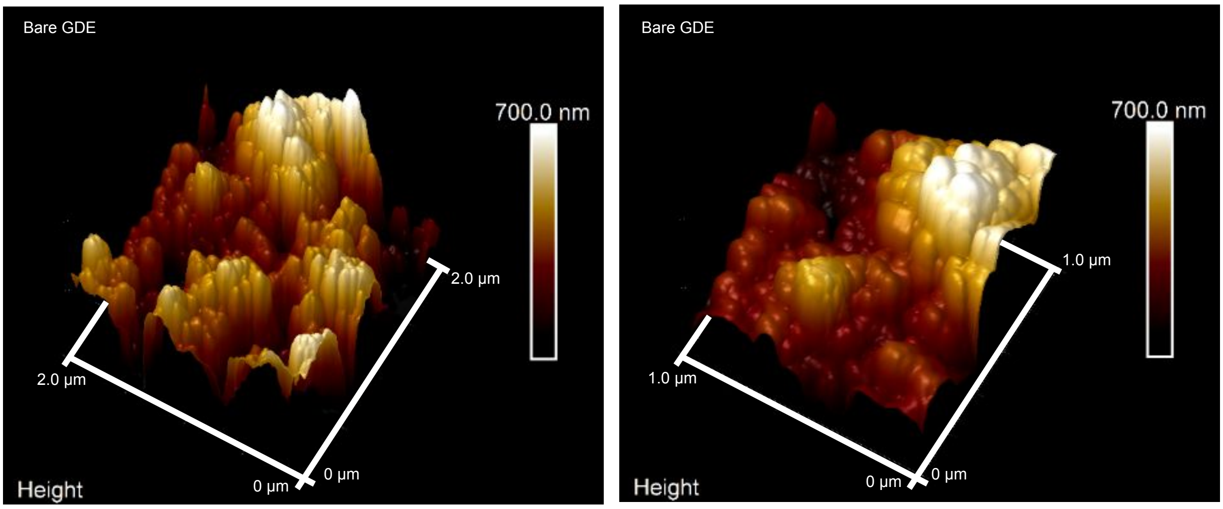

Figure S53. Two AFM images of a bare GDE substrate. The roughness of the surface ranges over 700 nm.

Ag

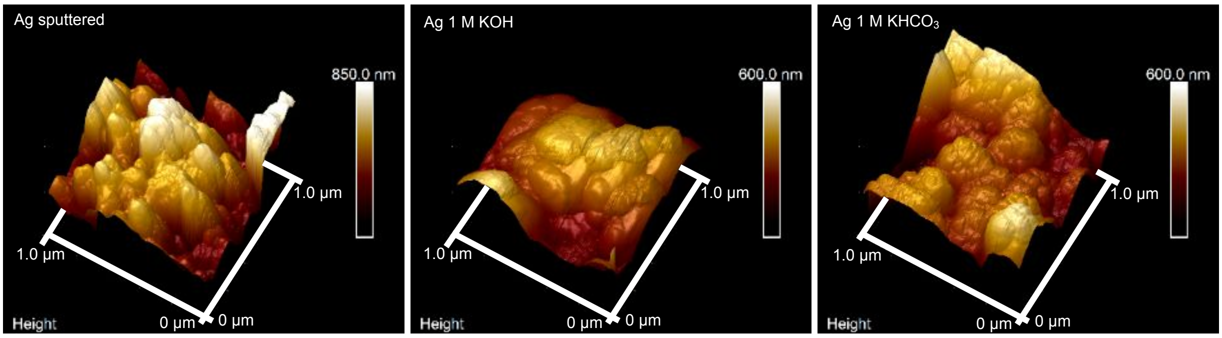

Figure S54. AFM images of Ag before and after electrolysis.

Au

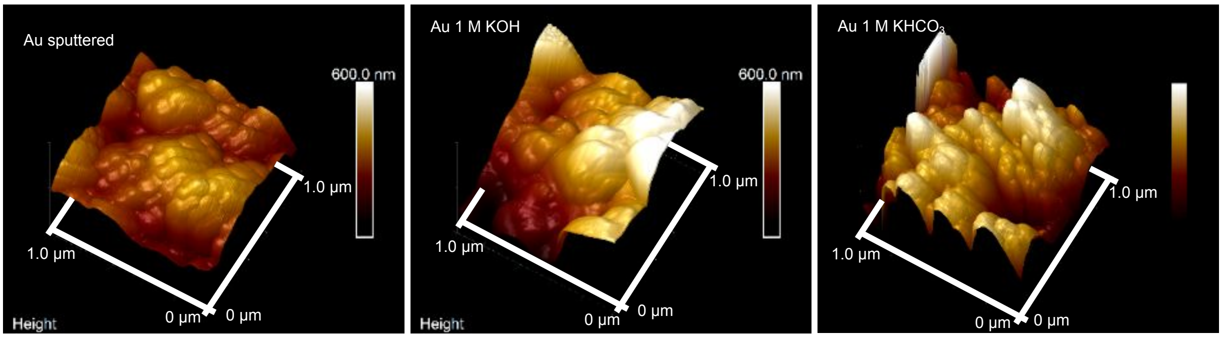

Figure S55. AFM images of Au before and after electrolysis.

Sn

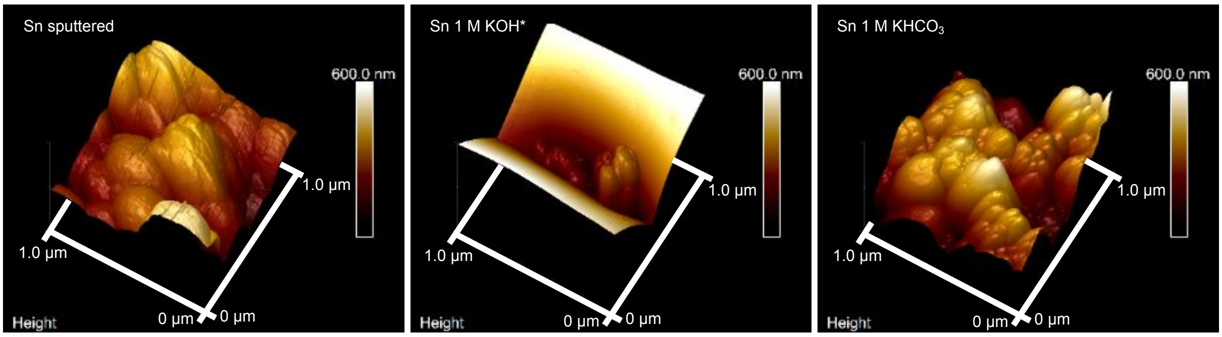

Figure S56. AFM images of Sn before and after electrolysis. For the Sn 1M KOH, SEM images were covered in bright crystals. The KOH AFM measurement was performed in a sharp valley between two crystals where only a small area of the GDE was visible.

Cu

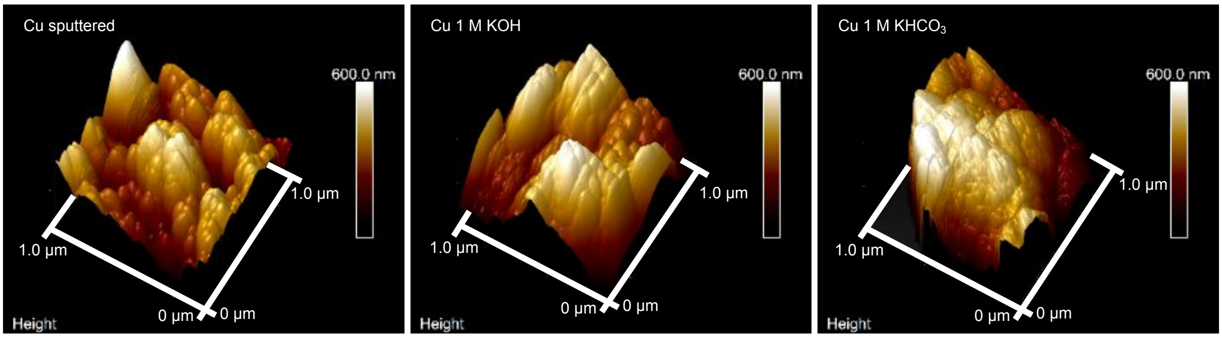

Figure S57. AFM images of Cu before and after electrolysis.

PTFE Cell design

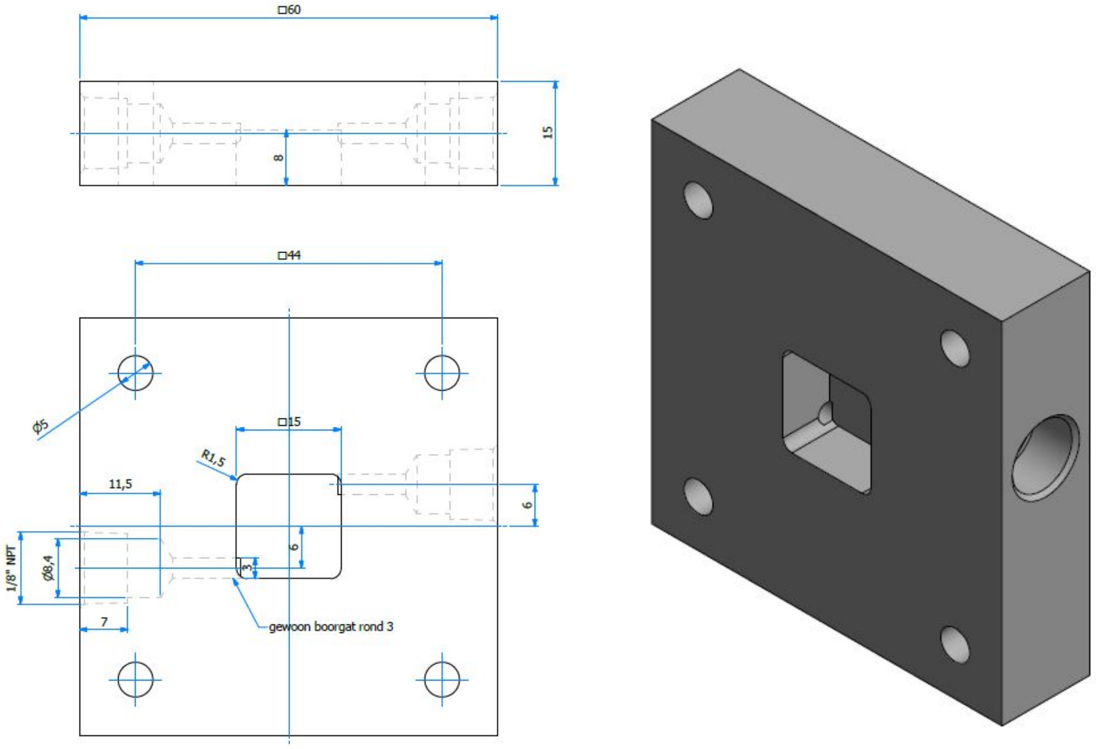

Figure S58 Dimensions of the CO2 gas channel for the electrochemical cell.

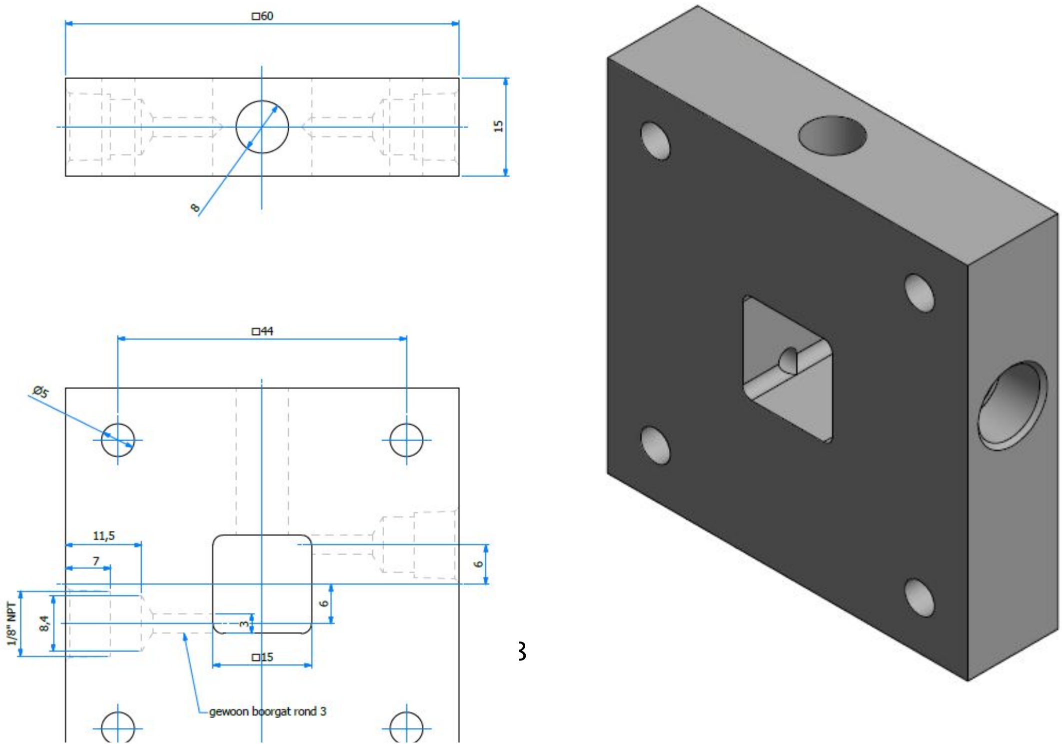

Figure S59 Dimensions of the electrochemical catholyte chamber.

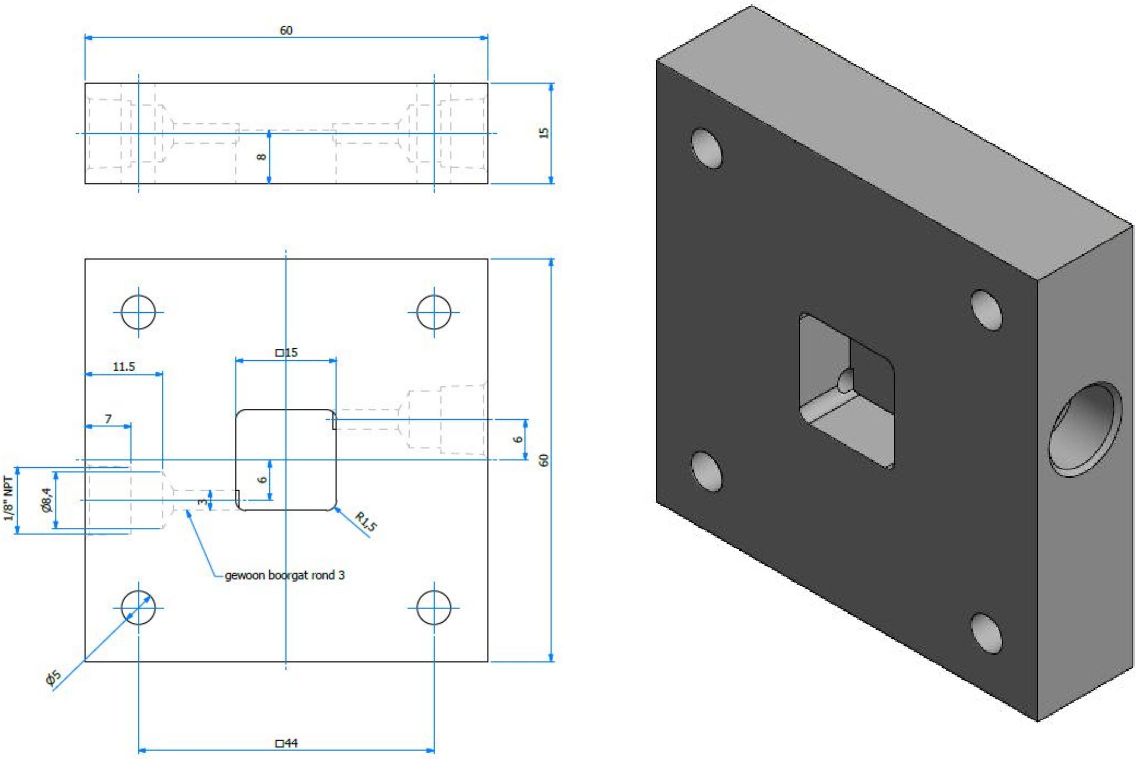

Figure S60 Dimensions of the electrochemical catholyte chamber.

Literature

1. Liu, K., W.A. Smith, and T. Burdyny, *Introductory Guide to Assembling and Operating Gas Diffusion Electrodes for Electrochemical CO2 Reduction.* ACS Energy Letters, 2019. **4**(3): p. 639-643.
